# 2-Unsubstituted Imidazole *N*-Oxides as Novel Precursors of Chiral 3-Alkoxyimidazol-2-ylidenes Derived from *trans*-1,2-Diaminocyclohexane and Other Chiral Amino Compounds †

**DOI:** 10.3390/molecules24234398

**Published:** 2019-12-02

**Authors:** Grzegorz Mlostoń, Małgorzata Celeda, Marcin Jasiński, Katarzyna Urbaniak, Przemysław J. Boratyński, Peter R. Schreiner, Heinz Heimgartner

**Affiliations:** 1 Department of Organic and Applied Chemistry, University of Łódź, Tamka 12, PL-91-403 Łódź, Poland; malgorzata.celeda@chemia.uni.lodz.pl (M.C.); marcin.jasinski@chemia.uni.lodz.pl (M.J.); katarzyna.urbaniak@chemia.uni.lodz.pl (K.U.); 2 Department of Organic Chemistry, Wrocław University of Technology, Wyspiańskiego 27, PL-50-370 Wrocław, Poland; przemyslaw.boratynski@pwr.edu.pl; 3 Justus Liebig University, Institute of Organic Chemistry, Heinrich-Buff-Ring 17, D-35392 Giessen, Germany; prs@uni-giessen.de; 4 Department of Chemistry, University of Zurich, Winterthurerstrasse 190, CH-8057 Zurich, Switzerland; heinz.heimgartner@chem.uzh.ch

**Keywords:** carbene sulfurization, chiral compounds, chiral nucleophilic carbenes, imidazole *N*-oxides, imidazole-2-thiones, *N*-alkoxyimidazolium salts

## Abstract

‘Desymmetrization’ of *trans-*1,2-diaminocyclohexane by treatment with α,ω-dihalogenated alkylation reagents leads to mono-NH_2_ derivatives (‘primary-tertiary diamines’). Upon reaction with formaldehyde, these products formed monomeric formaldimines. Subsequently, reactions of the formaldimines with α-hydroxyiminoketones led to the corresponding 2-unsubstituted imidazole *N*-oxide derivatives, which were used here as new substrates for the in situ generation of chiral imidazol-2-ylidenes. Upon O-selective benzylation, new chiral imidazolium salts were obtained, which were deprotonated by treatment with triethylamine in the presence of elemental sulfur. Under these conditions, the intermediate imidazol-2-ylidenes were trapped by elemental sulfur, yielding the corresponding chiral non-enolizable imidazole-2-thiones in good yields. Analogous reaction sequences, starting with imidazole *N*-oxides derived from enantiopure primary amines, amino alcohols, and amino acids, leading to the corresponding 3-alkoxyimidazole-2-thiones were also studied.

## 1. Introduction

Since 1991, after the isolation of the first stable 1,3-di(adamantyl)imidazol-2-ylidene [1], there has been a wealth of both structural studies and practical applications of nucleophilic *N*-heterocyclic carbenes (NHCs) [2,3]. At the same time, there is continuing demand for catalysts and ligands for asymmetric synthesis [4]. Chiral NHCs are particularly suitable for such applications, and the elaboration of methods for the preparation of their precursors and subsequent applications in asymmetric synthesis are intensively pursued [5,6,7,8]. One efficient method for the preparation of chiral NHCs is the introduction of a chiral fragment attached to the N-atom located within the heterocyclic skeleton (chiral *N*-substituents).

**Scheme 1:**
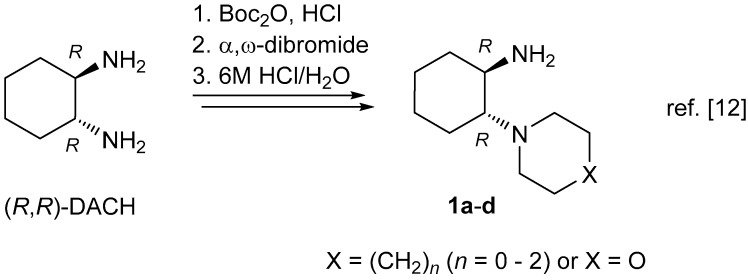
*trans*-1,2-diaminocyclohexane (DACH) and its ‘desymmetrized’ derivatives **1** (Reproduced with permission from [12]).

It is therefore natural that one of the most important motifs in the structure of catalysts and ligands for asymmetric catalysis derives from easily available and inexpensive enantiomerically pure (*R*,*R*)-*trans*-1,2-diaminocylohexane (*trans*-DACH, Scheme 1) [9,10]. Numerous derivatives of this exceptional diamine have been synthesized via functionalization of only one or both amino groups. In recent years, much attention has been paid to so-called bifunctional catalysts, which are prepared by modifications of the amino groups. Derivatives of type **1** with a cyclic amine motif are promising building blocks for the synthesis of new bifunctional catalysts [11,12] and are important synthons for the preparation of some bioactive compounds [13,14,15,16,17]. An efficient method for the ‘desymmetrization’ of DACH, reported only in recent years, comprises two-fold *N*,*N*-alkylation of only one NH_2_ group using α,ω-dihaloalkylating reagents as shown in Scheme 1 [12].

Along with *trans*-DACH, another primary amine widely explored in asymmetric synthesis is α-methylbenzylamine (α-MBA), but to date, reports on its application in the synthesis of chiral NHCs are limited [6]. Similarly, chiral β-amino alcohols and amino esters have scarcely been explored. Selected representatives of these two classes of compounds will also be involved in this study.

**Scheme 2:**
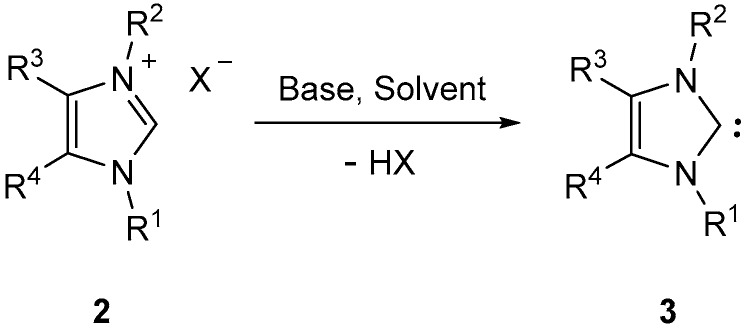
Formation of imidazol-2-ylidenes by deprotonation of imidazolium salts.

It is well established that the most efficient and preparatively useful method for the in situ generation of imidazol-2-ylidenes **3** comprises the deprotonation of the corresponding imidazolium salts of type **2** by treatment with a base (Scheme 2) [2,3].

In our earlier publications, we described an efficient method for the synthesis of enantiomerically pure bis- and mono-imidazole *N*-oxides derived either from *trans*-1,2-diaminocyclohexane [18,19], α-methylbenzylamine [20], β-amino alcohols [21], and amino acid esters [22]. In another study, we demonstrated that 2-unsubstituted imidazole *N*-oxides including some chiral derivatives can be O-alkylated by treatment with alkyl bromides [23]. However, these 1-alkoxyimidazolium salts have never been used for the generation of *N*-alkoxy-substituted imidazol-2-ylidenes of type **3**. In a very recent publication, we reported the synthesis of non-symmetric imidazolium salts prepared in a multistep synthesis starting with adamantyloxyamine [24]. In a test experiment, a symmetric 1,3-di(adamantyloxy)imidazolium salt was converted into the corresponding imidazole-2-thione derivative via an intermediate NHC. Prompted by these results, we decided to synthesize a series of new chiral 1-alkoxyimidazolium salts derived from ‘desymmetrized’ *trans*-1,2-diaminocylohexane derivatives of type **1**, which can subsequently be used as precursors of chiral NHCs. The study was extended by the involvement of α-methylbenzylamine as well as selected amino alcohols and amino acid derivatives.

## 2. Results and Discussion

**Scheme 3:**
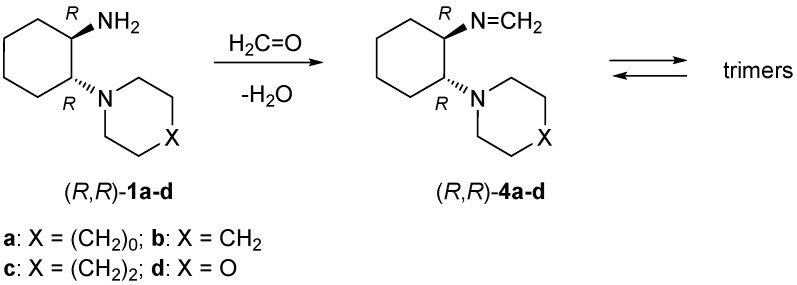
Synthesis of monomeric formaldimines **4** from ‘primary–tertiary’ diamines **1**.

In contrast to DACH, which reacts with two equivalents of formaldehyde forming a dimeric product identified as a tetraazaeicosane derivative [25], all ‘primary-tertiary’ diamines **1a**–**d** reacted with formaldehyde yielded the expected formaldimines **4a**–**d** as solid products (Scheme 3). In the solid state they exist as hexahydro-1,3,5-triazines (trimers), which in polar, aprotic solvents undergo almost total dissociation forming the monomeric imines. Similar observations were made for cyclohexylformaldimine [26]. For example, formaldimine **4b** dissolved in CDCl_3_ dissociates with t_1/2_ of ca. 30 s at 298 K. On the other hand, the same imine observed in the C_6_D_6_ solution exists as an equilibrium mixture of trimeric and monomeric forms in a ratio of ca. 2.5:1. The structures of formaldimines **4** were confirmed by ^1^H and ^13^C-NMR spectra in CDCl_3_. For example, in the ^1^H-NMR spectrum of **4b**, the characteristic AB-system of the monomeric form located at 7.13 and 7.28 ppm with *J* = 18 Hz was attributed to the N=CH_2_ group. In the ^13^C-NMR spectrum, the signal of this group appeared at 151.0 ppm, and similar data were found for all formaldimines **4**. However, in all samples, the presence of trimeric forms interfered with the integration of signals. The presence of trimers was revealed by signals of the CH_2_ groups located at 3.80–4.00 ppm. The products were unstable in solution and underwent gradual decomposition over a period of weeks during storage at room temperature. The molecular formulae of the stable, crystalline samples were confirmed by elemental analysis.

**Scheme 4:**
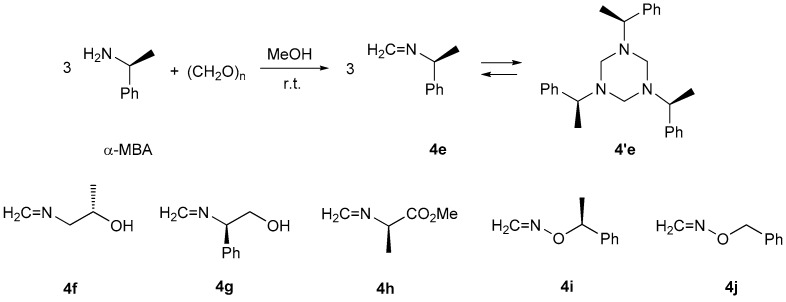
Trimerization of formaldimine **4e** derived from (*S*)-α-methylbenzylamine (α-MBA) [20] (Reproduced with permission from [20]) and structures of analogous formaldimines **4f**–**i**.

In the case of **4e** derived from (*S*)-α-methylbenzylamine ((*S*)-α-MBA), rapid trimerization led to hexahydro-1,3,5-triazine **4′e** (Scheme 4) in the course of its synthesis, and after isolation, it could be used for further transformations only in this form [20]. An analogous tendency for trimerization was also observed for **4f**–**h** used in the study.

In contrast, the alkoxyformaldimine **4i** derived from (*S*)-α-methylbenzyloxyamine (α-MBOA) [27] exists in CDCl_3_ solution in monomeric form, and in that case, no tendency to undergo trimerization was observed even after three days at room temperature. Moreover, a similar behavior was observed in the case of benzyloxyformaldimine (**4j**) prepared from benzyloxyamine (BOA) [28] and formaldehyde in the course of the present study. Apparently, the presence of an alkoxy moiety reduces the electrophilicity of the =CH_2_ unit and thereby formation of the trimeric forms is substantially disfavored.

**Scheme 5:**
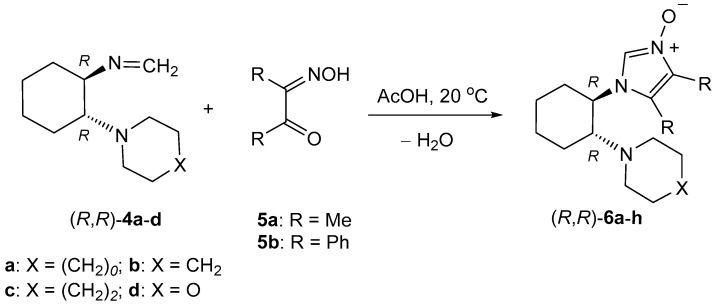
Synthesis of chiral (*R*,*R*)-configured imidazole *N*-oxides **6a**–**h** (see also Table 1).

Enantiomerically pure, (*R*,*R*)-configured 2-unsubstituted imidazole *N*-oxides **6** used in this study as the key building blocks were prepared from enantiopure formaldimines **4**, derived from the corresponding amines **1**, and α-hydroxyiminoketones **5a**,**b** in glacial acetic acid at room temperature [24]. In the case of **4b**, along with the (*R*,*R*)-configured stereoisomer, the (*S*,*S*)-enantiomer was also involved in the study (Scheme 5 and Table 1).

Reactions of **4** with α-hydroxyiminoketones **5** leading to imidazole *N*-oxides **6** were performed at room temperature in glacial acetic acid. After isolation, the products were identified by spectroscopic methods, and the most characteristic signal of HC(2) of the imidazole ring in the ^1^H-NMR spectra appeared at ca. 8 ppm. Products **6** were alkylated with an equimolar amount of benzyl bromide in CH_2_Cl_2_ solution at room temperature. Due to the observed slow decomposition, the (*N*-benzyloxy)imidazolium salts **7** were used for the next reaction step without further purification. In the case of the azepane derivative **7e**, the structure of the crude product was confirmed by ^1^H-NMR spectroscopy. The characteristic HC(2)-signal was significantly shifted toward the lower field and appeared at 11.35 ppm. In addition, chemoselective *O*-benzylation was confirmed by the appearance of only one AB-system (5.67 and 5.72 ppm, *J* = 12.0 Hz) of the OCH_2_Ph group. Thus, competitive N-benzylation could be ruled out.

**Scheme 6:**
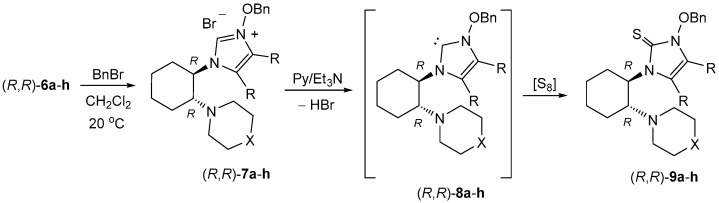
Preparation of imidazole-2-thiones **9a**–**h** bearing a chiral substituent via intermediate imidazol-2-ylidenes **8** (see also Table 1).

The obtained crude imidazolium salts **7** were used as precursors of chiral imidazol-2-ylidenes **8** (Scheme 6). Their intermediacy was proven with known trapping reactions with elemental sulfur [24,29,30] leading to non-enolizable imidazole-2-thiones. The deprotonation of **7** was easily achieved by treatment with triethylamine in pyridine. In the presence of elemental sulfur, the in situ generated NHCs **8** exclusively reacted with the desired imidazole-2-thiones **9** (Scheme 6 and Table 1). Their structures were confirmed by spectroscopic data with the most characteristic signal in the ^13^C-NMR spectra being the C(2)=S group resonating between 156 and 162 ppm. The obtained imidazole-2-thiones **9a**–**h** were shown to be optically active compounds. The optical rotation for all these products was determined in CHCl_3_ solutions and no racemization under the reaction conditions is expected.

The inspection of the ^1^H-NMR spectra of **9** in CDCl_3_ solution at room temperature evidenced complex signal patterns. It is likely that there are equilibria of different rotamers and/or conformers existing in these solutions. To acquire more information, the ^1^H-NMR spectra of analytically pure (*R*,*R*)-**9b** were recorded at different temperatures in a 1,1,2,2-tetrachloroethane solution. The spectrum at 294 K showed a set of broadened signals in the region of 4.2–4.8 ppm attributed to the PhC*H***_2_**O fragment. In addition, two broad signals of H(C1) and HC(2) of the cyclohexane ring were found at 2.8 and 3.1 ppm. In the spectrum measured at 334 K, the benzylic region revealed one broad signal at 4.6 ppm and the signals of two distinct cyclohexane H-atoms were shifted high-field and overlap with the α-H-atoms of the pyrrolidine ring. This observation supports our assumption of the presence of a dynamic equilibrium of different conformers of (*R*,*R*)-**9b**. An analogous explanation is valid for all spectra of **9** bearing two Ph groups at C(4) and C(5) of the imidazole ring.

**Scheme 7:**
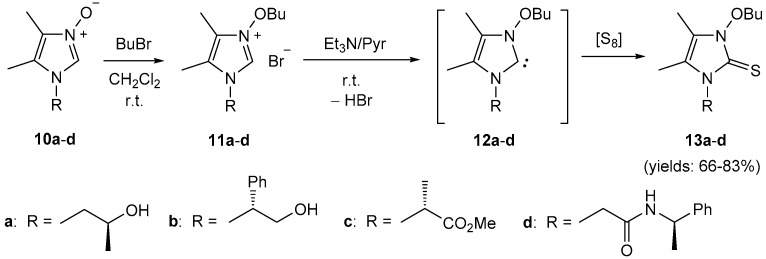
Synthesis of optically active 3-butoxyimidazole-2-thiones **13**.

In extension of the studies performed with ‘desymmetrized’ *trans*-DACH derivatives, analogous experiments were performed starting with enantiomerically pure functionalized primary amines. Thus, the corresponding formaldimines **4f**–**h** (Scheme 4) were converted into imidazole *N*-oxides **10a**–**c** by reaction with the α-hydroxyiminoketone **5a** under standard conditions (see Experimental Section). In addition, enantiopure **10d**, readily available by aminolysis of the corresponding ester with (*R*)-α-MBA [22] was used. The obtained imidazole *N*-oxides **10** were used for the preparation of imidazolium salts **11a**–**d**, which upon treatment with triethylamine in pyridine in the presence of elemental sulfur gave the optically active imidazole-2-thiones **13a**–**d** in good yields (Scheme 7). In these reactions, optically active *N*-butoxyimidazol-2-ylidenes of type **12** were the in situ generated reactive intermediates.

In contrast to **9b**,**d**,**f**, and **g** derived from (*R*,*R*)-*trans*-DACH, the ^1^H-NMR spectra of **13** clearly indicated the presence of a single form in the solution. For example, the spectrum of compound **13b** showed only one multiplet for the CH_2_O unit located at 4.34–4.41 ppm, and two characteristic singlets of the Me groups at C(4) and C(5) of the imidazole ring at 1.72 and 2.09 ppm, respectively. In the ^13^C-NMR spectrum, the signal of the C=S group appeared at 157.4 ppm.

**Scheme 8:**
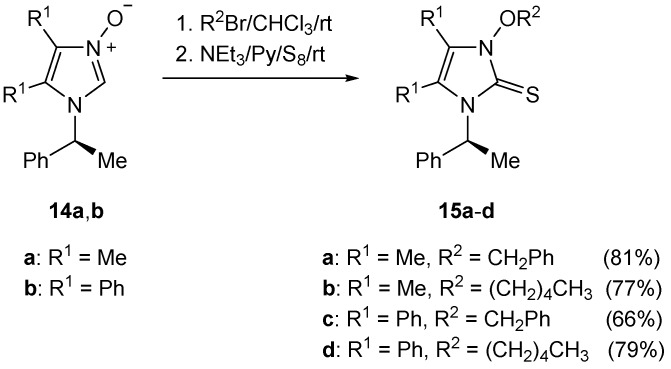
3-Alkoxyimidazole-2-thiones **15a**–**d** derived from (*S*)-α-methylbenzylamine.

In order to check whether steric hindrance is the reason for the existence of different conformers of **9**, four other compounds of that type (i.e., **15a**–**d** derived from (*S*)-α-methylbenzylamine) were synthesized following the multistep procedure presented in Scheme 6 and Scheme 7. The starting 2-unsubstituted imidazole *N*-oxides **14a**,**b** are known compounds [20]. Upon treatment with alkylating reagents (benzyl bromide or *n*-pentyl bromide), they were converted into the corresponding imidazolium salts (Scheme 8). The latter products without purification were treated with triethylamine in the presence of elemental sulfur, yielding the desired **15a–d** in good yields (66–81%).

In that case, the less bulky (*S*)-α-methylbenzyl residue was placed at the N(1) atom. The ^1^H-NMR spectrum of **15a** reveals the presence of the expected AB system of the PhC*H*_2_O group at 5.45 and 5.58 (*J* = 10 Hz) ppm. This result emphasizes the importance of the steric demand in the series of compounds **9** derived from bulky *trans*-1,2-diaminocyclohexane.

## 3. Conclusions

Enantiomerically pure desymmetrized derivatives of *trans*-1,2-diaminocyclohexane containing one free NH_2_ group (i.e., so called ‘primary-tertiary amines’) can be efficiently converted into corresponding formaldimines. The initially obtained formaldimines were transformed into 2-unsubstituted imidazole *N*-oxides, which upon treatment with benzyl bromide were selectively converted into benzyloxyimidazolium salts. After deprotonation with triethylamine, the latter compounds generated new types of chiral imidazol-2-ylidenes. Their appearance was evidenced by the trapping reaction with elemental sulfur leading to chiral non-enolizable imidazole-2-thiones. The same procedure applied to other optically active functionalized primary amines opens straightforward access for in situ generated nucleophilic carbenes derived from imidazole (imidazol-2-ylidenes) bearing an alkoxy group. This little-known class of alkoxy-substituted optically active NHCs is of interest for potential applications in asymmetric synthesis and in organometallic chemistry for the preparation of transition metal complexes. The multistep synthesis of the optically active imidazole-2-thiones occurs with preservation of the stereochemistry. In addition, many derivatives of chiral 1,2-diamines [13,15,31,32], imidazolium salts [33,34,35] as well as numerous imidazole-2-thiones [36,37] display diverse biological activities. Nevertheless, their alkoxy derivatives are practically unknown. For that reason, the new types of ‘desymmetrized’ derivatives of *trans*-1,2-diaminocyclohexane and other optically active primary amines reported in this work may also be of interest for medicinal chemistry.

## 4. Materials and Methods

### 4.1. General Information

Solvents and chemicals were purchased and used as received without further purification. Products were purified by standard column chromatography on silica gel (230–400 mesh, Merck, Kenilworth, NJ, USA). Unless stated otherwise, yields refer to analytically pure samples. NMR spectra were recorded with a Bruker Avance III 600 MHz instrument (^1^H-NMR: 600 MHz; ^13^C-NMR: 151 MHz; Bruker, Billerica, MA, USA). Chemical shifts are reported relative to solvent residual peaks (^1^H-NMR: δ = 7.26 ppm [CHCl_3_]; ^13^C-NMR: δ = 77.0 ppm [CDCl_3_]). IR spectra were recorded with a FTIR NEXUS spectrometer (as film or KBr pellets) or with a Cary 630 FTIR (Agilent Technologies, Santa Clara, CA, USA) spectrometer, in neat. ESI-MS spectra were performed with a Varian 500-MS LC Ion Trap. High-resolution mass spectrometry (HRMS) measurements were performed using Synapt G2-Si mass spectrometer (Waters, Milford, MA, USA) equipped with an ESI source and quadrupole-Time-of-flight mass analyzer. Elemental analyses were obtained with a Vario EL III (Elementar Analysensysteme GmbH, Langenselbold, Germany) instrument. Optical rotations were determined with an Anton Paar MCP 500 polarimeter (Anton Paar, Graz, Austria) at the temperatures indicated. Melting points were determined in capillaries with a Stuart SMP30 apparatus with automatic temperature monitoring or with a polarizing optical microscope (Opta-Tech, Warszawa, Poland), and are uncorrected.

### 4.2. Starting Materials

The ‘primary–tertiary’ amines **1** (i.e., compounds (*R*,*R*)-**1a**, (*R*,*R*)-**1b**, (*S*,*S*)-**1b,** (*R*,*R*)-**1c**, and (*R*,*R*)-**1d**) were prepared from the respective, enantiopure diastereomers of *trans*-1,2-diaminocyclohexane (*trans*-DACH), according to the literature procedure [12]. α-Hydroxyiminoketones **5** were prepared by nitrozation of ethyl methyl ketone in the case of **7a** [38] and by oximation of dibenzoyl in the case of **7b** [39] based on the published procedures. α-Methylbenzyloxyamine (α-MBOA) [27] and benzyloxyamine (BOA) [40] were prepared based on modified literature procedures. Formaldimines **4e** [20], **4f** [21], **4g** [21], and **4h** [22] were synthetized from corresponding amino compounds and formaldehyde based on the published procedures.

### 4.3. Synthesis of Formaldimines ***4***

#### 4.3.1. Reactions of ‘Primary-Tertiary’ Amines **1** with Formaldehyde—General Procedure

A solution of 5 mmol of the respective diamine **1** and 450 mg (15 mmol) of formaldehyde (used as an aqueous solution) in 50 mL of benzene was heated in the Dean-Stark apparatus over 1.5 h. Next, the solvent was evaporated and the crude products were obtained as colorless solids. Analytically pure samples were obtained by recrystallization.

(*R,R*)-*N*-[2-(Pyrrolidin-1-yl)cyclohexyl]methanimine ((*R*,*R*)-**4a**): Yield 855 mg (95%). Colorless crystals (diisopropyl ether). M.p. 97–98 °C. ${\left[\alpha \right]}_{D}^{25}$ = −135.30 (*c* 0.40, CHCl_3_). ^1^H-NMR (CDCl_3_): δ = 7.39 (d, *J* = 17.2 Hz, 1H), 7.13 (d, *J* = 17.2 Hz, 1H), 2.93–2.97 (m, 1H), 2.66–2.71 (m, 1H), 2.52–2.62 (m, 4H), 1.86–1.91 (m, 1H), 1.72–1.77 (m, 1H), 1.60–1.69 (m, 4H), 1.52–1.58 (m, 2H), 1.23–1.30 (m, 3H), 1.12–1.20 (m, 1H) ppm. ^13^C-NMR (CDCl_3_): δ = 151.1, 74.4, 63.4, 48.4, 34.0, 24.6, 24.2, 23.9, 23.5 ppm. IR: ν 2920 (vs), 2851 (s), 2801 (s), 1457 (m), 1384 (m), 1369 (m), 1183 (s), 1135 (vs), 997 (vs), 898 (m), 875 (m), 766 (m) cm^−1^. C_11_H_20_N_2_ (180.29): calcd. C 73.28, H 11.18, N 15.54; found C 73.27, H 11.15, N 15.60.

(*R*,*R*)-*N*-[2-(Piperidin-1-yl)cyclohexyl]methanimine ((*R*,*R*)-**4b**): Yield 756 mg (78%). Colorless crystals (diethyl ether). M.p. 126–128 °C. ${\left[\alpha \right]}_{D}^{25}$ = −130.92 (*c* 0.40, CHCl_3_). ^1^H-NMR (CDCl_3_): δ = 7.28 (d, *J* = 17.2 Hz, 1H), 7.13 (d, *J* = 17.2 Hz, 1H), 2.93 (td, *J* = 9.9, 5.2 Hz, 1H), 2.45–2.62 (m, 3H), 2.29–2.37 (m, 2H), 1.8–11.86 (m, 1H), 1.72–1.79 (m, 1H), 1.58–1.69 (m, 3H), 1.44–1.51 (m, 2H), 1.30–1.40 (m, 4H), 1.10–1.25 (m, 3H) ppm. ^13^C-NMR (CDCl_3_): δ = 151.0, 72.4, 67.3, 49.6, 34.6, 26.6, 25.6, 25.1, 24.6, 23.6 ppm. IR: ν 2924 (vs), 2849 (s), 1450 (m), 1381 (m), 1360 (m), 1191 (s), 1127 (vs), 1114 (vs), 1004 (vs), 911 (m), 876 (m), 766 (m), 641 (m) cm^–1^. C_12_H_22_N_2_ (194.32): calcd. C 74.17, H 11.41, N 14.42; found C 74.17, H 11.38, N 14.46.

(*S*,*S*)-*N*-[2-(Piperidin-1-yl)cyclohexyl]methanimine ((*S*,*S*)-**4b**): Yield 775 mg (80%). Colorless crystals (diethyl ether). M.p. 125–127 °C. ${\left[\alpha \right]}_{D}^{25}$ = +140.97 (*c* 0.40, CHCl_3_). ^1^H-NMR (CDCl_3_): δ = 7.28 (d, *J* = 17.2 Hz, 1H), 7.14 (d, *J* = 17.2 Hz, 1H), 2.94 (td, *J* = 9.9, 5.3 Hz, 1H), 2.46–2.61 (m, 3H), 2.28–2.36 (m, 2H), 1.82–1.86 (m, 1H), 1.73–1.79 (m, 1H), 1.59–1.70 (m, 3H), 1.44–1.52 (m, 2H), 1.31–1.40 (m, 4H), 1.12–1.26 (m, 3H) ppm. ^13^C-NMR (CDCl_3_): δ = 151.1, 72.4, 67.3, 49.6, 34.6, 26.6, 25.6, 25.1, 24.6, 23.6 ppm. IR: ν 2924 (vs), 2849 (s), 1450 (m), 1381 (m), 1360 (m), 1191 (s), 1127 (vs), 1114 (vs), 1004 (vs), 911 (m), 876 (m), 641 (m) cm^–1^. C_12_H_22_N_2_ (194.32): calcd. C 74.17, H 11.41, N 14.42; found C 73.99, H 11.55, N 14.38.

(*R*,*R*)-*N*-[2-(Azepan-1-yl)cyclohexyl]methanimine ((*R*,*R*)-**4c**): Yield 969 mg (93%). Colorless crystals (diisopropyl ether). M.p. 112–114 °C. ${\left[\alpha \right]}_{D}^{25}$ = –81.57 (*c* 0.40, CHCl_3_). ^1^H-NMR (CDCl_3_): δ = 7.32 (d, *J* = 17.3 Hz, 1H), 7.13 (d, *J* = 17.3 Hz, 1H), 2.90 (td, *J* = 10.0, 5.0 Hz, 1H), 2.64–2.68 (m, 2H), 2.59–2.64 (m, 1H), 2.50–2.55 (m, 2H), 1.79–1.82 (m, 1H), 1.71–1.76 (m, 1H), 1.63–1.68 (m, 1H), 1.41–1.62 (m, 10H), 1.15–1.24 (m, 3H) ppm. ^13^C-NMR (CDCl_3_): δ = 151.1, 73.7, 68.6, 51.4, 34.5, 30.0, 27.0, 25.6, 25.3, 24.6 ppm. IR: ν 2920 (vs), 2849 (s), 1448 (m), 1355 (m), 1166 (m), 1131 (s), 993 (m), 907 (m), 874 (m) cm^−1^. C_13_H_24_N_2_ (208.34): calcd. C 74.94, H 11.62, N 13.44; found C 75.10, H 11.73, N 13.61.

(*R,R*)-*N*-(2-Morpholinocyclohexyl)methanimine ((*R*,*R*)-**4d**): Yield 1.02 g (98%). Colorless crystals (diisopropyl ether). M.p. 127–129 °C. ${\left[\alpha \right]}_{D}^{25}$ = −124.52 (*c* 0.40, CHCl_3_). ^1^H-NMR (CDCl_3_): δ = 7.30 (d, *J* = 17.1 Hz, 1H), 7.14 (d, *J* = 17.1 Hz, 1H), 3.59–3.66 (m, 2H), 3.51–3.55 (m, 2H), 2.93 (td, *J* = 9.8, 5.5 Hz, 1H), 2.50–2.60 (m, 3H), 2.40–2.46 (m, 2H), 1.83–1.88 (m, 1H), 1.75–1.79 (m, 1H), 1.65–1.70 (m, 1H), 1.57–1.63 (m, 2H), 1.16–1.26 (m, 3H) ppm. ^13^C-NMR (CDCl_3_): δ = 151.4, 72.5, 67.6, 67.0, 48.9, 34.6, 25.5, 24.6, 23.9 ppm. IR: ν 2920 (s), 2851 (s), 1450 (m), 1258 (m), 1112 (vs), 995 (s), 872 (m) cm^–1^. C_11_H_20_N_2_O (196.29): calcd. C 67.31, H 10.27, N 14.27; found C 67.07, H 10.39, N 14.23.

#### 4.3.2. Synthesis of Alkoxyformaldimines **4i**–**j**—General Procedure

A solution of 3 mmol of the corresponding primary amine ((*S*)-MBOA or BOA) and 0.25 mL (ca. 3 mmol) of formaldehyde (as aqueous solution (37%)) in 40 mL benzene was heated in the Dean-Stark apparatus until no formation of water was observed. After cooling to room temperature, the solvent was evaporated and the colorless, oily liquids were used for further reactions without purification.

(*S*)-(α-Methylbenzyloxy)methanimine (**4i**): Yield 404 mg (90%). Colorless liquid. ${\left[\alpha \right]}_{D}^{25}$ = −11.90 (*c* 0.51, CHCl_3_). ^1^H-NMR (CDCl_3_): δ = 7.38–7.33, 7.30–7.27 (2m, 4H, 1H, Ph), 7.08, 6.43 (2d, 2H, *J* = 8.5 Hz, =CH_2_), 5.27 (q, *J* = 6.7 Hz, 1H), 1.56 (d, *J* = 6.7 Hz, 3H, Me) ppm. ^13^C-NMR (CDCl_3_): δ = 143.0 (t, N=CH_2_), 137.4, 128.4, 127.6, 126.3 (s, 3d, Ph), 81.1 (d, CH), 21.8 (q, Me) ppm. HRMS (CI^+^): calcd for C_9_H_13_NO [M + H]^+^ 150.0917; found 150.0919.

(Benzyloxy)methanimine (**4j**): Yield 360 mg (88%). Colorless liquid (ref. [41], bp 76 °C/15 mmHg). ^1^H-NMR (CDCl_3_): δ = 7.40–7.35, 7.34–7.30 (2m, 4H, 1H, Ph), 7.09, 6.47 (2d, 2H, *J* = 8.2 Hz, =CH_2_), 5.14 (s, 2H, -C*H*_2_-) ppm. ^13^C-NMR (CDCl_3_): δ = 137.4 (t, N=*C*H_2_), 137.3 (s, Ph), 128.3, 128.1, 127.8 (3d, Ph), 75.8 (t, -CH_2_-) ppm.

### 4.4. Synthesis of Imidazole N-Oxides ***6***—General Procedure

A solution of 1.1 mmol of the respective formaldimine **4** and 1 mmol of α-hydroxyiminoketone **5** in 3 mL of glacial acetic acid was stirred magnetically overnight. The next day, 1 mL of aqueous hydrochloric acid was added and the solution was stirred for 15 min. Then, the resulting solution was evaporated to dryness, the solid residue was dissolved in 20 mL of dichloromethane, and the obtained solution was neutralized with 20 mL of diluted aqueous sodium hydroxide. The organic layer was separated, dried over MgSO_4_, filtrated, and evaporated. The resulting crude products were washed with two portions (each ca. 10 mL) of diethyl ether, and after separation, the obtained thick oils or amorphous solids were characterized spectroscopically and used for further transformations.

(*R,R*)-4,5-Dimethyl-1-[2-(pyrrolidin-1-yl)cyclohexyl]-1*H*-imidazole 3-oxide ((*R*,*R*)-**6a**): Yield 171.0 mg (65%). Colorless, viscous oil. ^1^H-NMR (CDCl_3_): δ = 7.88 (s, 1H), 3.73 (td, *J* = 11.2, 3.5 Hz, 1H), 2.81–2.87 (m, 1H), 2.45–2.49 (m, 2H), 2.37–2.41 (m, 2H), 2.14 (s, 3H), 2.10 (s, 3H), 2.00–2.03 (m, 1H), 1.93–1.97 (m, 1H), 1.80–1.84 (m, 1H), 1.76–1.79 (m, 1H), 1.44–1.60 (m, 4H), 1.14–1.35 (m, 4H) ppm. ^13^C-NMR (CDCl_3_): δ = 125.2, 123.0, 120.6, 62.1, 58.7, 48.0, 35.0, 25.2, 24.8, 24.6, 23.6, 8.8, 7.1 ppm. IR: ν 2927 (s), 1655 (m), 1448 (m), 1377 (m), 1332 (vs), 1191 (m), 1142 (m), 883 (s), 702 (s), 579 (s) cm^−1^. HRMS (ESI^+^): calcd for C_15_H_26_N_3_O [M + H]^+^ 264.2076; found 264.2082.

(*R*,*R*)-4,5-Diphenyl-1-[2-(pyrrolidin-1-yl)cyclohexyl]-1*H*-imidazole 3-oxide ((*R*,*R*)-**6b**): Yield 360.0 mg (93%). Yellowish crystals. M.p. 189–191 °C. ^1^H-NMR (CDCl_3_): δ = 8.12 (s, 1H), 7.49 (d, *J* = 7.9 Hz), 7.35–7.39 (m, 3H), 7.18–7.23 (m, 5H), 3.74 (td, *J* = 11.7, 4.2 Hz, 1H), 2.86 (td, *J* = 11.1, 3.5 Hz, 1H), 2.32–2.36 (m, 2H), 2.25–2.29 (m, 2H), 2.06–2.11 (m, 1H), 1.89–1.94 (m, 1H), 1.47–1.78 (m, 8H), 1.21–1.29 (m, 1H), 1.08–1.18 (m, 3H) ppm. ^13^C-NMR (CDCl_3_): δ = 130.7, 129.6, 129.3, 129.1, 128.9, 128.0, 127.9, 127.8, 127.3, 127.0, 124.2, 62.3, 58.7, 47.6, 35.4, 25.2, 24.6, 24.0, 23.56, 23.51 ppm. IR: ν 2931 (m), 2808 (s), 1448 (m), 1341 (m), 1321 (m), 1224 (m), 881 (m), 754 (s), 710 (s), 689 (vs), 658 cm^−1^. C_25_H_29_N_3_O·0.5 H_2_O (387.52 + 9): calcd. C 75.72, H 7.62, N 10.60; found C 75.50, H 7.59, N 10.61.

(*R*,*R*)-4,5-Dimethyl-1-[2-(piperidin-1-yl)cyclohexyl]-1*H*-imidazole 3-oxide ((*R*,*R*)-**6c**): Yield 249.5 mg (90%). Yellowish crystals. M.p. 68–71 °C. ^1^H-NMR (CDCl_3_): δ = 7.70 (s, 1H), 3.75 (td, *J* = 11.5, 3.8 Hz, 1H), 2.47–2.54 (m, 3H), 2.23–2.27 (m, 2H), 2.19 (s, 3H), 2.11 (s, 3H), 1.99–2.02 (m, 2H), 1.78–1.87 (m, 2H), 1.45–1.52 (m, 1H), 1.37–1.42 (m, 2H), 1.24–1.33 (m, 7H) ppm. ^13^C-NMR (CDCl_3_): δ = 124.9, 123.4, 120.8, 67.7, 56.6, 50.0, 34.9, 26.5, 25.4, 25.0, 24.7 (2C signals overlap), 8.9, 7.2 ppm. IR: ν 2926 (vs), 2853 (m), 1655 (br.m), 1450 (s), 1377 (m), 1331 (vs), 1103 (m), 881 (m), 702 (s), 579 (s) cm^–1^. HRMS (ESI^+^): calcd for C_16_H_28_N_3_O [M + H]^+^ 278.2232; found 278.2234.

(*S*,*S*)-4,5-Dimethyl-1-[2-(piperidin-1-yl)cyclohexyl]-1*H*-imidazole 3-oxide ((*S*,*S*)-**6c**): Yield: 263.5 g (95%). Yellowish crystals. M.p. 51–54 °C. ^1^H-NMR (CDCl_3_): δ = 7.87 (s, 1H), 3.70 (td, *J* = 11.5, 3.8 Hz, 1H), 2.51–2.56 (m, 1H), 2.41–2.46 (m, 2H), 2.27–2.21 (m, 2H), 2.12 (s, 3H), 2.06 (s, 3H), 1.92–1.96 (m, 2H), 1.71–1.80 (m, 2H), 1.46–1.53 (m, 1H), 1.29–1.35 (m, 2H), 1.18–1.25 (m, 7H) ppm. ^13^C-NMR (CDCl_3_): δ = 124.7, 122.8, 120.6, 67.6, 56.3, 49.9, 34.8, 26.3, 25.2, 24.9, 24.51, 24.46, 8.7, 7.1 ppm. IR: ν 2928 (vs), 2855 (m), 1664 (br, m), 1450 (s), 1379 (s), 1332 (vs), 1101 (m), 881 (m), 704 (s), 581 (s) cm^−1^. HRMS (ESI^+^): calcd for C_16_H_28_N_3_O [M + H]^+^ 278.2232; found 278.2238.

(*R*,*R*)-4,5-Diphenyl-1-[2-(piperidin-1-yl)cyclohexyl]-1*H*-imidazole 3-oxide ((*R*,*R*)-**6d**): Yield 261.0 mg (65%). Yellowish crystals. M.p. 192–194 °C. ^1^H-NMR (CDCl_3_): δ = 8.00 (s, 1H), 7.57 (d, *J* = 7.5 Hz, 2H), 7.39–7.45 (m, 3H), 7.23–7.29 (m, 5H), 3.82 (td, *J* = 11.5, 3.8 Hz, 1H), 2.61 (td, *J* = 11.3, 3.3 Hz, 1H), 2.25–2.29 (m, 2H), 2.17–2.22 (m, 2H), 1.93–1.97 (m, 1H), 1.78–1.83 (m, 2H), 1.63–1.70 (m, 1H), 1.00–1.44 (m, 10H) ppm. ^13^C-NMR (CDCl_3_): δ = 130.6, 129.6, 129.2, 128.9, 128.2, 127.9, 127.7, 127.36, 127.28, 124.1, 68.2, 56.7, 49.7, 35.7, 26.5, 25.3, 25.0, 24.7, 24.0 ppm. IR: ν 2929 (s), 2855 (m), 1444 (m), 1342 (m), 1205 (w), 1101 (w), 1034 (w), 784 (s), 710 (s), 691 (s) cm^−1^. C_26_H_31_N_3_O (401.54): calcd. C 77.77, H 7.78, N 10.46; found C 77.56, H 7.88, N 10.59.

(*S*,*S*)-4,5-Diphenyl-1-[2-(piperidin-1-yl)cyclohexyl]-1*H*-imidazole 3-oxide ((*S*,*S*)-**6d**): Yield: 333.0 mg (83%) after purification on a short column. Yellowish crystals. M.p. 198–199 °C. ^1^H-NMR (CDCl_3_): δ = 8.03 (s, 1H), 7.56 (d, *J* = 7.5 Hz, 2H), 7.40–7.45 (m, 3H), 7.23–7.29 (m, 5H), 3.81 (td, *J* = 11.6, 3.8 Hz, 1H), 2.61 (td, *J* = 11.3, 3.4 Hz, 1H), 2.24–2.28 (m, 2H), 2.17–2.22 (m, 2H), 1.92–1.97 (m, 1H), 1.78–1.83 (m, 2H), 1.68 (qd, *J* = 12.5, 3.3 Hz, 1H), 1.06–1.44 (m, 10H) ppm. ^13^C-NMR (CDCl_3_): δ = 130.5, 129.6, 129.3, 128.95, 128.89, 127.95, 127.91, 127.79, 127.4, 127.1, 68.0, 56.9, 49.6, 35.4, 26.5, 25.3, 24.9, 24.6, 24.0 ppm. IR: ν 2928 (s), 2853 (m), 1444 (m), 1342 (m), 1205 (m), 1103 (m), 1034 (w), 786 (s), 712 (s), 693 (vs) cm^−1^. HRMS (ESI^+^): calcd for C_26_H_32_N_3_O [M + H]^+^ 402.2545; found 402.2546.

(*R*,*R*)-1-[2-(Azepan-1-yl)cyclohexyl]-4,5-dimethyl-1*H*-imidazole 3-oxide ((*R*,*R*)-**6e**): Yield 227.1 g (78 %). Colorless, viscous oil. ^1^H-NMR (CDCl_3_): δ = 7.74 (s, 1H), 3.66 (td, *J* = 11.2, 3.9 Hz, 1H), 2.63–2.67 (m, 1H), 2.53–2.58 (m, 2H), 2.37–2.42 (m, 2H), 2.11 (s, 3H), 2.06 (s, 3H), 1.91–1.96 (m, 2H), 1.71–1.79 (m, 2H), 1.15–1.53 (m, 12H) ppm. ^13^C-NMR (CDCl_3_): δ = 124.7, 123.4, 120.1, 67.2, 57.3, 50.4, 35.0, 29.5, 26.5, 25.2, 25.0, 24.7, 8.6, 6.9 ppm. IR: ν 2924 (vs), 2853 (m), 1654 (s), 1448 (s), 1332 (s), 725 (vs), 704 (vs), 581 (m) cm^–1^. HRMS (ESI^+^): calcd for C_17_H_30_N_3_O [M + H]^+^ 292.2389; found 292.2398.

(*R*,*R*)-1-[2-(Azepan-1-yl)cyclohexyl]-4,5-diphenyl-1*H*-imidazole 3-oxide ((*R*,*R*)-**6f**): Yield: 369.6 mg (89%). Yellowish crystals. M.p. 64–68 °C. ^1^H-NMR (CDCl_3_): δ = 8.06 (s, 1H), 7.50 (d, *J* = 7.7 Hz, 2H), 7.37–7.41 (m, 3H), 7.19–7.24 (m, 5H), 3.75 (td, *J* = 11.4, 3.7 Hz, 1H), 2.71 (td, *J* = 11.3, 3.5 Hz, 1H), 2.35–2.44 (m, 4H), 2.13–2.17 (m, 1H), 1.88–1.93 (m, 1H), 1.72–1.78 (m, 3H), 1.09–1.64 (m, 11H) ppm. ^13^C-NMR (CDCl_3_): δ = 130.5, 129.7, 129.4, 129.0, 128.00, 127.96, 127.5, 127.0, 126.7, 125.9, 125.5, 68.3, 57.9, 50.5, 35.7, 29.7, 26.7, 25.2, 24.9 ppm (one signal of sp^3^ C atom not observed due to overlap). IR: ν 2926 (s), 2855 (w), 1672 (m), 1444 (m), 1224 (w), 712 (s), 693 (br, s) 635 (m), 508 (br, m), 451 (br, m) cm^−1^. HRMS (ESI^+^): calcd for C_17_H_34_N_3_O [M + H]^+^ 416.2702; found 416.2708.

(*R*,*R*)-1-(2-Morpholinocyclohexyl)-4,5-dimethyl-1*H*-imidazole 3-oxide ((*R*,*R*)-**6g**): Yield: 240.1 mg (86%). Yellowish crystals. M.p. 85–87 °C. ^1^H-NMR (CDCl_3_): δ = 9.06 (s, 1H), 3.76–3.82 (m, 1H), 3.58–3.66 (m, 1H), 3.43–3.48 (m, 2H), 3.36–3.40 (m, 2H), 3.10 (td, *J* = 11.1, 3.1 Hz, 1H), 2.49–2.54 (m, 2H), 2.39–2.43 (m, 2H), 2.15 (s, 3H), 2.09 (s, 3H), 1.95–2.04 (m, 2H), 1.86–1.93 (m, 1H), 1.76–1.82 (m, 2H), 1.42–1.50 (m, 1H), 1.20–1.28 (m, 2H) ppm. ^13^C-NMR (CDCl_3_): δ = 126.2, 124.4, 121.0, 67.3, 66.0, 57.2, 48.8, 34.2, 25.3, 24.9, 24.5, 8.9, 7.1 ppm. IR ν 2929 (s), 2855 (m), 1654 (w), 1627 (br, w), 1450 (m), 1332 (s), 1112 (vs), 926 (m), 861 (m), 725 (vs) cm^−1^. HRMS (ESI^+^): calcd for C_15_H_26_N_3_O_2_ [M + H]^+^ 280.2025; found 280.2026.

(*R*,*R*)-1-(2-Morpholinocyclohexyl)-4,5-diphenyl-1*H*-imidazole 3-oxide ((*R*,*R*)-**6h**): Yield: 266.3 mg (66%). Yellowish crystals. M.p. 152–155 °C. ^1^H-NMR (CDCl_3_): δ = 8.08 (s, 1H), 7.54 (d, *J* = 7.2 Hz, 2H), 7.38–7.43 (m, 3H), 7.24–7.29 (m, 3H), 7.19 (d, *J* = 6.9 Hz, 2H), 3.84 (td, *J* = 11.6, 4.0 Hz, 1H), 3.44–3.51 (m, 4H), 2.62 (td, *J* = 11.3, 3.1 Hz, 1H), 2.22–2.28 (m, 5H), 1.94–1.98 (m, 1H), 1.81–1.86 (m, 2H), 1.69–1.76 (m, 1H), 1.25–1.33 (m, 1H), 1.16–1.24 (m, 1H), 1.08–1.15 (m, 1H) ppm. ^13^C-NMR (CDCl_3_): δ = 130.4, 129.7, 129.3, 129.0, 127.99, 127.92, 127.88, 127.4, 127.1, 123.9, 68.1, 67.3, 56.3, 48.6, 35.2, 25.2, 24.8, 24.2 ppm (one signal of an sp^2^ C atom not observed due to overlap). IR: ν 2926 (s), 2857 (m), 1448 (m), 1341 (m), 1109 (s), 861 (s), 766 (s), 695 (vs), 665 (s), 633 (s) cm^−1^. C_25_H_29_N_3_O_2_·0.5 H_2_O (412.52): calcd. C 72.79, H 7.33, N 10.18; found C 72.68, H 7.25, N 9.90.

### 4.5. Synthesis of 3-Benzyloxyimidazolium Bromides ***7***—General Procedure

A solution of 0.5 mmol of crude imidazole *N*-oxide **6** and 86 mg (0.5 mmol) benzyl bromide in 1 mL of CHCl_3_ was stirred magnetically overnight at room temperature. The next day, the solvent was evaporated and the obtained crude imidazolium salts were triturated with diethyl ether. The ethereal phase was separated and the non-soluble imidazolium salts **7** were used for the generation of carbenes **8** and their reaction with elemental sulfur without further purification. Whereas di-Me substituted imidazolium salts formed viscous, thick oils, the corresponding di-Ph derivatives were obtained as amorphous solids. The structures of selected imidazolium salts were confirmed by running the ^1^H-NMR spectra. A representative example of the ^1^H-NMR spectra registered for **7e** is described below and the scanned spectrum is presented in the Supplementary Materials.

(*R*,*R*)-1-[2-(Azepan-1-yl)cyclohexyl]-4,5-dimethyl-3-benzyloxy-1*H*-imidazolium bromide((*R*,*R*)-**7e**): ^1^H-NMR (CDCl_3_): δ = 11.09 (s, 1H), 7.51 (d, *J* = 6.9 Hz, 2H), 7.27–7.37 (m, 3H), 5.70 (d, *J*= 10.0 Hz, 1H), 5.57 (d, *J*= 10.0 Hz, 1H), 3.91 (br., 1H), 3.62 (br., 1H), 2.60 (br., 4H), 2.13–2.17 (m, 1H), 2.13 (s, 3H), 2.02–2.06 (m, 1H), 1.96–2.01 (m, 1H), 1.91 (s, 3H), 1.80–1.85 (m, 1H), 1.74–1.79 (m, 1H), 1.55–1.63 (m, 1H), 1.47 (br., 2H), 1.29 (br., 6H), 1.18 (br., 2H) ppm.

### 4.6. Synthesis of Non-Symmetric Imidazole-2-Thiones ***9***—General Procedure

The crude imidazolium salt **7** obtained from 0.5 mmol of the corresponding imidazole *N*-oxide **6** and 86 mg (0.5 mmol) of benzyl bromide according to the general procedure (see above) was dissolved in 1 mL of dry pyridine. Next, 38 mg (1.2 mmol) of elemental sulfur and 122 mg (1.2 mmol) of triethylamine were added to the magnetically stirred, homogenous solution. Stirring at room temperature was continued overnight. Next day, pyridine was removed under reduced pressure and the residual semi-solid material was purified by preparative thin layer chromatography with silica gel. A mixture of dichloromethane and methanol (99:1) was used as an eluent. Imidazole-2-thiones **9** formed a single fraction with *R*_f_ ca. 0.3. Solid products were additionally purified by crystallization.

(*R,R*)-3-Benzyloxy-4,5-dimethyl-1-[2-(pyrrolidin-1-yl)cyclohexyl]-1,3-dihydro-2*H*-imidazole-2-thione ((*R,R*)*-***9a**): Yield: 238.8 mg (62%). Colorless, viscous oil, purified by chromatography. ${\left[\alpha \right]}_{D}^{25}$ = −91.15 (*c* 0.40, CHCl_3_). ^1^H-NMR (CDCl_3_): δ = 7.32–7.44 (m, 5H), 5.75–5.80 (m, 1H), 5.42 (d, *J* = 10.0 Hz, 1H), 5.28 (d, *J* = 10.0 Hz, 1H), 4.57–4.63 (m, 1H), 3.06–3.11 (m, 1H), 2.83–3.02 (m, 3H), 2.36 (s, 3H), 2.26–2.31 (m, 1H), 1.72–2.13 (m, 8H), 1.81 (s, 3H), 1.38–1.51 (m, 3H) ppm. ^13^C-NMR (CDCl_3_): δ = 155.1, 133.3, 130.2, 129.5, 128.5, 121.1, 118.4, 78.1, 60.7, 58.4, 52.2, 29.6, 29.4, 24.1, 23.7, 22.6, 9.9, 7.7 ppm. IR ν 2929 (s), 2862 (m), 1377 (br, s), 1401 (br, s), 1321 (vs), 1140 (br, m), 1025 (m),954 (m), 913 (m), 836 (w), 751 (s), 697 (vs), 606 (w), 483 (s) cm^−1^. HRMS (ESI^+^): calcd for C_22_H_32_N_3_OS [M + H]^+^ 386.2266; found 386.2274.

(*R*,*R*)-3-Benzyloxy-4,5-diphenyl-1-[2-(pyrrolidin-1-yl)cyclohexyl]-1,3-dihydro-2*H*-imidazole-2-thione ((*R*,*R*)-**9b**): Yield: 356.8 g (70%). Colorless crystals. M.p. 285–286 °C (from MeOH/CH_2_Cl_2_) (decomp.). ${\left[\alpha \right]}_{D}^{25}$ = −36.65 (*c* 0.40, CHCl_3_). ^1^H-NMR (CDCl_3_): δ = (signals for both rotamers) 6.96–7.45 (m, 15H), 4.95–5.47 (m, 3H), 3.44 and 3.74 (br., 1H), 2.14–2.70 (m, 4H), 0.80–1.96 (m, 12H) ppm. ^13^C-NMR (CDCl_3_): δ = (signals for both rotamers) 157.3, 156.1, 133.2, 132.6, 131.1, 130.6, 129.4, 129.3, 129.0, 128.7, 128.2, 128.0, 127.8, 126.4, 125.2, 124.5, 77.4, 61.7, 58.2, 55.0, 46.4, 33.3, 29.4, 26.0, 25.8, 25.0, 24.8, 23.8, 23.6, 22.1 ppm. IR: ν 2929 (m), 2797 (m), 1425 (m), 1358 (m), 1332 (m), 1306 (br, m), 1187 (w), 1073 (w), 965 (m), 911 (m), 786 (m), 753 (s), 695 (vs), 598 (m), 506 (m) cm^−1^. HRMS (ESI^+^): calcd. for C_32_H_36_N_3_OS [M + H]^+^ 510.2579; found 510.2595. C_32_H_35_N_3_OS (509.70): calcd. C 75.40, H 6.92, N 8.24, S 6.30; found C 75.30, H 6.91, N 8.24, S 6.14.

(*R*,*R*)-3-Benzyloxy-4,5-dimethyl-1-[2-(piperidin-1-yl)cyclohexyl]-1,3-dihydro-2*H*-imidazole-2-thione ((*R*,*R*)-**9c**): Yield: 239.5 mg (60%). Colorless, viscous oil, purified by chromatography. ${\left[\alpha \right]}_{D}^{25}$ = −16.53 (*c* 0.40, CHCl_3_). ^1^H-NMR (CDCl_3_): δ = 7.47 (d, *J* = 7.7 Hz, 2H), 7.32–7.37 (m, 3H), 5.46 (d, *J* = 10.5 Hz, 1H), 5.32 (d, *J* = 10.5 Hz, 1H), 5.27 (td, *J* = 11.6, 3.7 Hz, 1H), 2.89 (td, *J* = 11.4, 2.8 Hz, 1H), 2.74–2.79 (m, 2H), 2.23–2.27 (m, 2H), 2.08 (s, 3H), 1.94–2.02 (m, 2H), 1.83–1.88 (m, 1H), 1.73–1.78 (m, 1H), 1.70 (s, 3H), 1.59 (qd, *J* = 12.5, 3.7 Hz, 1H), 1.29–1.52 (m, 8H), 1.17–1.25 (m, 1H) ppm. ^13^C-NMR (CDCl_3_): δ = 156.5, 134.2, 130.5, 129.1, 128.4, 119.4, 117.9, 77.6, 65.9, 58.5, 49.4, 32.0, 26.8, 25.9, 25.7, 24.9, 24.6, 10.5, 7.2 ppm. IR: ν 2927 (vs), 2853 (m), 1403 (s), 1377 (s), 1330 (s), 1211 (m), 1105 (m), 963 (m), 911 (m), 749 (vs), 699 (vs), 479 (br.s) cm^−1^. HRMS (ESI^+^) calcd. for C_23_H_34_N_3_OS [M + H]^+^ 400.2423; found: 400.2424.

(*S*,*S*)-3-Benzyloxy-4,5-dimethyl-1-[2-(piperidin-1-yl)cyclohexyl]-1,3-dihydro-2*H*-imidazole-2-thione ((*S*,*S*)-**9c**): Yield: 187.6 mg (47%). Colorless, viscous oil, purified by chromatography. ${\left[\alpha \right]}_{D}^{25}$ = +26.13 (*c* 0.40, CHCl_3_). ^1^H-NMR (CDCl_3_): δ = 7.46 (d, *J* = 7.5 Hz, 2H), 7.32–7.37 (m, 3H), 5.46 (d, *J* = 10.5 Hz, 1H), 5.31 (d, *J* = 10.5 Hz, 1H), 5.26 (td, *J* = 11.9, 4.2 Hz, 1H), 2.89 (td, *J* = 11.4, 2.8 Hz, 1H), 2.74–2.78 (m, 2H), 2.23–2.27 (m, 2H), 2.07 (s, 3H), 1.94–2.03 (m, 2H), 1.83–1.87 (m, 1H), 1.73–1.77 (m, 1H), 1.68 (s, 3H), 1.58 (qd, *J* = 12.5, 3.7 Hz, 1H), 1.27–1.51 (m, 8H), 1.17–1.25 (m, 1H) ppm. ^13^C-NMR (CDCl_3_): δ = 156.3, 134.2, 130.5, 129.1, 128.4, 119.4, 117.9, 77.6, 65.9, 58.5, 49.4, 32.0, 26.8, 25.8, 25.7, 24.9, 24.5, 10.5, 7.2 ppm. IR: ν 2927 (vs), 2853 (m), 1403 (s), 1377 (s), 1328 (s), 1211 (m), 1105 (m), 963 (m), 911 (w), 749 (vs), 697 (vs), 479 (br, s) cm^−1^. HRMS (ESI^+^): calcd for C_23_H_34_N_3_OS [M + H]^+^ 400.2423; found 400.2426.

(*R*,*R*)-3-Benzyloxy-4,5-diphenyl-1-[2-(piperidin-1-yl)cyclohexyl]-1,3-dihydro-2*H*-imidazole-2-thione ((*R*,*R*)-**9d**): Yield 429.4 mg (82%). Colorless crystals. M.p. 283–285 °C (MeOH/CH_2_Cl_2_) (decomp.). ${\left[\alpha \right]}_{D}^{25}$ = −26.77 (*c* 0.40, CHCl_3_). ^1^H-NMR (CDCl_3_): δ = (signals for both rotamers in ca. 1:2 ratio) 7.07–7.41 (m, 15H), 5.40 and 5.32 (d, *J* = 9.4 Hz, 1H), 4.61–4.67 (m) and 5.36 (td, *J* = 11.8, 3.4 Hz, total: 1H), 4.98 and 5.12 (br. d, *J* = 9.4 Hz, 1H), 0.77–3.76 (m, 18H) ppm. ^13^C-NMR (CDCl_3_): δ = (signals for both rotamers) 157.6, 156.7, 133.2, 133.1, 132.4, 130.6, 130.42, 130.38, 129.5, 129.3, 129.22, 129.15, 128.92, 128.87, 128.84, 128.6, 128.4, 128.3, 128.1, 128.02, 127.97, 127.8, 127.7, 126.9, 126.6, 126.40, 126.31, 124.9, 124.6, 124.3, 77.51, 77.49, 64.4, 59.9, 59.7, 49.1 (br.), 48.8 (br.), 33.2, 29.6, 27.1, 26.8, 26.0, 25.7, 25.2, 24.94, 24.91, 24.6, 23.5 ppm. IR: ν 2924 (s), 2849 (w), 1442 (w), 1397 (s), 1321 (s), 1207 (m), 959 (m), 760 (s), 691 (vs), 596 (m), 568 (m), 475 (w) cm^−1^. C_33_H_37_N_3_OS (523.73): calcd. C 75.68, H 7.13, N 8.02, S 6.12; found C 75.63, H 7.17, N 8.00. S 6.00.

(*S*,*S*)-3-Benzyloxy-4,5-diphenyl-1-[2-(piperidin-1-yl)cyclohexyl]-1,3-dihydro-2*H*-imidazole-2-thione ((*S*,*S*)-**9d**): Yield 324.7 mg (62%). Viscous oil (after chromatography). ${\left[\alpha \right]}_{D}^{25}$ = +22.42 (*c* 0.40, CHCl_3_). ^1^H-NMR (CDCl_3_): δ = (signals for both rotamers in ca. 1:2 ratio) 7.07–7.41 (m, 15H), 5.41 and 5.32 (d, *J* = 9.3 Hz, 1H), 4.61–4.67 and 5.33–5.38 (m, 1H), 4.98 and 5.12 (br. d, *J* = 9.3 Hz, 1H), 0.78–3.74 (m, 18H) ppm. ^13^C-NMR (CDCl_3_): δ = (signals for both rotamers) 157.6, 156.7, 133.14, 133.06, 132.4, 130.6, 130.43, 130.40, 129.5, 129.4, 129.2, 128.93, 128.89, 128.88, 128.7, 128.5, 128.13, 128.05, 127.8, 127.7, 126.9, 126.7, 126.4, 125.9, 124.9, 124.6, 124.4, 77.53, 77.51, 64.4, 60.0, 59.7, 49.2 (br.), 33.2, 29.6, 27.1, 26.8, 26.0, 25.7, 25.2, 24.9, 24.6, 23.5 ppm. IR: ν 2927 (s), 2853 (m), 1444 (w), 1396 (w), 1321 (m), 1207 (m), 959 (m), 753 (s), 693 (vs), 596 (m), 568 (m), 475 (w) cm^−1^. HRMS (ESI^+^): calcd for C_33_H_38_N_3_OS [M + H]^+^ 524.2736; found 524.2743. C_33_H_37_N_3_OS (523.73): calcd. C 75.68, H 7.13, N 8.02, S 6.12; found C 75.64, H 7.10, N 8.06, S 5.92.

(*R*,*R*)-3-Benzyloxy-1-[2-(azepan-1-yl)cyclohexyl]-4,5-dimethyl-1,3-dihydro-2*H*-imidazole-2-thione ((*R*,*R*)-**9e**): Yield: 173.7 mg (42 %).Viscous oil. ${\left[\alpha \right]}_{D}^{25}$ = −28.85 (*c* 0.40, CHCl_3_). ^1^H-NMR (CDCl_3_): δ = 7.46–7.48 (m, 2H), 7.33–7.36 (m, 3H), 5.38 (d, *J* = 10.4 Hz, 1H), 5.32 (d, *J* = 10.4 Hz, 1H), 5.21 (td, *J* = 11.6, 3.4 Hz, 1H), 2.97 (td, *J* = 11.6, 2.7 Hz, 1H), 2.79–2.84 (m, 2H), 2.40–2.44 (m, 2H), 2.11 (s, 3H), 1.93–2.01 (m, 2H), 1.82–1.86 (m, 1H), 1.73–1.77 (m, 1H), 1.74 (s, 3H), 1.57 (qd, *J* = 12.4, 3.3 Hz, 1H), 1.31–1.52 (m, 10H) 1.16–1.25 (m, 1H) ppm. ^13^C-NMR (CDCl_3_): δ = 156.6, 134.2, 130.3, 129.1, 128.4, 119.2, 118.1, 77.9, 67.1, 59.4, 51.2, 32.1, 30.0, 26.6, 25.8, 25.7, 25.5, 10.6, 7.2 ppm. IR: ν 2922 (vs), 2853 (m), 1403 (s), 1377 (s), 1328 (s), 1170 (w), 1133 (w), 959 (m), 907 (m), 749 (vs), 697 (vs) cm^−1^. C_24_H_35_N_3_OS (413.62): calcd. C 69.69, H 8.53, N 10.16., S 7.75; found C 69.47, H 8.62, N 10.21, S 7.59.

(*R*,*R*)-3-Benzyloxy-1-[2-(azepan-1-yl)cyclohexyl]-4,5-diphenyl-1,3-dihydro-2*H*-imidazole-2-thione ((*R*,*R*)-**9f**): Yield: 376.4 mg (70%). Colorless crystals. M.p. 264–267 °C (MeOH/CH_2_Cl_2_) (decomp.). ${\left[\alpha \right]}_{D}^{25}$ = −27.17 (*c* 0.40, CHCl_3_). ^1^H-NMR (CDCl_3_): δ = (signals for both rotamers in ca. 1:2 ratio) 7.05–7.38 (m, 15H), 5.32 and 4.72 (td, *J* = 11.5, 3.6 Hz, 1H), 5.31 and 5.21 (d, *J* = 9.3 Hz, 1H), 5.02 (br.) and 5.15 (d, *J* = 9.3 Hz, 1H), 3.69 (td, *J* = 11.7, 3.0 Hz, 0.3H), 3.28–3.36 (m, 0.3H), 2.75–2.80 (m, 1H), 2.43–2.47 (m, 0.7H), 2.29–2.32 (m, 0.7H), 1.34–2.21 (m, 15H), 1.16 and 0.98 (qd, *J* = 12.8, 3.5 Hz, 1H), 1.03–1.11 and 0.73–0.82 (m, 1H) ppm. ^13^C-NMR (CDCl_3_): δ = 158.0, 157.0, 133.2, 133.1, 132.5, 130.6, 130.37, 130.3, 129.6, 129.4, 129.2, 129.1, 128.9, 128.6, 128.15, 128.13, 128.10, 128.07, 127.9, 127.8, 127.7, 126.6, 126.4, 126.0, 125.0, 124.7, 77.62, 77.58, 66.1, 61.4, 60.5, 51.4 (br.), 50.5, 33.3, 30.05, 29.97, 29.8, 26.9, 26.6, 26.4, 26.0, 25.8, 25.23, 25.17 ppm. IR: ν 2924 (s), 2853 (m), 1446 (m), 1356 (m), 1332 (m), 1073 (w), 963 (w), 752 (s), 693 (vs) cm^−1^. C_34_H_39_N_3_OS (537.76): calcd. C 75.94, H 7.31, N 7.81, S 5.96; found C 75.91, H 7.45, N 8.01, S 5.86.

(*R*,*R*)-3-Benzyloxy-1-(2-morpholinocyclohexyl)-4,5-dimethyl-1,3-dihydro-2*H*-imidazole-2-thione ((*R*,*R*)-**9g**): Yield 135 mg (67%). Colorless crystals. M.p. 122–124 °C (after chromatographic separation). ${\left[\alpha \right]}_{D}^{25}$ = −21.22 (*c* 0.40, CHCl_3_). ^1^H-NMR (CDCl_3_): δ = 7.43–7.46 (m, 2H), 7.33–7.37 (m, 3H), 5.44 (d, *J* = 10.4 Hz, 1H), 5.30 (d, *J* = 10.4 Hz, 1H), 5.27 (td, *J* = 11.9, 3.7 Hz, 1H), 3.52–3.57 (m, 2H), 3.45–3.50 (m, 2H), 2.92 (dt, *J* = 11.4, 3.2 Hz, 1H), 2.85–2.90 (m, 2H), 2.30–2.34 (m, 2H), 2.08 (s, 3H), 1.97–2.02 (m, 2H), 1.85–1.90 (m, 1H), 1.75–1.80 (m, 1H), 1.72 (s, 3H), 1.62 (qd, *J* = 12.4, 3.8 Hz, 1H), 1.42–1.51 (m, 1H), 1.32–1.40 (m, 1H), 1.17–1.27 (m, 1H) ppm. ^13^C-NMR (CDCl_3_): δ = 156.5, 134.1, 130.5, 129.3, 128.6, 119.9, 117.7, 77.8, 67.7, 65.4, 58.1, 48.6, 31.9, 25.8, 25.5, 24.5, 10.7, 7.3 ppm. IR: ν 2935 (br, m), 2851 (m) 2816 (w), 1451 (m), 1407 (s), 1334 (s), 1269 (s), 1146 (m), 1108 (vs), 915 (m), 855 (m), 754 (vs), 702 (m) cm^−1^. C_22_H_31_N_3_O_2_S (401.56): calcd. C 65.80, H 7.78, N 10.46, S 7.98; found C 65.71, H 7.90, N 10.59, S 7.84.

(*R*,*R*)-3-Benzyloxy-1-(2-morpholinocyclohexyl)-4,5-diphenyl-1,3-dihydro-2*H*-imidazole-2-thione ((*R*,*R*)-**9h**): Yield 213 mg (81%). Colorless crystals. M.p. 279–281 °C (MeOH/CH_2_Cl_2_) (decomp.). ${\left[\alpha \right]}_{D}^{25}$ = −43.58 (*c* 0.40, CHCl_3_). ^1^H-NMR (CDCl_3_): δ = (signals for both rotamers in ca. 1:1.2 ratio) 7.07–7.43 (m, 15H), 5.40 and 5.33 (d, *J* = 9.4 Hz, 1H), 5.37 and 4.72 (td, *J* = 11.8, 3.3 Hz, 1H), 5.09 and 4.92 (d, *J* = 9.4 Hz, 1H), 3.75 and 3.43 (td, *J* = 11.7, 3.7 Hz, 1H), 3.44–3.69 (m, 4H), 2.78 and 2.43 (br., 2H), 2.09–2.28 (m, 3H), 1.87–1.93 and 2.01–2.06 (m, 1H), 1.66–1.78 (m, 3H), 1.40–1.49 (m, 1H), 1.20–1.27 and 0.92–1.00 (m, 1H), 1.09–1.17 and 0.81–0.89 (m, 1H) ppm. ^13^C-NMR (CDCl_3_): δ = (signals for both rotamers) 157.9, 156.9, 133.13, 133.07, 132.5, 130.6, 130.5, 130.4, 129.6, 129.5, 129.4, 129.3, 129.1, 129.04, 129.02, 128.8, 128.3, 128.0, 127.9, 126.5, 126.34, 126.29, 125.3, 124.5, 77.72, 77.66, 68.0, 67.8, 63.8, 59.8, 59.47, 59.43, 48.3, 33.2, 29.6, 25.93, 25.75, 25.2, 25.0, 24.9, 23.7 ppm. IR: ν 2924 (br m), 2853 (m), 1401 (m), 1360 (w), 1323 (m), 1112 (s), 965 (m), 861 (m), 762 (s), 699 (vs) cm^−1^. C_32_H_35_N_3_O_2_S (525.70): calcd. C 73.11, H 6.72, N 7.99; S 6.10; found C 73.12, H 6.76, N 7.93, S 6.03.

### 4.7. Synthesis of Imidazole N-Oxides ***10a**–**d***

A solution of diacetyl monooxime (**5a**, 354 mg, 3.5 mmol) and the corresponding formaldimine **4** (3.0 mmol) in EtOH (10 mL) was refluxed for 3 h. The solvents were removed in vacuo, and the resulting oil was washed with Et_2_O (3 × 20 mL). The crude product **10** was either purified by column chromatography on silica gel using AcOEt/MeOH mixtures as the eluent or by recrystallization from the appropriate solvents to give spectroscopically pure imidazole *N*-oxides isolated as colorless materials. Compounds **10a** [21] and **10c** [22] were prepared following the analogous method, while imidazole *N*-oxide **10d** was obtained in two steps by condensation of **5a** with methyl glycinate-derived formaldimine of type **4** followed by aminolysis of the resulting product with α-MBA as described [22]; the NMR spectra of the obtained samples matches the data reported in the literature.

(*R*)-1-(2-Hydroxy-1-phenylethyl)-4,5-dimethylimidazole 3-oxide (**10b**): Yield 474 mg (68%). Off-white solid. M.p. 198–200 °C (CH_2_Cl_2_/Et_2_O) (decomp.). ${\left[\alpha \right]}_{D}^{20}$ = +49.1 (*c* 0.22, CHCl_3_). ^1^H-NMR (CDCl_3_): δ = 9.17 (s, 1H, C(2)H), 8.72 (br.s, 1H, OH), 7.01–7.04, 7.27–7.35 (2m, 2H, 3H, Ph), 5.14 (dd, *J* = 3.8, 10.7 Hz, 1H, C*H*Ph), 4.21 (dd, *J* = 10.7, 13.4 Hz, 1H, CH_2_O), 3.95 (dd, *J* = 3.8, 13.4 Hz, 1H, C*H*_2_O), 1.84, 2.10 (2s, 3H each, 2Me) ppm. ^13^C-NMR (CDCl_3_): δ = 136.6 (s, Ph), 128.3, 129.0 (2d, 3CH, Ph), 126.9 (d, 2CH, Ph), 126.4 (s, C(4)), 124.9 (d, C(2)), 122.0 (s, C(5)), 63.9 (d, *C*HPh), 63.6 (t, CH_2_O), 7.0, 8.9 (2q, 2Me) ppm. IR (neat): ν 3110 (s), 3025–2926 (br, m), 1450 (m), 1405 (m), 1351 (s), 1325 (m), 1208 (m), 1064 (s) cm^−1^. ESI-MS (*m*/*z*): 465.4 (100, [2M + H]^+^), 233.3 (47, [M + H]^+^). C_13_H_16_N_2_O_2_ (232.28): calcd. C, 67.22; H, 6.94; N, 12.06; found: C 67.05, H 6.95, N 12.22.

### 4.8. Synthesis of Imidazolium Bromides ***11a**–**d***

To a solution of imidazole *N*-oxide **10** (1.0 mmol) in dry CH_2_Cl_2_ (1.0 mL) was added an excess of 1-bromobutane (548 mg, 4.0 mmol) and the resulting mixture was stirred until the starting material was fully consumed (TLC monitoring: SiO_2_, EtOAc/MeOH 7:1). The solvents were removed under reduced pressure to give the corresponding imidazolium bromide **11** quantitatively, which was used for the next step without further purification.

(*S*)-3-Butoxy-1-(2-hydroxypropyl)-4,5-dimethylimidazolium bromide [23] (**11a**): Reaction time: 2 d. Pale yellow oil. ${\left[\alpha \right]}_{D}^{20}$ = +16.0 (*c* 0.74, CHCl_3_). ^1^H-NMR (CDCl_3_): δ = 10.00 (s, 1H, C(2)H), 4.91 (br.d, *J* ≈ 5.9 Hz, 1H, OH), 4.47 (td, *J* = 2.1, 6.5 Hz, 2H, OCH_2_, Bu), 4.34 (dd, *J* = 9.1, 14.0 Hz, 1H, NCH_2_), 4.22 (dd, *J* = 2.6, 14.0 Hz, 1H, NCH_2_), 4.16–4.22 (m, 1H, C*H*CH_3_), 2.26, 2.27 (2s, 3H each, 2Me), 1.77–1.81 (m, 2H, Bu), 1.47–1.43 (m, 2H, Bu), 1.34 (d, *J* = 6.3 Hz, 3H, CHC*H*_3_), 0.98 (t, *J* = 7.4 Hz, 3H, CH_3_, Bu) ppm. ^13^C-NMR (CDCl_3_): δ = 131.4 (br.d, C(2)), 123.6, 124.7 (2s, C(4), C(5)), 82.7 (t, OCH_2_, Bu), 64.8 (d, *C*HCH_3_), 53.5 (t, NCH_2_), 29.6 (t, CH_2_, Bu), 20.4 (q, CH*C*H_3_), 18.7 (t, CH_2_, Bu), 13.6 (q, CH_3_, Bu), 7.1, 8.9 (2q, 2Me) ppm. IR (neat): ν3308 (s), 2990–2876 (br, s), 1670 (m), 1634 (m), 1545 (m), 1457 (m), 1377 (m), 1139 (s), 1072 (s), 936 (s) cm^−1^.

(*R*)-3-Butoxy-1-(2-hydroxy-1-phenylethyl)-4,5-dimethylimidazolium bromide (**11b**): Reaction time: 2 d. Yellow solid. M.p. 124–127 °C. ${\left[\alpha \right]}_{D}^{25}$ = +83.5 (*c* 1.79, CHCl_3_). ^1^H-NMR (CDCl_3_): δ = 10.50 (s, 1H, C(2)H), 7.15–7.18, 7.32–7.38 (2m, 2H, 3H, Ph), 5.61 (dd, *J* = 3.8, 10.1 Hz, 1H, C*H*Ph), 5.46 (br.s, 1H, OH), 4.80 (*pseudo*-q, *J* ≈ 6.6 Hz, 1H, OCH_2_, Bu), 4.52–4.59 (m, 2H, C*H*_2_OH (1H), OCH_2_ (1H)), 4.10 (dd, *J* = 3.8, 13.2 Hz, 1H, C*H*_2_OH), 2.06, 2.24 (2s, 3H each, 2Me), 1.79–1.84 (m, 2H, Bu), 1.49–1.55 (m, 2H, Bu), 0.98 (t, *J* = 7.4 Hz, 3H, CH_3_, Bu) ppm. ^13^C-NMR (CDCl_3_): δ = 134.2 (s, Ph), 131.4 (d_br_, C(2)), 126.7, 129.3, 129.5 (3d, 5CH, Ph), 124.3, 125.0 (2s, C(4), C(5)), 83.2 (t, OCH_2_, Bu), 64.9 (d, *C*HPh), 62.4 (t, CH_2_OH), 29.8 (t, CH_2_, Bu), 18.8 (t, CH_2_, Bu), 13.7 (q, CH_3_, Bu), 7.1, 9.1 (2q, 2Me) ppm. IR (neat): ν 3248 (vs), 3027–2876 (br, s), 1636 (m), 1541 (m), 1448 (s), 1359 (m), 1066 (s), 935 (s) cm^−1^.

(*R*)-3-Butoxy-4,5-dimethyl-1-[1-(methoxycarbonyl)ethyl]imidazolium bromide [23] (**11c**): Reaction time: 2 d. Pale yellow oil. ${\left[\alpha \right]}_{D}^{20}$ = −16.6 (*c* 0.51, CHCl_3_). The NMR data in accordance with those reported in [23].

(*R*)-3-Butoxy-4,5-dimethyl-1-[(*N*-phenylethyl)acetamido]imidazolium bromide (**11d**): Reaction time: 4 d. Pale yellow oil. ${\left[\alpha \right]}_{D}^{20}$ = +51.2 (*c* 0.52, CHCl_3_). ^1^H-NMR (CDCl_3_): δ = 9.84 (s, 1H, C(2)H), 9.29 (d, *J* = 7.8 Hz, 1H, NH), 7.14–7.17, 7.23–7.26, 7.39–7.42 (3m, 1H, 2H, 2H, Ph), 5.35, 5.45 (AB system, *J* = 16.1 Hz, 2H, NCH_2_), 4.95 (dq, *J* = 7.1, 7.8 Hz, 1H, C*H*(Ph)CH_3_), 4.32 (td, *J* = 1.4, 6.5 Hz, 2H, OCH_2_, Bu), 2.14, 2.17 (2s, 3H each, 2Me), 1.71–1.76 (m, 2H, Bu), 1.53 (d, *J* = 7.1 Hz, 3H, CH(Ph)C*H*_3_), 1.42–1.48 (m, 2H, Bu), 0.94 (t, *J* = 7.4 Hz, 3H, CH_3_, Bu) ppm. ^13^C-NMR (CDCl_3_): δ = 163.6 (s, C=O), 143.6 (s, Ph), 131.4 (br.d, C(2)), 126.3, 126.9, 128.4 (3d, 5CH, Ph), 123.3, 125.9 (2s, C(4), C(5)), 82.6 (t, OCH_2_, Bu), 50.3 (d, *C*H(Ph)CH_3_), 49.7 (t, NCH_2_), 29.5 (t, CH_2_, Bu), 22.4 (q, CH(Ph)*C*H_3_), 18.6 (t, CH_2_, Bu), 13.6 (q, CH_3_, Bu), 7.0, 8.7 (2q, 2Me) ppm. IR (neat): ν 3200 (m), 3032–2872 (br, s), 1679 (vs, C=O), 1547 (s), 1448 (m), 1377 (m), 1250 (m) cm^−1^.

### 4.9. Synthesis of Imidazole-2-thiones ***13a**–**d*** and ***15a**–**d***

To a solution of the respective imidazolium bromide (1.0 mmol) in dry pyridine (4.0 mL) was added triethylamine (150 μL) followed by elemental sulfur (33 mg, 1.1 mmol), and the resulting mixture was stirred overnight. The solvents were removed under reduced pressure, and the crude product was purified by standard column chromatography to give imidazole-2-thione of type **13** or **15**, respectively.

(*S*)-3-Butoxy-1-(2-hydroxypropyl)-4,5-dimethylimidazole-2-thione (**13a**): (chromatographic separation, SiO_2_, CH_2_Cl_2_/EtOAc 5:1); 209 mg (81%). Pale yellow oil. ${\left[\alpha \right]}_{D}^{20}$ = +1.3 (*c* 0.77, CHCl_3_). ^1^H-NMR (CDCl_3_): δ = 4.29–4.34 (m, 2H, OCH_2_, Bu), 4.18–4.23 (m, 1H, C*H*CH_3_), 4.03 (dd, *J* = 8.7, 14.4 Hz, 1H, NCH_2_), 3.97 (dd, *J* = 3.2, 14.4 Hz, 1H, NCH_2_), 3.28 (br.s, 1H, OH), 2.08, 2.12 (2s, 3H each, 2Me), 1.73–1.77 (m, 2H, Bu), 1.46–1.53 (m, 2H, Bu), 1.25 (d, *J* = 6.3 Hz, 3H, CHC*H*_3_), 0.96 (t, *J* = 7.4 Hz, 3H, CH_3_, Bu) ppm. ^13^C-NMR (CDCl_3_): δ = 156.2 (s, C=S), 118.5, 119.0 (2s, C(4), C(5)), 76.8 (t, OCH_2_, Bu), 67.3 (d, *C*HCH_3_), 51.3 (t, NCH_2_), 29.8 (t, CH_2_, Bu), 21.2 (q, CH*C*H_3_), 18.9 (t, CH_2_, Bu), 13.7 (q, CH_3_, Bu), 7.5, 9.1 (2q, 2Me) ppm. IR (neat): ν 3353 (s), 3040–2874 (br, m), 1433 (m), 1409 (s), 1375 (m), 1347 (m), 1127 (m), 1072 (m), 943 (m) cm^−1^. ESI-MS (*m*/*z*): 281.1 (53, [M + Na]^+^), 259.1 (100, [M + H]^+^), 208.1 (42). C_12_H_22_N_2_O_2_S (258.38): calcd. C 55.78, H 8.58, N 10.84, S 12.41; found: C 55.57, H 8.65, N 10.76, S 12.21.

(*R*)-3-Butoxy-1-(2-hydroxy-1-phenylethyl)-4,5-dimethylimidazole-2-thione (**13b**): (chromatographic separation, SiO_2_, CH_2_Cl_2_/EtOAc 5:1); 240 mg (75%). Yellow oil. ${\left[\alpha \right]}_{D}^{20}$ = −10.8 (*c* 0.73, CHCl_3_). ^1^H-NMR (CDCl_3_): δ = 7.24–7.30, 7.32–7.35 (2m, 3H, 2H, Ph), 6.26 (m, 1H, C*H*Ph), 4.60–4.65 (m, 1H, C*H*_2_OH), 4.34–4.42 (m, 3H, OCH_2_(Bu) (2H), C*H*_2_OH (1H)), 3.09 (br.s, 1H, OH), 2.09 (s, 3H, Me), 1.79–1.84 (m, 2H, Bu), 1.72 (s, 3H, Me), 1.52–1.58 (m, 2H, Bu), 1.00 (t, *J* = 7.4 Hz, 3H, CH_3_, Bu) ppm. ^13^C-NMR (CDCl_3_): δ = 157.4 (s, C=S), 136.7 (s, Ph), 126.9, 127.8, 128.7 (3d, 5CH, Ph), 118.8, 120.1 (2s, C(4), C(5)), 76.8 (t, OCH_2_, Bu), 62.8 (t, CH_2_OH), 60.9 (d, *C*HPh), 29.9 (t, CH_2_, Bu), 19.0 (t, CH_2_, Bu), 13.8 (q, CH_3_, Bu), 7.4, 9.9 (2q, 2Me) ppm. IR (neat): ν 3347 (s), 3016–2874 (br, s), 1407 (s), 1333 (m), 1156 (m), 1060 (m), 1031 (m) cm^–1^. ESI-MS (*m*/*z*): 343.1 (54, [M + Na]^+^), 321.2 (100, [M + H]^+^), 270.1 (45). HRMS (ESI-TOF): calcd for C_17_H_25_N_2_O_2_S: 321.1637; found: 321.1640.

(*R*)-3-Butoxy-4,5-dimethyl-1-[1-(methoxycarbonyl)ethyl]imidazole-2-thione (**13c**): (chromatographic separation, SiO_2_, CH_2_Cl_2_/EtOAc 95:5); 236 mg (83%). Yellow oil. ${\left[\alpha \right]}_{D}^{20}$ = −45.4 (*c* 0.20, CHCl_3_). ^1^H-NMR (CDCl_3_): δ = 6.03 (d, *J* = 7.4 Hz, 1H, C*H*CH_3_), 4.31–4.39 (m, 2H, OCH_2_, Bu), 3.74 (s, 3H, OCH_3_), 2.02, 2.12 (2s, 3H each, 2Me), 1.75–1.80 (m, 2H, Bu), 1.62 (d, *J* = 7.4 Hz, 3H, CHC*H*_3_), 1.49–1.55 (m, 2H, Bu), 0.98 (t, *J* = 7.4 Hz, 3H, CH_3_, Bu) ppm. ^13^C-NMR (CDCl_3_): δ = 170.8 (s, C=O), 157.6 (s, C=S), 117.4, 119.8 (2s, C(4), C(5)), 76.7 (t, OCH_2_, Bu), 53.2 (d, *C*HCH_3_), 52.6 (q, OCH_3_), 29.9 (t, CH_2_, Bu), 19.0 (t, CH_2_, Bu), 16.0 (q, CH*C*H_3_), 13.8 (q, CH_3_, Bu), 7.4, 9.8 (2q, 2Me) ppm. IR (neat): ν 2932–2872 (m), 1743 (vs, C=O), 1407 (s), 1344 (m), 1224 (s), 1183 (s), 1109 (m), 1066 (s), 963 (m) cm^−1^. ESI-MS (*m*/*z*): 287.3 (100, [M + H]^+^). C_13_H_22_N_2_O_3_S (286.39): calcd. C 54.52, H 7.74, N, 9.78, S 11.19; found: C 54.58, H 7.79, N 9.77, S 11.18.

(*R*)-3-Butoxy-4,5-dimethyl-1-[(*N*-phenylethyl)acetamido]imidazole-2-thione (**13d**): (chromatographic separation, SiO_2_, CH_2_Cl_2_/EtOAc 4:1); 238 mg (66%). Pale orange solid. M.p. 118–119 °C. ${\left[\alpha \right]}_{D}^{20}$ = +2.7 (*c* 1.89, CHCl_3_). ^1^H-NMR (CDCl_3_): δ = 7.71 (br.d, *J* ≈ 8.1 Hz, 1H, NH), 7.20–7.23, 7.25–7.30 (2m, 1H, 4H, Ph), 5.00 (*pseudo*-p, *J* ≈ 7.1 Hz, 1H, C*H*(Ph)CH_3_), 4.65, 4.70 (AB system, *J* = 14.9 Hz, 2H, NCH_2_), 4.35 (dt, *J* ≈ 6.6, 8.1 Hz, 1H, OCH_2_, Bu), 4.28 (dt, *J* ≈ 6.6, 8.1 Hz, 1H, OCH_2_, Bu), 2.11, 2.13 (2s, 3H each, 2Me), 1.76–1.81 (m, 2H, Bu), 1.50–1.56 (m, 2H, Bu), 1.43 (d, *J* = 7.0 Hz, 3H, CH(Ph)C*H*_3_), 0.99 (t, *J* = 7.4 Hz, 3H, CH_3_, Bu) ppm. ^13^C-NMR (CDCl_3_): δ = 166.2 (s, C=O), 156.3 (s, C=S), 143.1 (s, Ph), 125.8, 127.0, 128.4 (3d, 5CH, Ph), 118.5, 119.5 (2s, C(4), C(5)), 76.9 (t, OCH_2_, Bu), 49.3 (d, *C*H(Ph)CH_3_), 48.6 (t, NCH_2_), 29.8 (t, CH_2_, Bu), 22.5 (q, CH(Ph)*C*H_3_), 18.9 (t, CH_2_, Bu), 13.7 (q, CH_3_, Bu), 7.4, 9.1 (2q, 2Me) ppm. IR (neat): ν 3282 (m), 2956–2871 (br, m), 1662 (vs, C=O), 1549 (s), 1407 (s), 1372 (m), 1247 (m), 1003 (m) cm^−1^. ESI-MS (*m*/*z*): 384.2 (100, [M + Na]^+^), 362.2 (21, [M + H]^+^), 311.1 (48), 241.1 (69). C_19_H_27_N_3_O_2_S (361.50): calcd. C 63.13, H 7.53, N 11.62, S 8.87; found: C 63.04, H 7.80, N 11.70, S 8.76.

(*S*)-3-Benzyloxy-4,5-dimethyl-1-(1-phenylethyl)imidazole-2-thione (**15a**): Yield: 275 mg (81%). Pale yellow crystals. M.p. 102–104 °C (petroleum ether/CH_2_Cl_2_). ${\left[\alpha \right]}_{D}^{20}$ = +110.5 (*c* 0.32, CHCl_3_). ^1^H-NMR (CDCl_3_): δ = 7.58–7.53 (m, 2H), 7.43–7.40 (m, 3H), 7.37–7.34 (m, 2H), 7.30–7.26 (m, 3H), 6.80 (q, *J* = 7.3 Hz, 1H), 5.58, 5.45 (AB system, *J* = 10.0 Hz, 2H, OCH_2_), 1.83 (d, *J* = 7.3 Hz, 3H), 1.78, 1.63 (2s, 6H, 2Me) ppm. ^13^C-NMR (CDCl_3_): δ = 157.3 (s, C=S), 126.4, 127.4, 128.5, 128.6, 129.3, 130.4 (6d, 10CH), 117.5, 120.7 134.0, 139 (4s), 77.9 (t, OCH_2_), 53.4 (d, CH), 7.2, 10.2, 17.3 (3q, 3Me) ppm. IR (neat): ν 2971 (m), 2924 (m), 1449 (m), 1403 (s), 1375 (m), 1332 (s), 1299 (m), 1151 (m), 1025 (m), 956 (m), 909 (m), 747 (s), 697 (vs) cm^−1^. HRMS (ESI-TOF) calcd for C_20_H_23_N_2_OS: 339.1531; found: 339.1535.

(*S*)-4,5-Dimethyl-3-pentyloxy-1-(1-phenylethyl)imidazole-2-thione (**15b**): Yield: 245 mg (77%). Pale yellow crystals. M.p. 70–73 °C (petroleum ether/CH_2_Cl_2_). ${\left[\alpha \right]}_{D}^{20}$ = +125.9 (*c* 0.40, CHCl_3_). ^1^H-NMR (CDCl_3_): δ = 7.28–7.39 (m, 5H), 6.76 (q, *J =* 7.3 Hz, 1H), 4.43–4.46 (pseudo q, *J* = 7.2 Hz, 1H, OCH_2_), 4.37–4.40 (pseudo q, *J* = 7.2 Hz, 1H, OCH_2_), 2.09 (*s*, 3H, Me), 1.83–1.88 (m, 2H, CH_2_), 1.80 (d, *J =* 7.3 Hz, 3H), 1.68 (s, 3H, Me), 1.49–1.53 (m, 2H, CH_2_), 1.41–1.45 (m, 2H, CH_2_), 0.96 (t, *J* = 7.3 Hz, 3H, CH_3_) ppm. ^13^C-NMR (CDCl_3_): δ = 157.5 (C=S), 126.5, 127.3, 128.5 (3d, 5CH), 117.8, 119.9, 139.9 (3s), 76.9 (t, OCH_2_), 53.4 (d, CH), 22.5, 27.7, 27.9 (3t, 3CH_2_), 7.3, 10.1, 13.9, 16.8 (4q, 4Me) ppm. IR (neat): ν 2954 (m), 2927 (m), 1450 (m), 1410 (s), 1334 (s), 1299 (s), 1157 (m), 1027 (m), 1001 (s), 998 (m), 876 (m), 700 (vs), 687 (s) cm^−1^. C_18_H_26_N_2_OS (318.48): calcd. C 67.88, H 8.23, N 8.80, S 10.07; found: C 67.61, H 8.28, N 8.70, S 10.06.

(*S*)-3-Benzyloxy-4,5-diphenyl-1-(1-phenylethyl)imidazole-2-thione (**15c**): Yield: 305 mg (66%). Pale yellow oil. ${\left[\alpha \right]}_{D}^{20}$ = +188.5 (*c* 0.40, CHCl_3_). ^1^H-NMR (CDCl_3_): δ = 7.30–7.32 (m, 2H), 7.20–7.25 (m, 10H), 7.14–7.18 (m, 2H), 7.05–7.12 (m, 6H), 6.75 (br.s, 1H, CH_3_-C*H*), 6.67 (br.s, 2H), 5.30, 5.32 (AB system, *J =* 12.5 Hz, 2H, OCH_2_), 1.62 (d, *J* = 6.5 Hz, 3H, C*H*_3_-CH) ppm. ^13^C-NMR (CDCl_3_): δ = 158.4 (s, C=S), 124.1, 125.9, 126.2, 128.6, 129.8, 132.9 (6s), 126.9, 127.3, 127.9, 128.0, 128.1, 128.2, 128.3, 128.9, 129.1, 129.4, 130.6, 132.0 (12d, 20CH), 77.7 (t, OCH_2_), 54.6 (d, CH), 17.5 (q, CH_3_) ppm. IR (neat): ν 3032 (m), 2927 (m), 1496 (m), 1446 (m), 1395 (s), 1325 (m), 1316s, 1215 (m), 1187 (m), 1073 (m), 907 (m), 730 (s), 695 (vs) cm^−1^. HRMS (ESI-TOF): calcd for C_30_H_27_N_2_O: 463.1844; found: 463.1849.

(*S*)-3-Pentyloxy-4,5-diphenyl-1-(1-phenylethyl)imidazole-2-thione (**15d**): Yield: 350 mg (79%). Beige crystals. M.p. 78–81 °C (petroleum ether/CH_2_Cl_2_). ${\left[\alpha \right]}_{D}^{20}$ = +130.1 (*c* 0.30, CHCl_3_). ^1^H-NMR (CDCl_3_): δ = 7.20–7.25 (m, 9H), 7.06–7.09 (m, 4H), 6.67–6.73 (m, 3H, 2CH_arom_, *CH*-CH_3_), 4.31 (pseudo q, *J* = 6.8 Hz, 1H, OCH_2_), 4.14 (pseudo q, *J* = 6.8 Hz, 1H, OCH_2_), 1.63–1.66 (m, 2H, CH_2_), 1.59 (d, *J* = 6.8 Hz, 3H, *CH*_3_-CH), 1.24–1.27 (*m*, 2H, CH_2_), 1.19–1.23 (*m*, 2H, CH_2_), 0.83 (*t*, *J* = 7.3 Hz, 3H, CH_3_) ppm. ^13^C-NMR (CDCl_3_): δ = 158.6 (s, C=S), 126.9, 127.2, 127.9, 128.1, 128.2, 128.3, 128.9, 129.3, 132.0 (9d, 15CH), 124.4, 125.2, 126.0, 140.3 (4s), 76.9 (t, OCH_2_), 54.7 (d, CH), 22.2, 27.4, 27.8 (3t, 3CH_2_), 13.9, 17.5 (2q, 2CH_3_) ppm. IR (neat): ν 2968 (m), 2953 (m), 1448 (m), 1392 (s), 1321 (s), 1187 (m), 1026 (m), 1002 (m), 963 (m), 702 (vs), 695 (vs) cm^−1^. C_28_H_30_N_2_OS (442.62): C 75.98; H 6.83; N 6.33; S 7.24. Found: C 75.78; H 6.88; N 6.58; S 7.50.

## Figures and Tables

**Table 1:** Synthesis of imidazole *N*-oxides **6** and imidazole-2-thiones **9** from chiral formaldimines **4a**–**d** derived from ‘desymetrized’ *trans*-1,2-diaminocyclohexanes **1**.

Config.	4	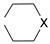	R	6	(%) ^1^	9	(%) ^1^
(*R*,*R*)	**a**	(CH_2_)_4_	Me	**a**	65	**a**	62
(*R*,*R*)	**a**	(CH_2_)_4_	Ph	**b**	93	**b**	70
(*R*,*R*)	**b**	(CH_2_)_5_	Me	**c**	90	**c**	60
(*R*,*R*)	**b**	(CH_2_)_5_	Ph	**d**	65	**d**	82
(*S*,*S*)	**b**	(CH_2_)_5_	Me	**c**	95	**c**	47
(*S*,*S*)	**b**	(CH_2_)_5_	Ph	**d**	83	**d**	62
(*R*,*R*)	**c**	(CH_2_)_6_	Me	**e**	78	**e**	42
(*R*,*R*)	**c**	(CH_2_)_6_	Ph	**f**	89	**f**	70
(*R*,*R*)	**d**	(CH_2_)_2_O(CH_2_)_2_	Me	**g**	86	**g**	67
(*R*,*R*)	**d**	(CH_2_)_2_O(CH_2_)_2_	Ph	**h**	66	**h**	81

^1^ Yield of isolated compounds.

## Supplementary Materials

Copies of ^1^H-NMR and ^13^C-NMR spectra of all new compounds are available online.
