# Supplementary material for: 2-Unsubstituted Imidazole N-Oxides as Novel Precursors of Chiral 3-Alkoxyimidazol-2-ylidenes Derived from trans-1,2-Diaminocyclohexane and Other Chiral Amino Compounds
Source: Molecules. 2019 Dec 2;24(23):4398. doi: 10.3390/molecules24234398 (PMC6930529; doi:10.3390/molecules24234398)
Supplement: Supplementary file 1 [file molecules-24-04398-s001.pdf]

Supporting Information for

# 2-Unsubstituted Imidazole *N*-oxides as Novel Precursors of Chiral 3-Alkoxyimidazol-2-ylidenes Derived from *trans*-1,2-Diaminocyclohexane and Other Chiral Amino Compounds

Grzegorz Mlostoń,[a*] Małgorzata Celeda,[a] Marcin Jasiński,[a] Katarzyna Urbaniak,[a]
Przemysław J. Boratyński,[b] Peter R. Schreiner[c] and Heinz Heimgartner

[a] Department of Organic and Applied Chemistry, University of Łódź, Tamka 12, PL-91-403 Łódź, Poland

[b] Department of Organic Chemistry, Wrocław University of Technology, Wyspiańskiego 27, PL-50-370 Wrocław, Poland.

[c] Justus Liebig University, Institute of Organic Chemistry, Heinrich-Buff-Ring 17, D-35392 Giessen, Germany

[d] Department of Chemistry, University of Zurich, Winterthurerstrasse 190, CH-8057 Zurich, Switzerland

**Content:**

Copies of $^{1}$H NMR and $^{13}$C NMR spectra of synthetized compounds S2–S42

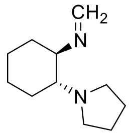


(*R*,*R*)-**4a**

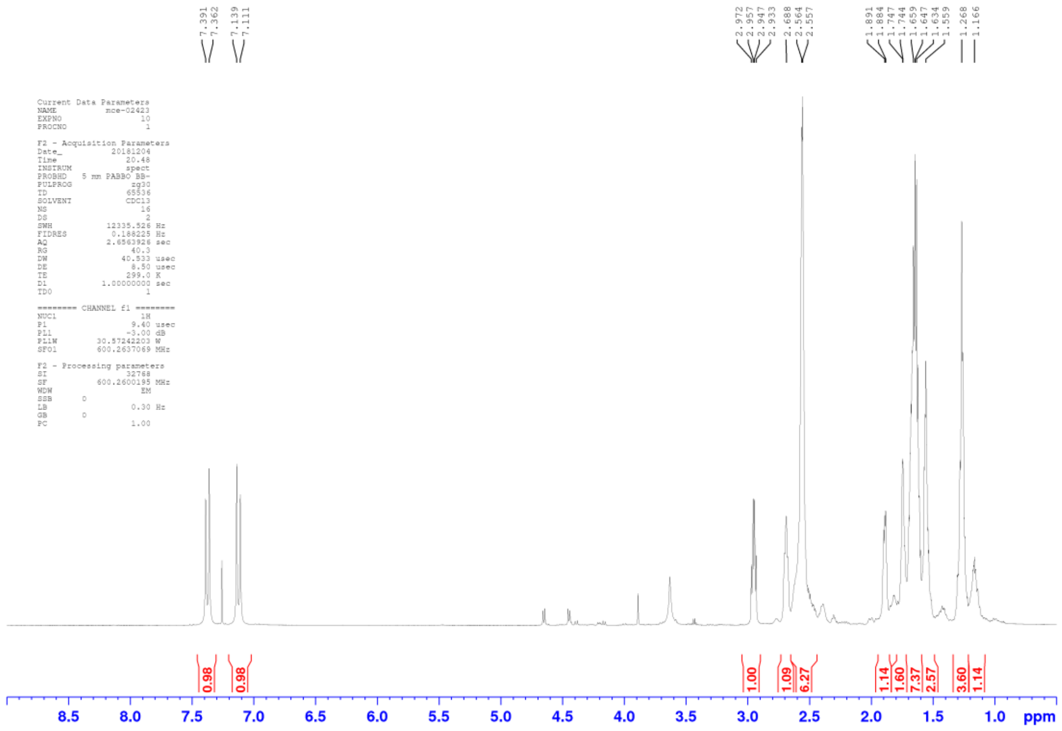


**Figure S1**. $^1$H NMR of (*R*,*R*)-**4a** ($CDCl_3$, 600 MHz).

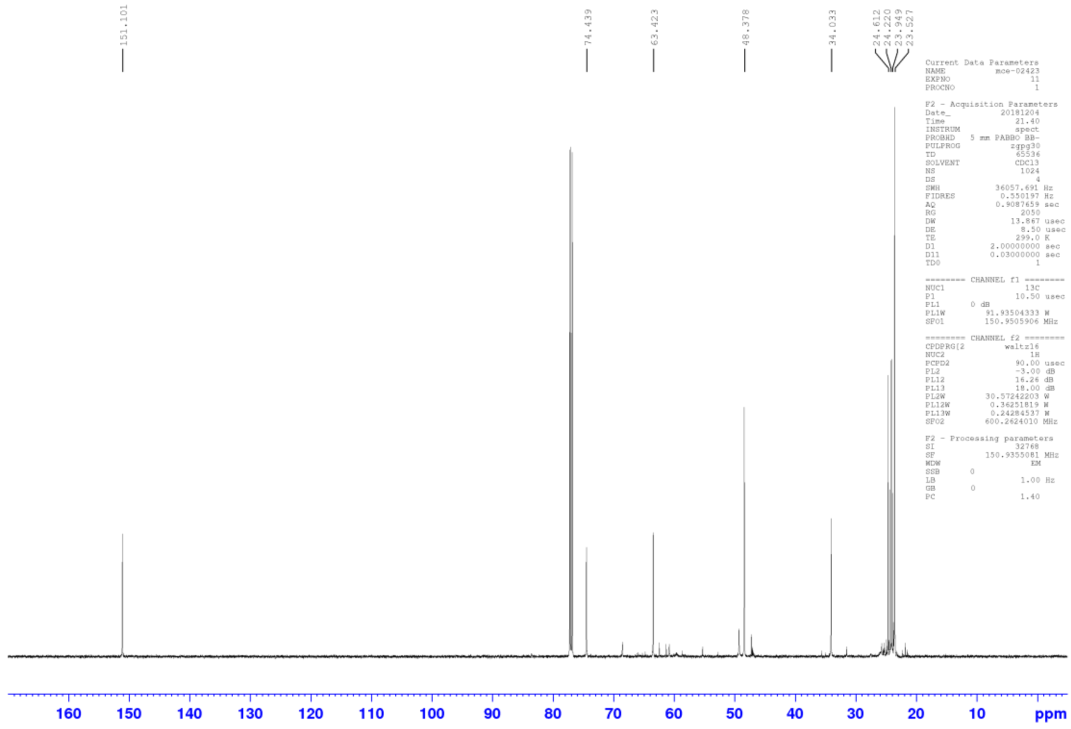


**Figure S2**. $^{13}$C NMR of (*R*,*R*)-**4a** ($CDCl_3$, 151 MHz).

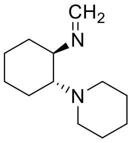


(*R*,*R*)-**4b**

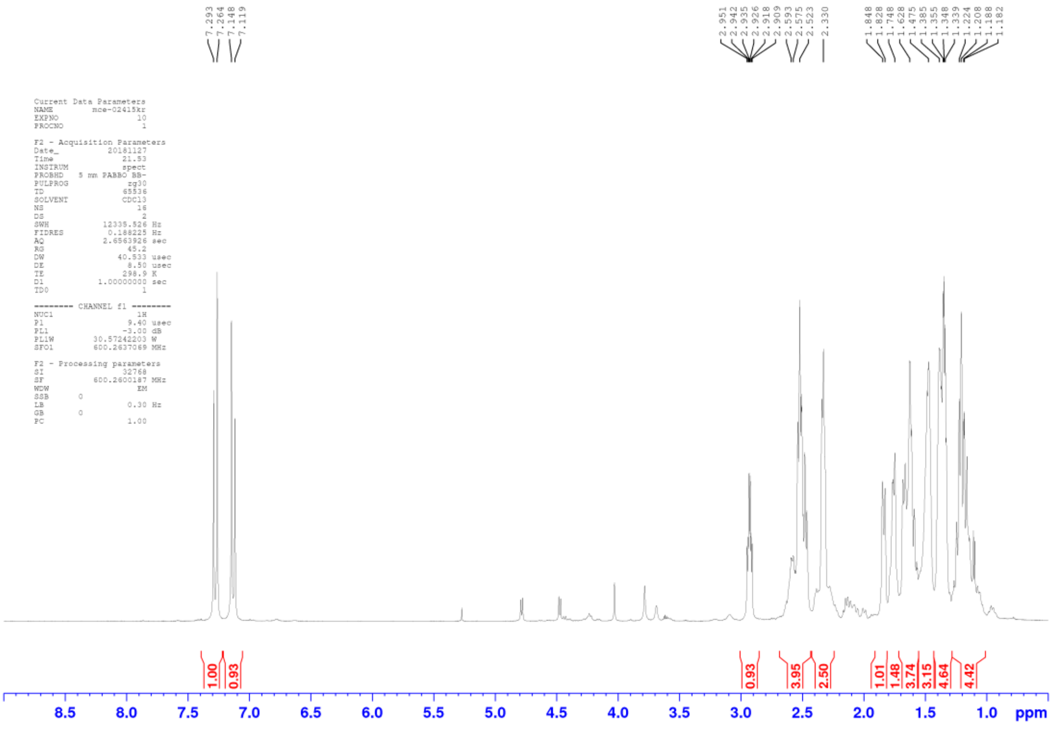


**Figure S3**. $^{1}$H NMR of (*R*,*R*)-**4b** ($CDCl_3$, 600 MHz).

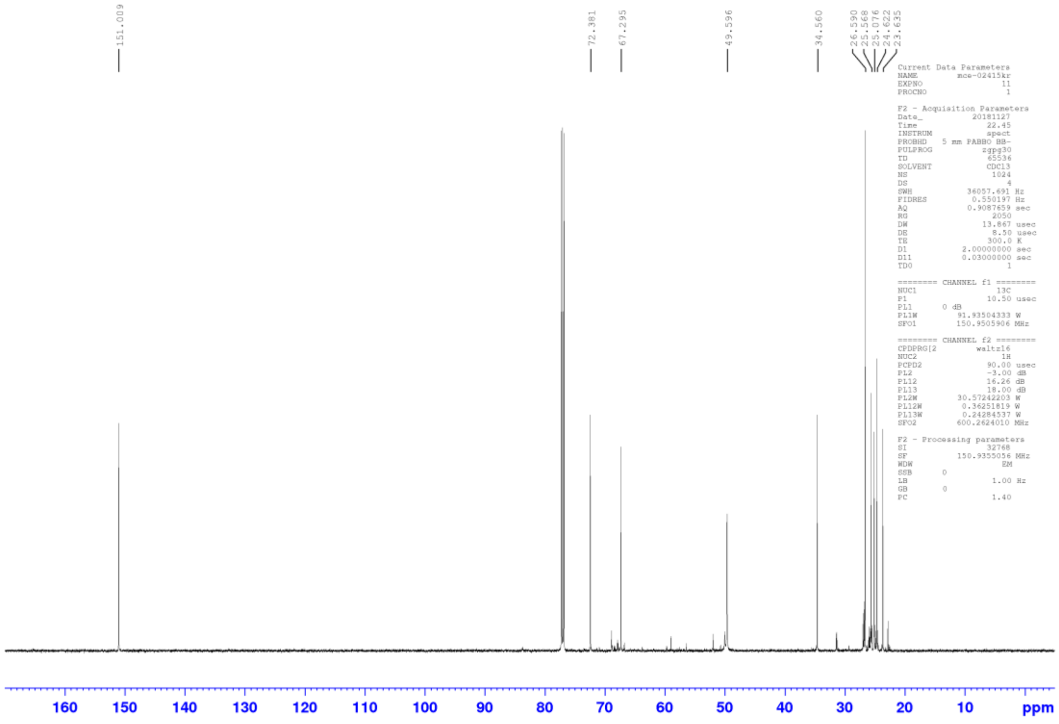


**Figure S4**. $^{13}$C NMR of (*R*,*R*)-**4b** ($CDCl_3$, 151 MHz).

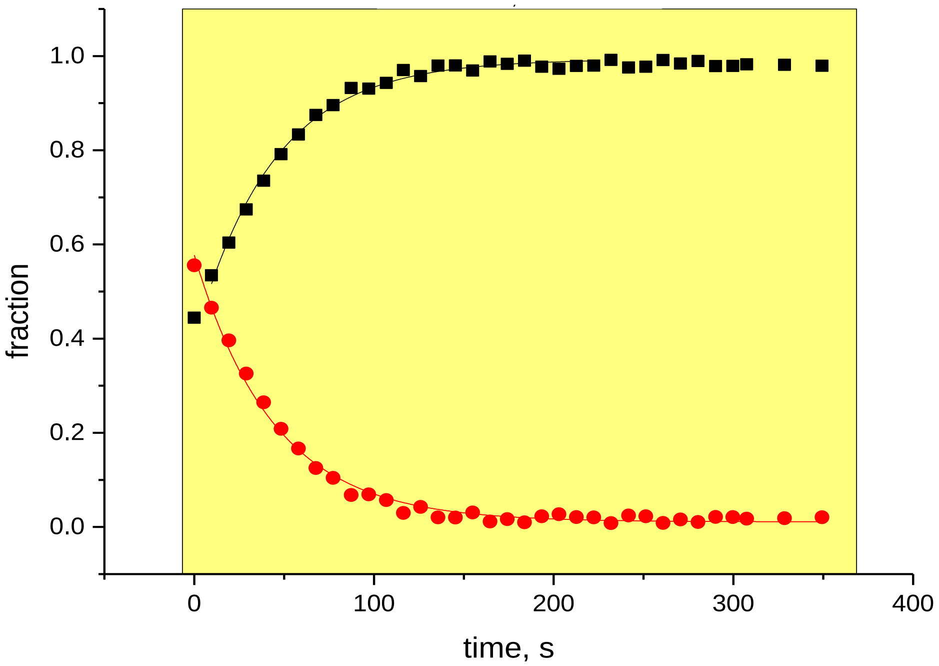


**Figure S5**. Kinetics of triazine-formaldimine **4b**/**4'b** equilibration after dissolution in $CDCl_3$ monitored by $^1$H NMR integration. Content of triazine is shown in red dots, and the content of monomeric formaldimine in black squares. Decay curves were fitted with $t_{1/2}$ value of 30 s.

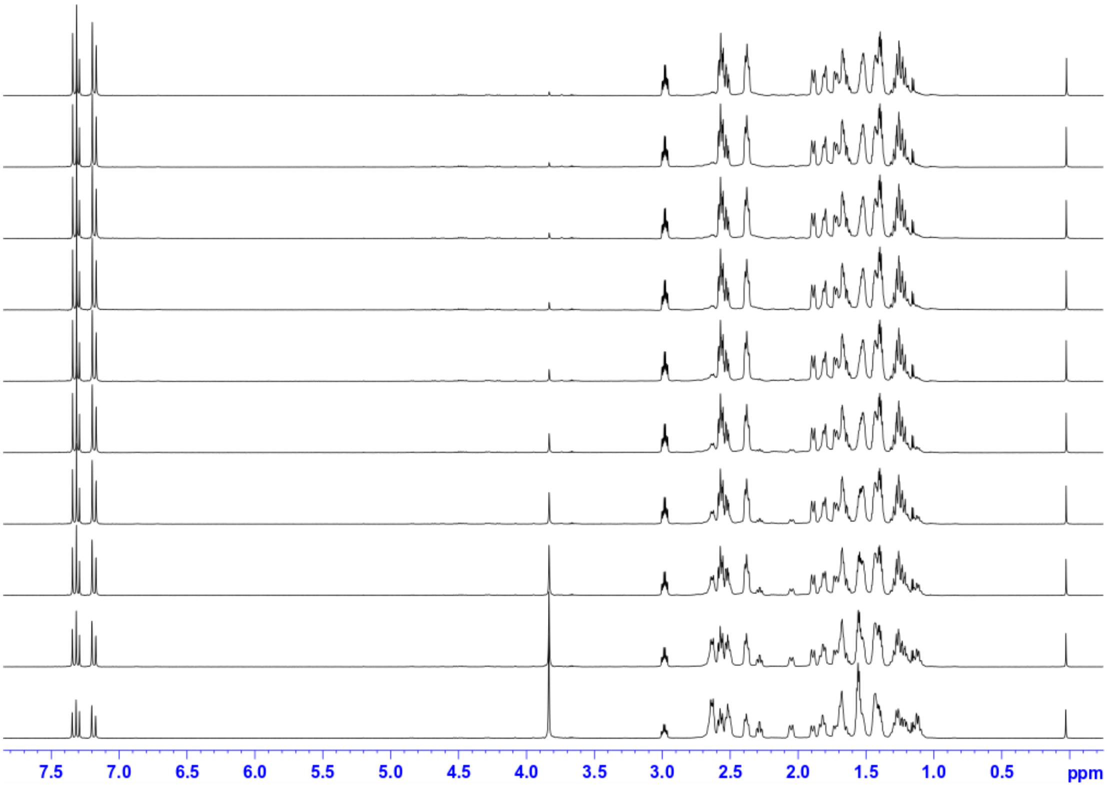


**Figure S6**. Overlays of NMR spectra (600 MHz, $CDCl_3$, NS=1) of **4b**/**4'b** taken at various intervals after dissolution and placing in the instrument and initiating measurement (accounting for approx. 15-20 s). Shown spectra bottom to top were collected after consecutive 19.3 s increments.

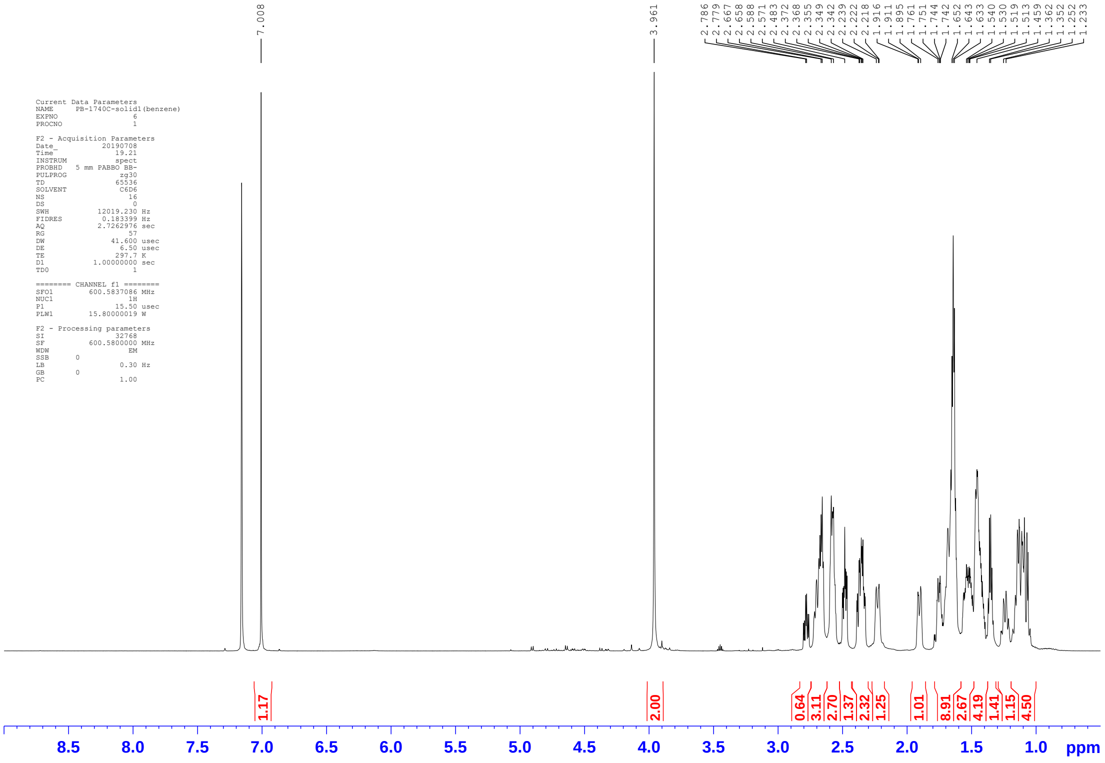


**Figure S7**. $^{1}$H NMR spectrum of **4b**/**4’b** in benzene-$d_6$: triazine to monomeric formaldimine ratio is established at 1:0.6.

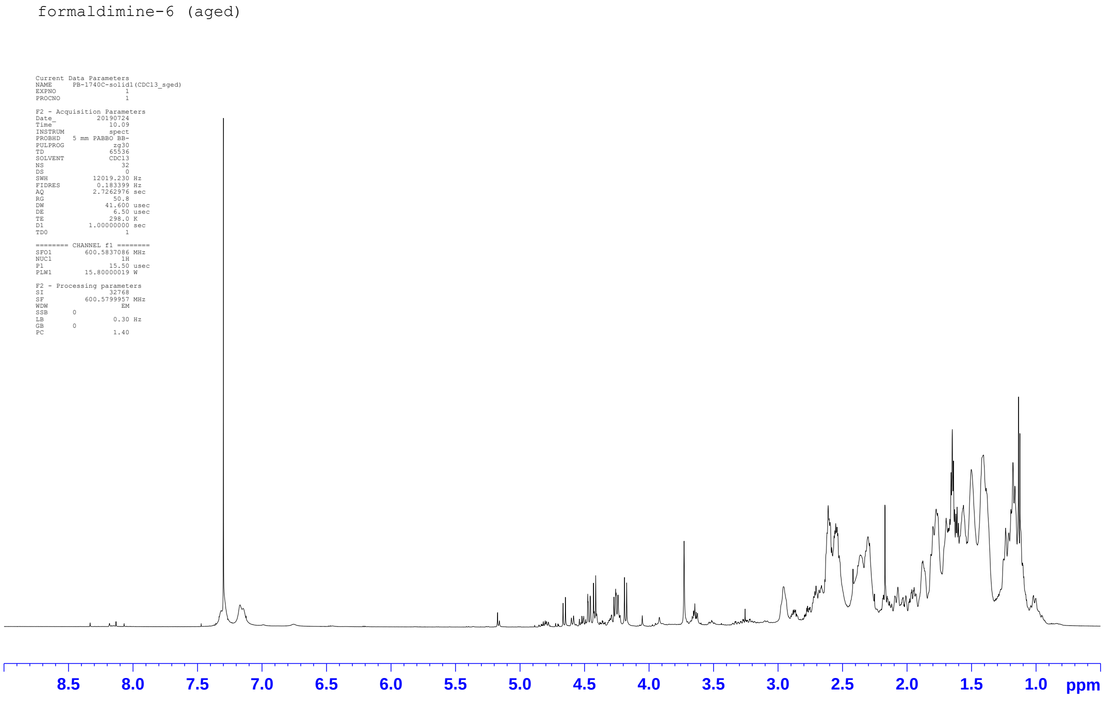


**Figure S8**. $^{1}$H NMR spectrum (600 MHz, $CDCl_3$) of a sample of formaldimine **4b** following storage in the $CDCl_3$ solution for 16 days. Unidentified products account for approx. 75% of the material.

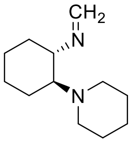

(*S*,*S*)-**4b**

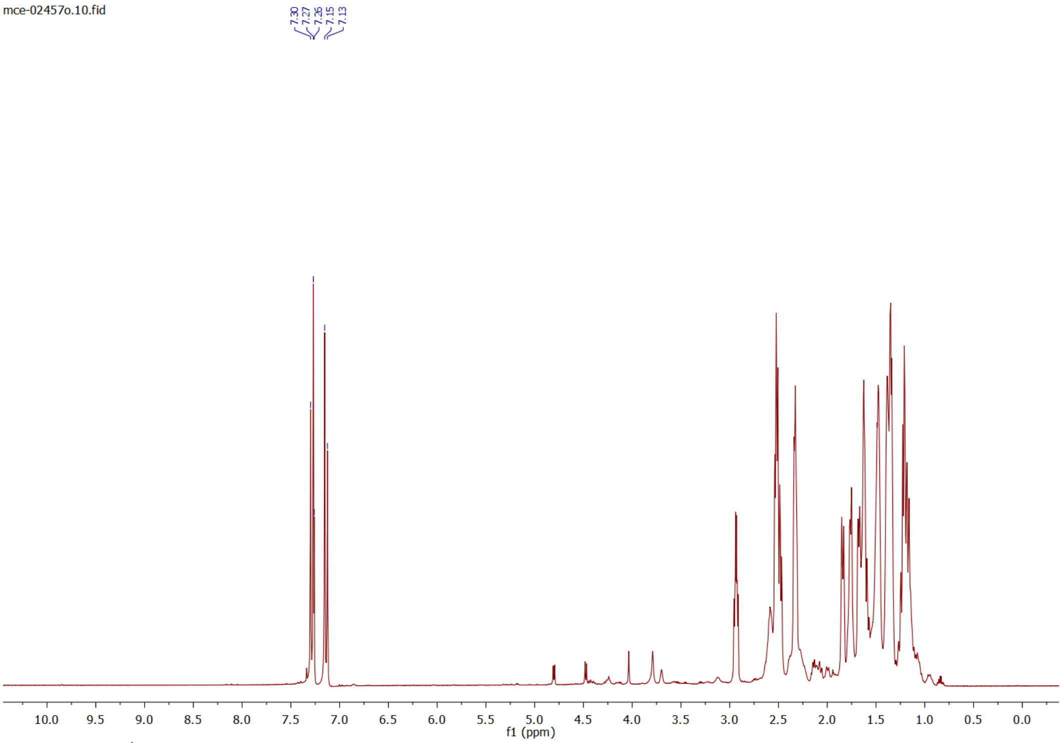


**Figure S9**. $^{1}$H NMR of (*S*,*S*)-**4b** ($CDCl_3$, 600 MHz).

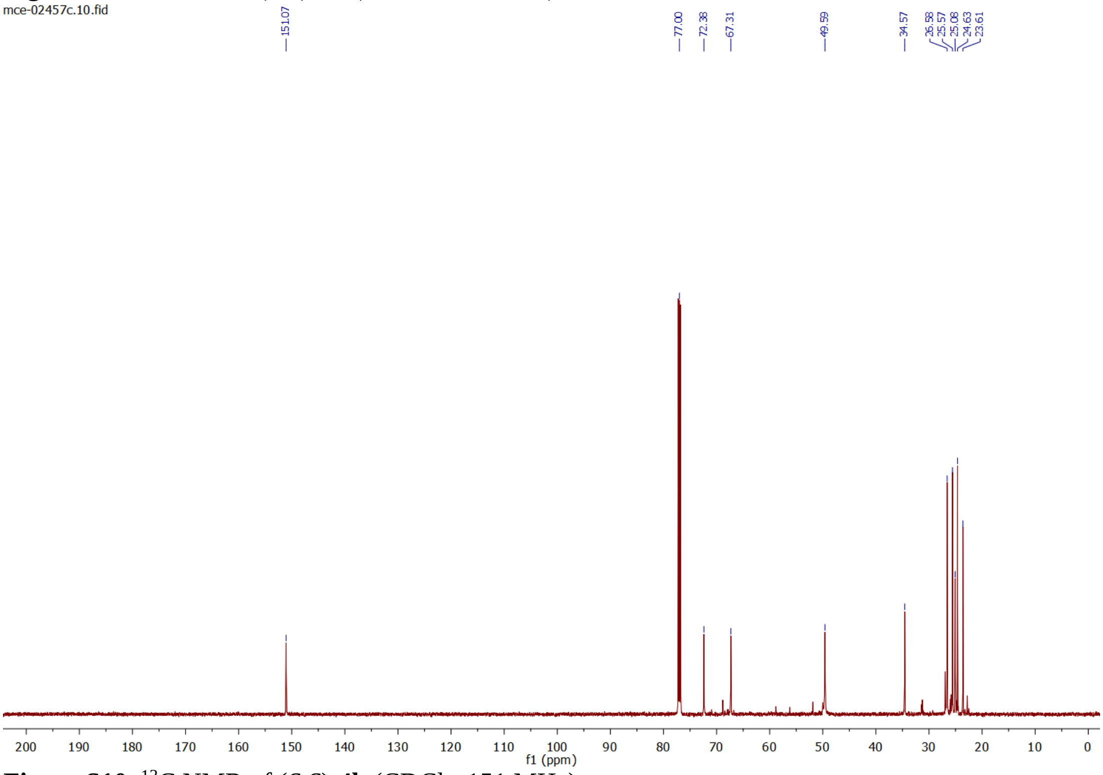


**Figure S10**. $^{13}$C NMR of (*S*,*S*)-**4b** ($CDCl_3$, 151 MHz).

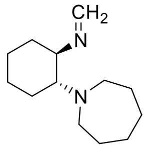


(*R*,*R*)-**4c**

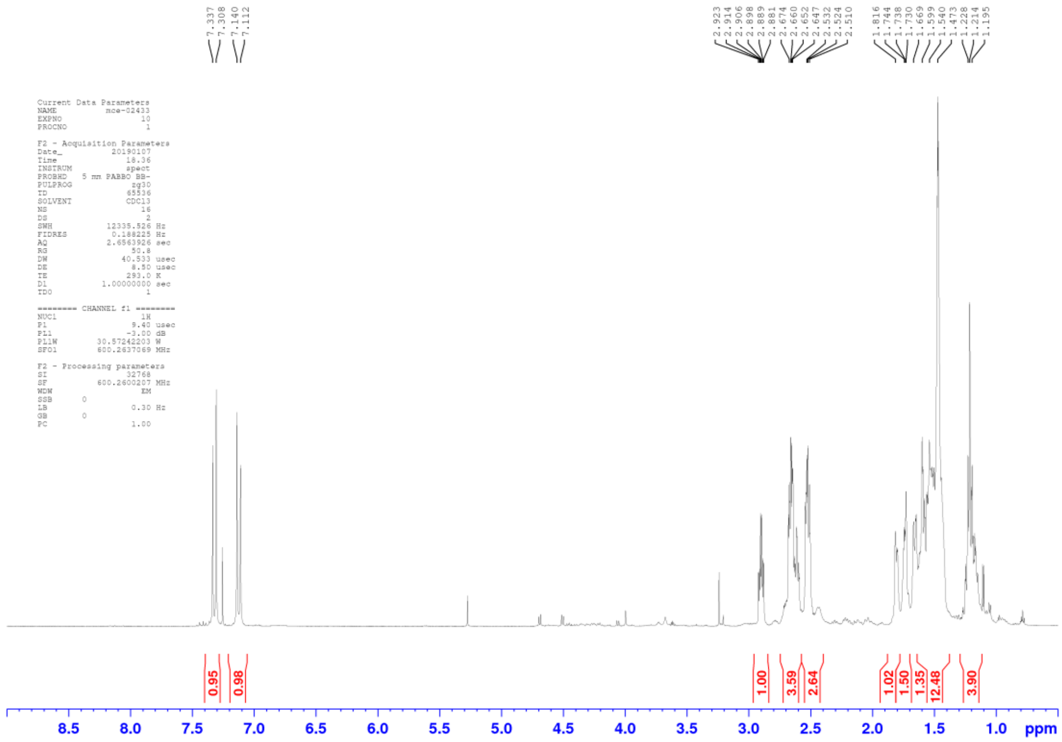


**Figure S11**. $^{1}$H NMR of (*R*,*R*)-**4c** ($CDCl_3$, 600 MHz).

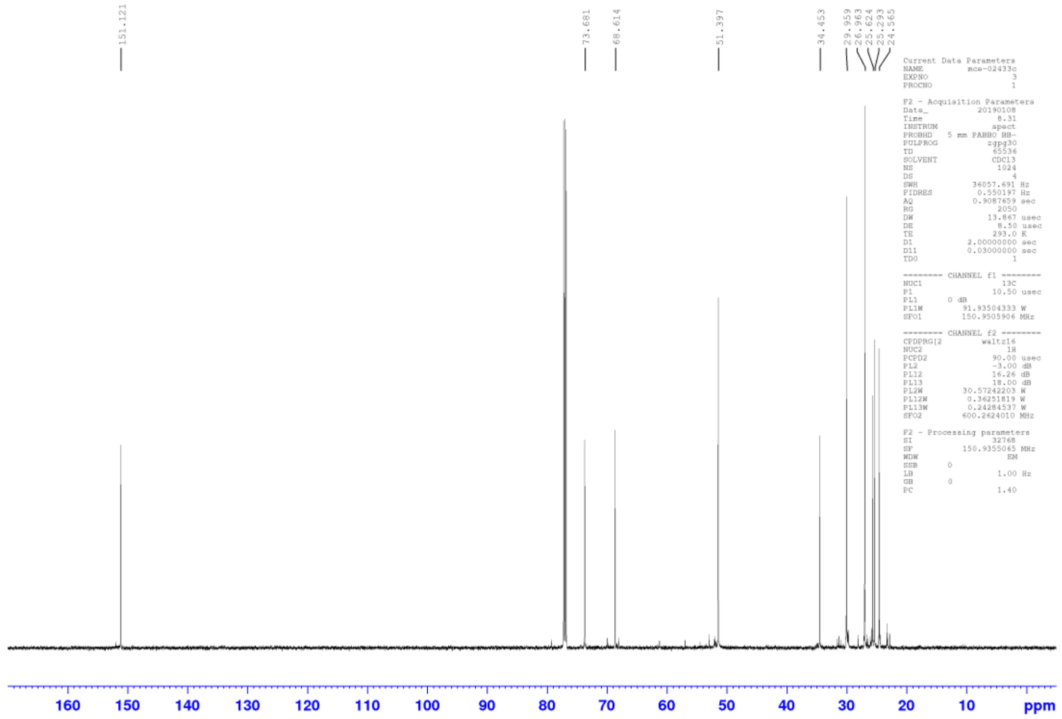


**Figure S12**. $^{13}$C NMR of (*R*,*R*)-**4c** ($CDCl_3$, 151 MHz).

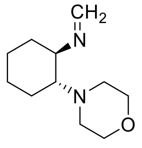


(*R*,*R*)-**4d**

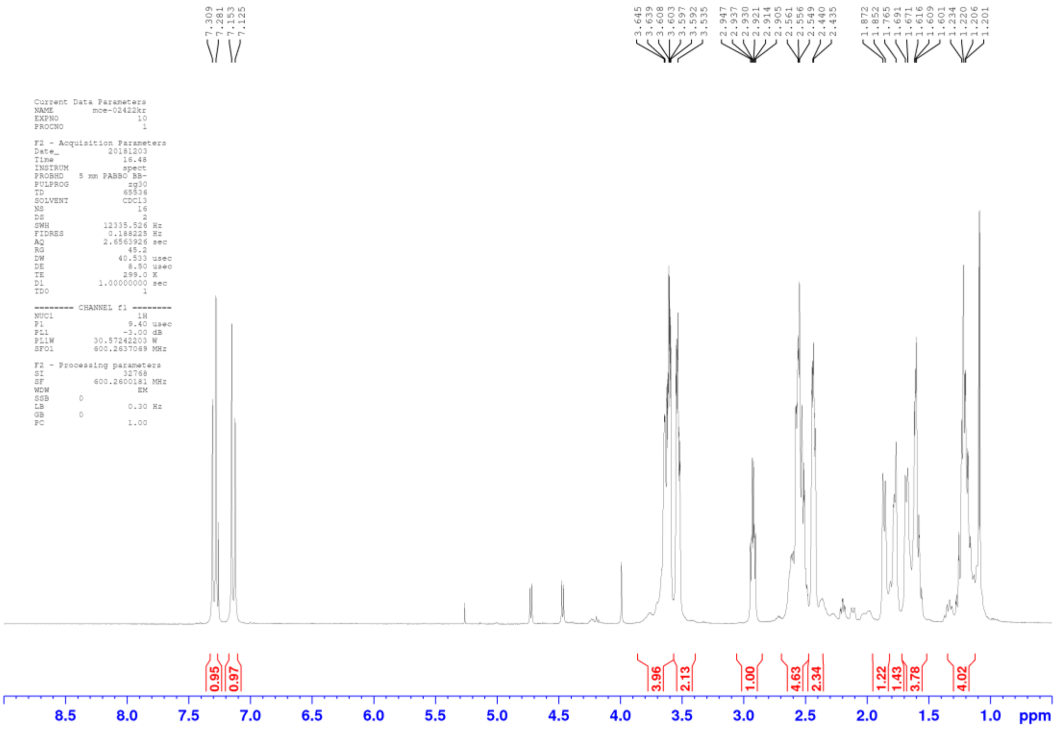


**Figure S13**. $^{1}$H NMR of (*R*,*R*)-**4d** ($CDCl_3$, 600 MHz).

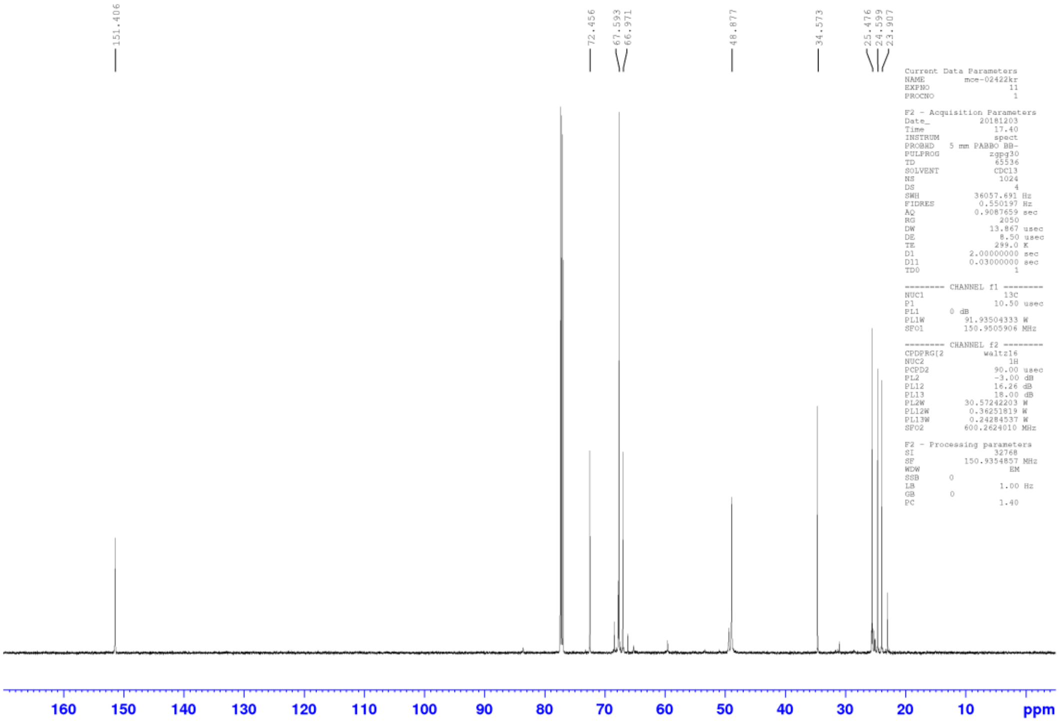


**Figure S14**. $^{13}$C NMR of (*R*,*R*)-**4d** ($CDCl_3$, 151 MHz).

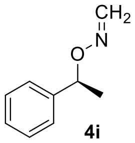


**4i**

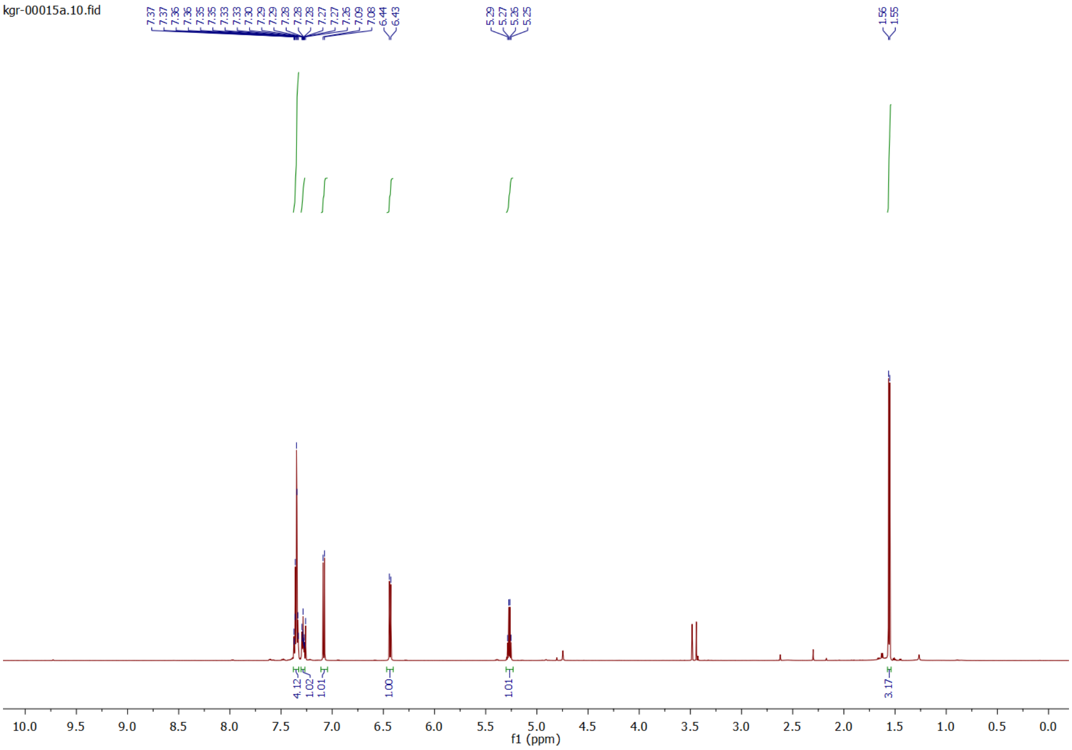


**Figure S15**. $^{1}$H NMR of **4i** ($CDCl_3$, 600 MHz).

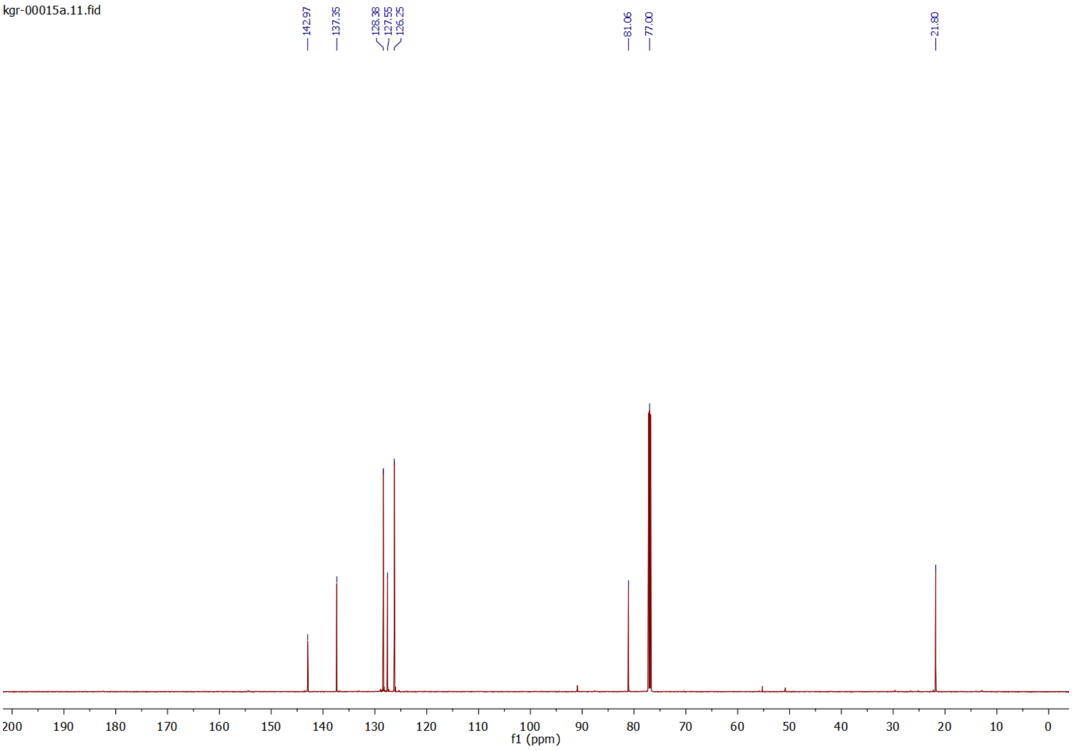


**Figure S16**. $^{13}$C NMR of **4i** ($CDCl_3$, 151 MHz).

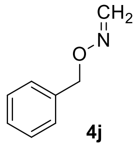


**4j**

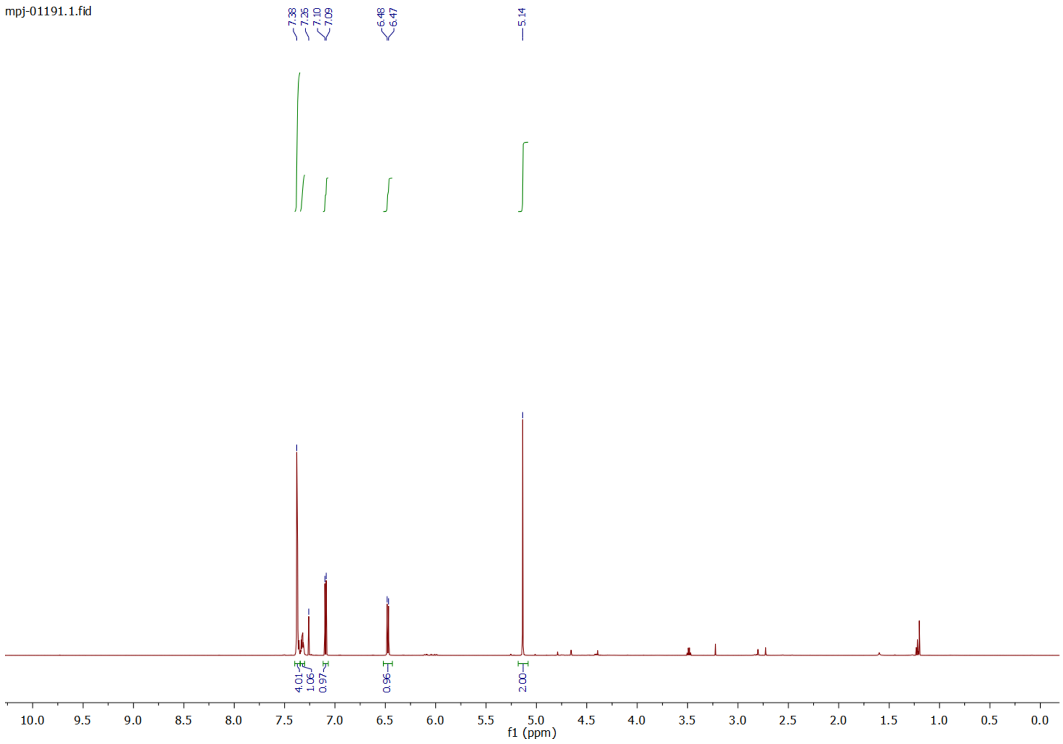


**Figure S17**. $^{1}$H NMR of **4j** ($CDCl_3$, 600 MHz).

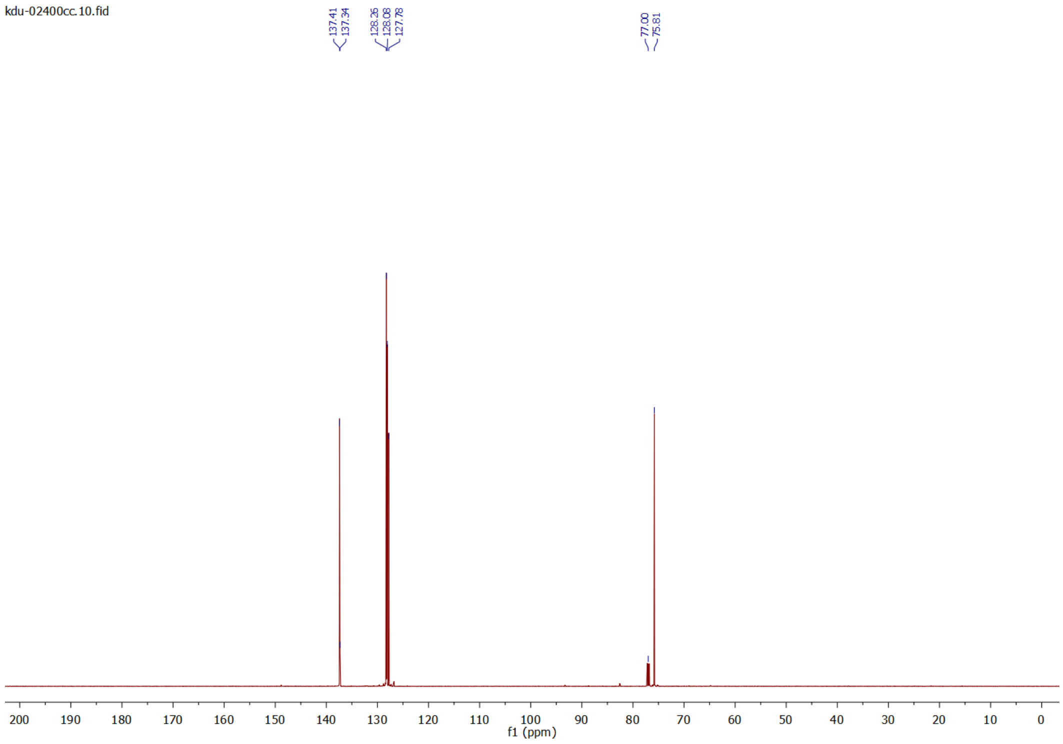


**Figure S18**. $^{13}$C NMR of **4j** ($CDCl_3$, 151 MHz).

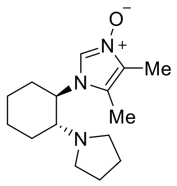


(*R*,*R*)-**6a**

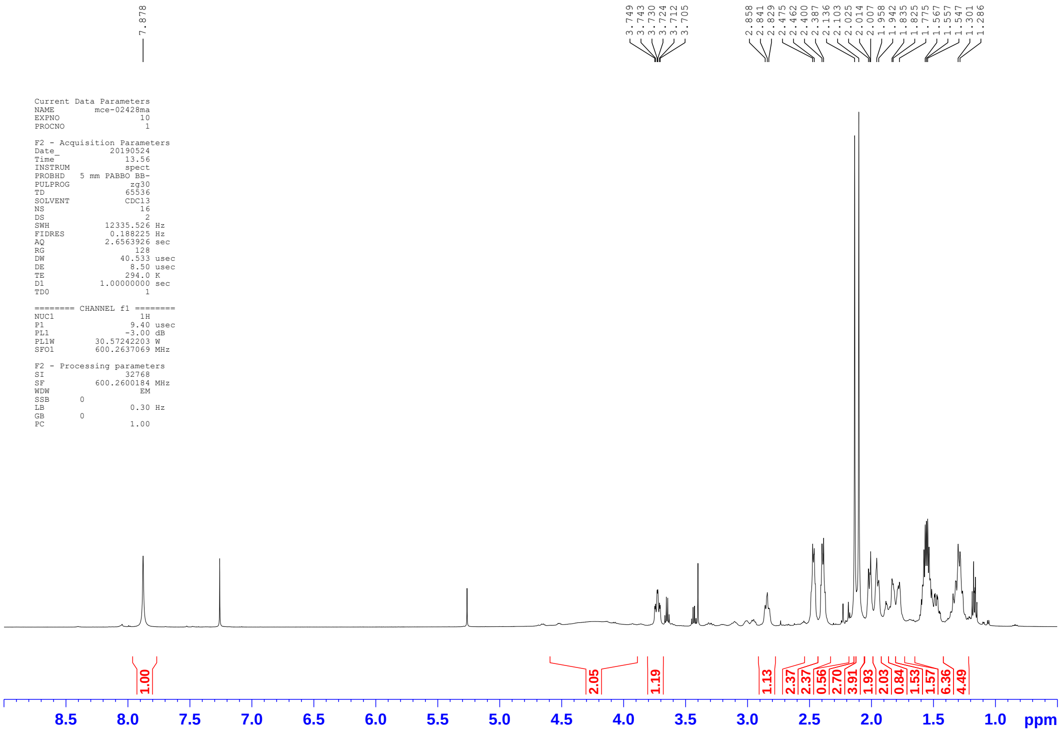


**Figure S19**. $^{1}$H NMR of (*R*,*R*)-**6a** ($CDCl_3$, 600 MHz).

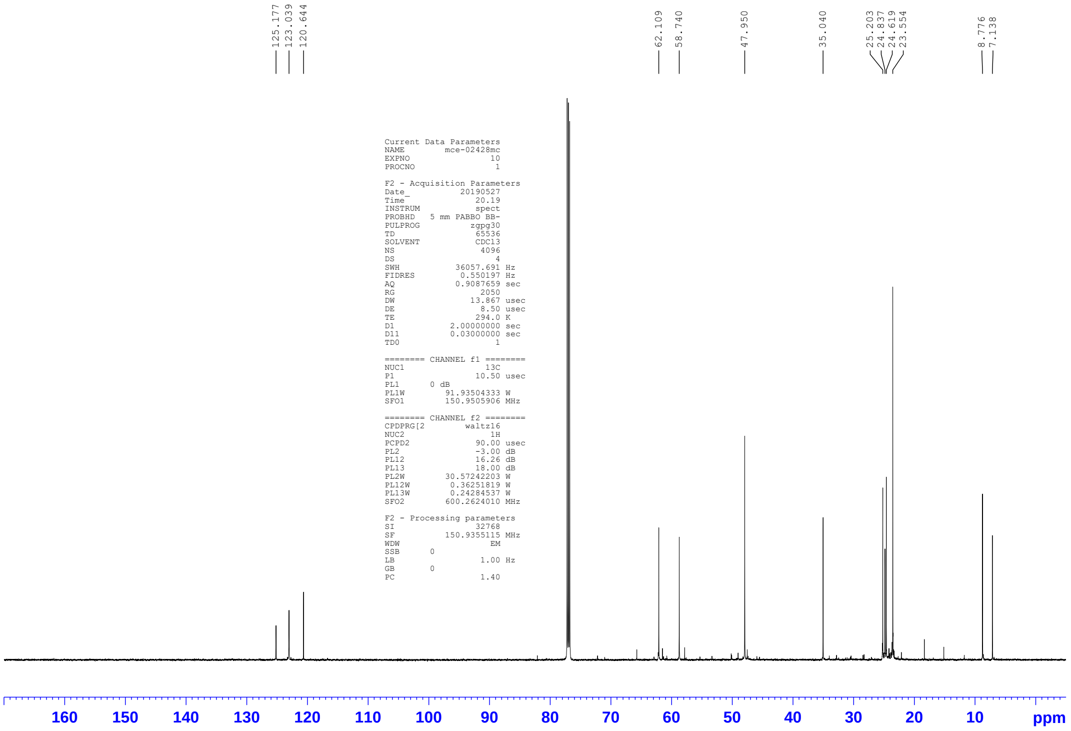


**Figure S20**. $^{13}$C NMR of (*R*,*R*)-**6a** ($CDCl_3$, 151 MHz).

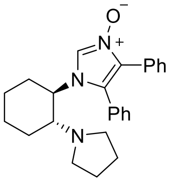

(*R*,*R*)-**6b**

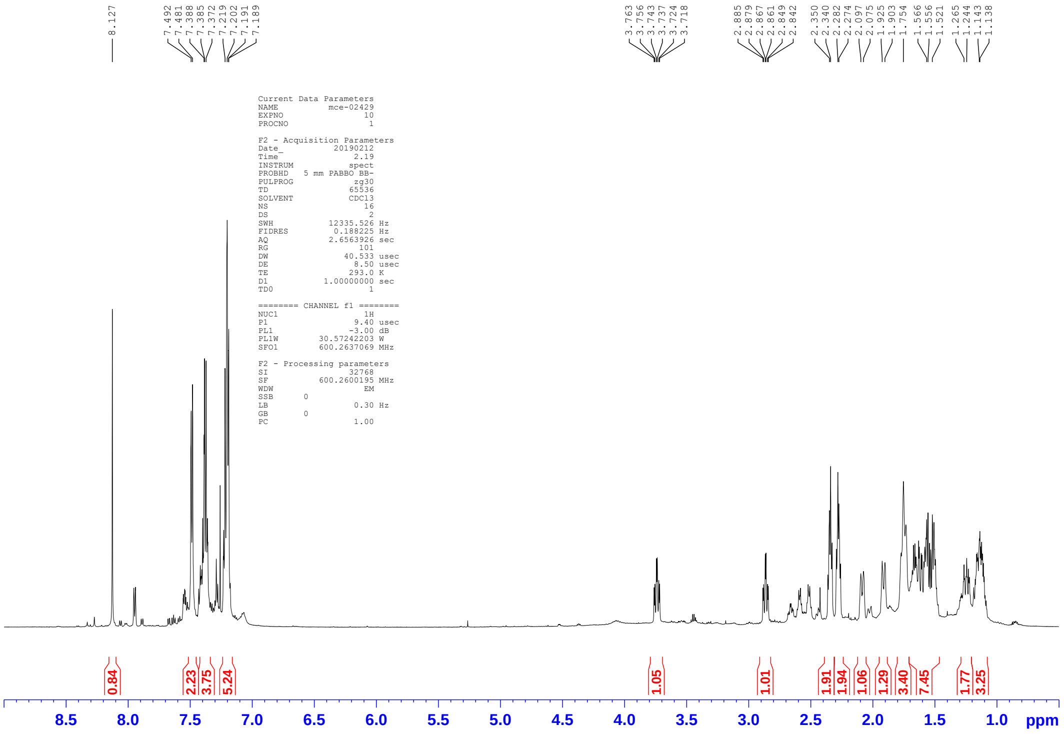

**Figure S21**. $^{1}$H NMR of (*R*,*R*)-**6b** ($CDCl_3$, 600 MHz).

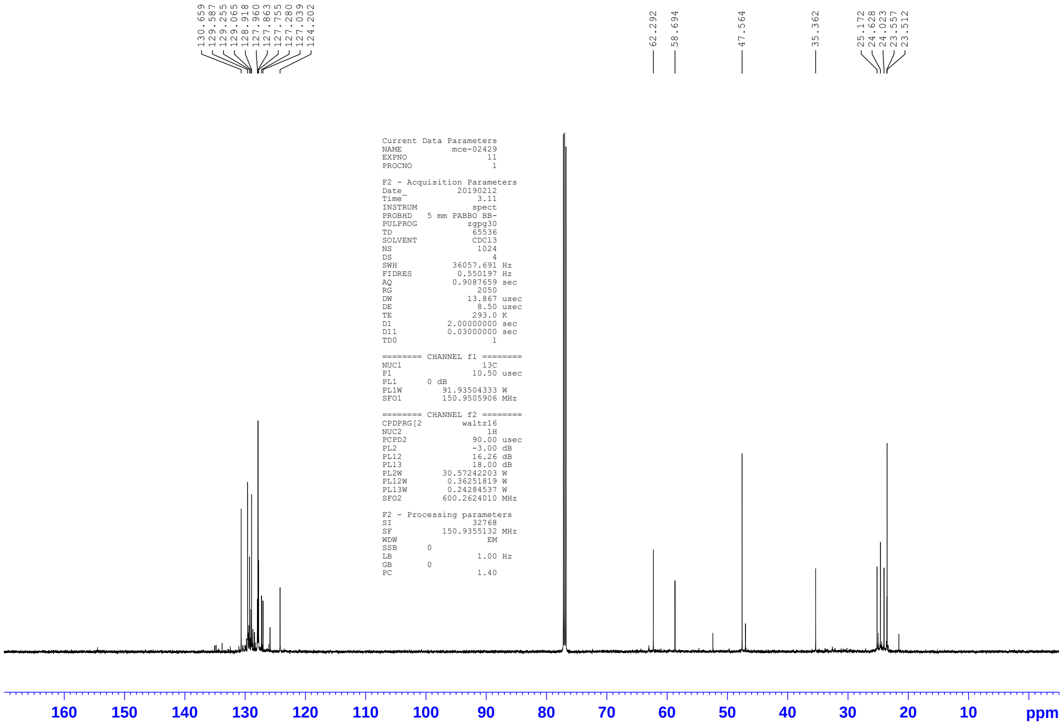

**Figure S22**. $^{13}$C NMR of (*R*,*R*)-**6b** ($CDCl_3$, 151 MHz).

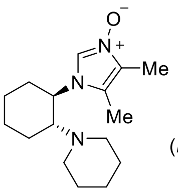

(*R*,*R*)-**6c**

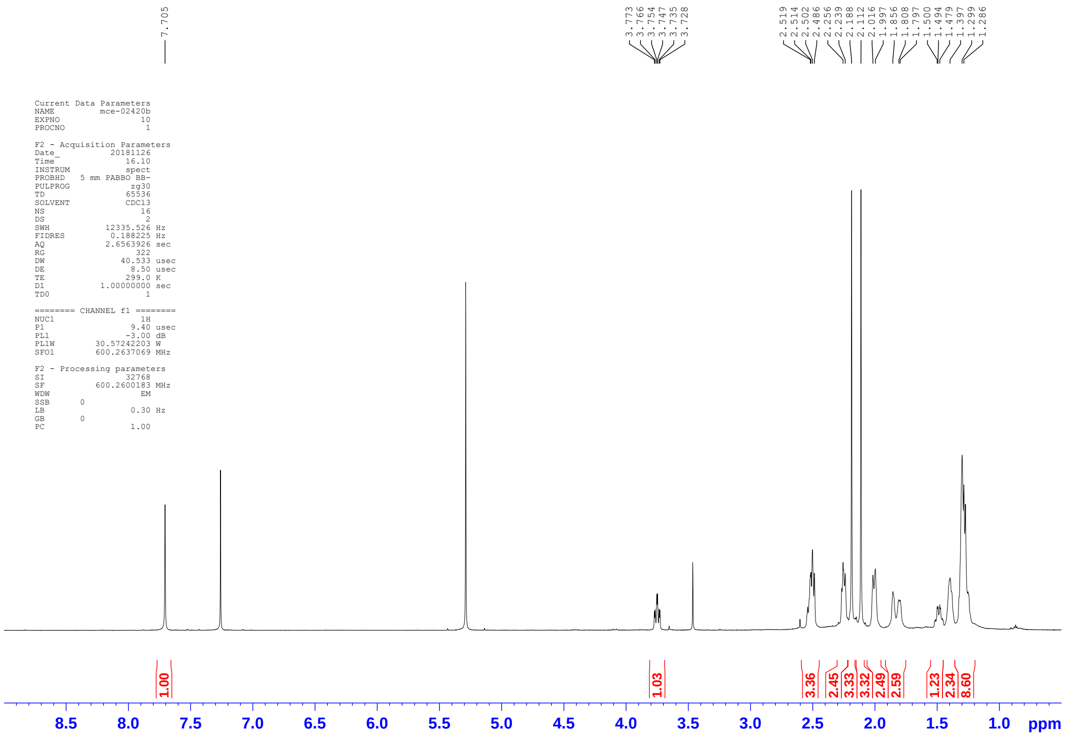


**Figure S23**. $^{1}H$ NMR of (*R*,*R*)-**6c** ($CDCl_3$, 600 MHz).

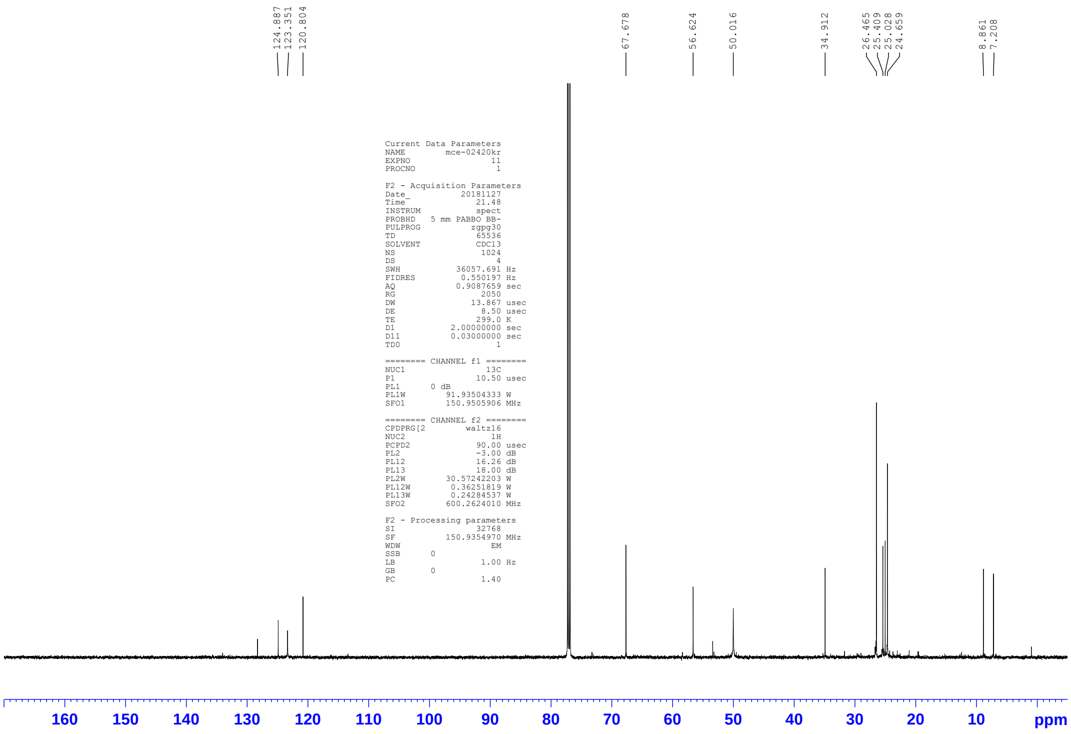


**Figure S24**. $^{13}C$ NMR of (*R*,*R*)-**6c** ($CDCl_3$, 151 MHz).

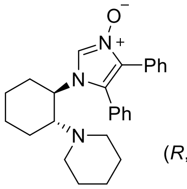


(*R*,*R*)-**6d**

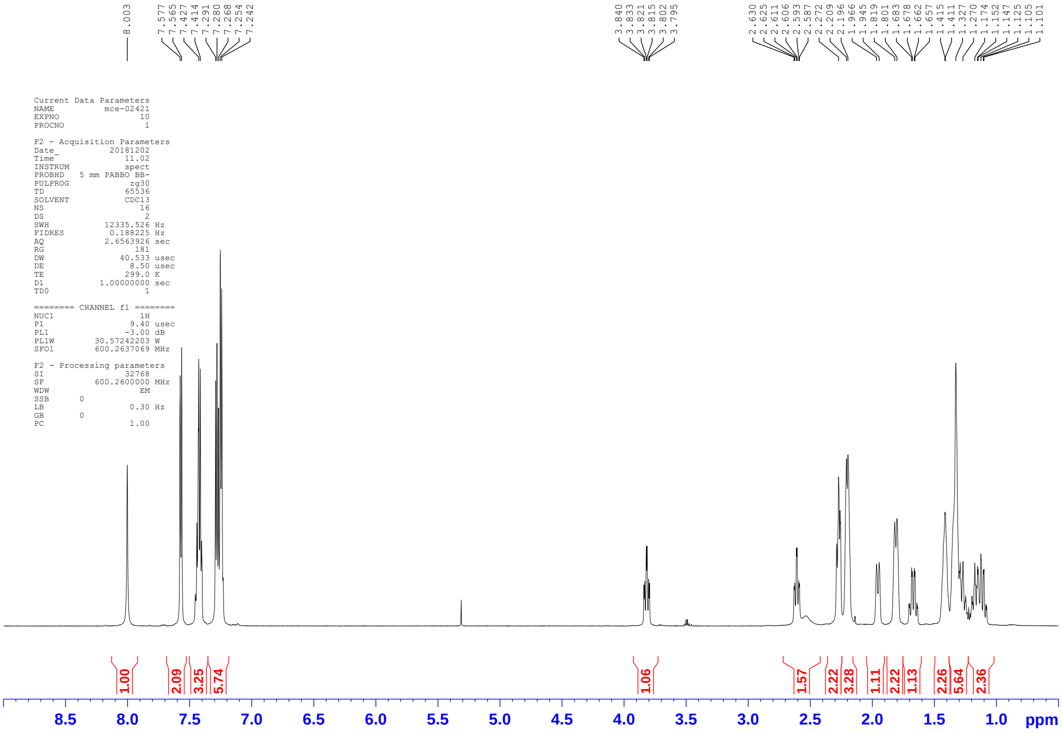


**Figure S25**. $^{1}H$ NMR of (*R*,*R*)-**6d** ($CDCl_3$, 600 MHz).

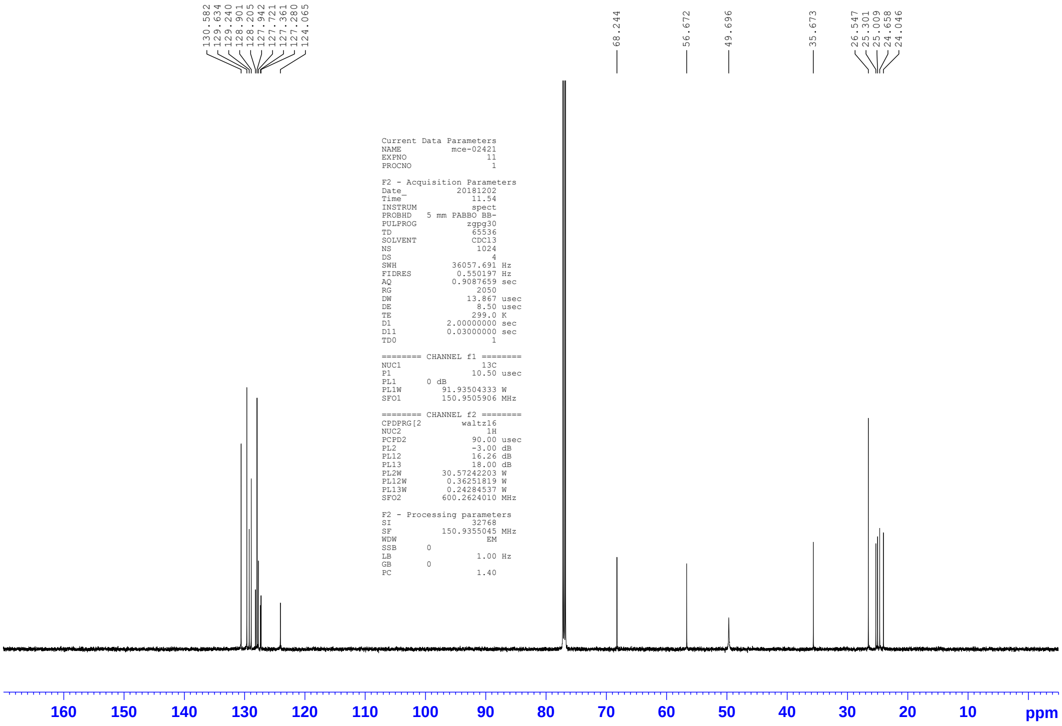


**Figure S26**. $^{13}C$ NMR of (*R*,*R*)-**6d** ($CDCl_3$, 151 MHz).

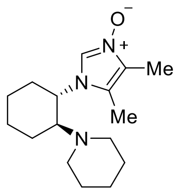


(*S*,*S*)-**6c**

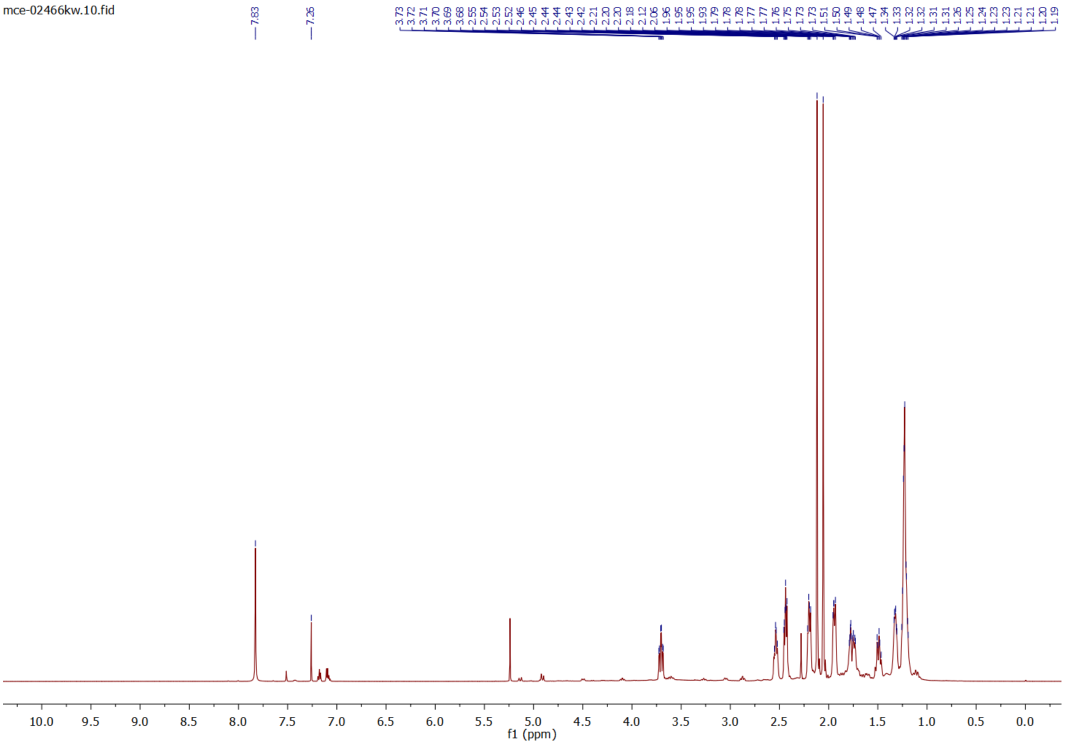


**Figure S27**. $^{1}$H NMR of (*S*,*S*)-**6c** ($CDCl_3$, 600 MHz).

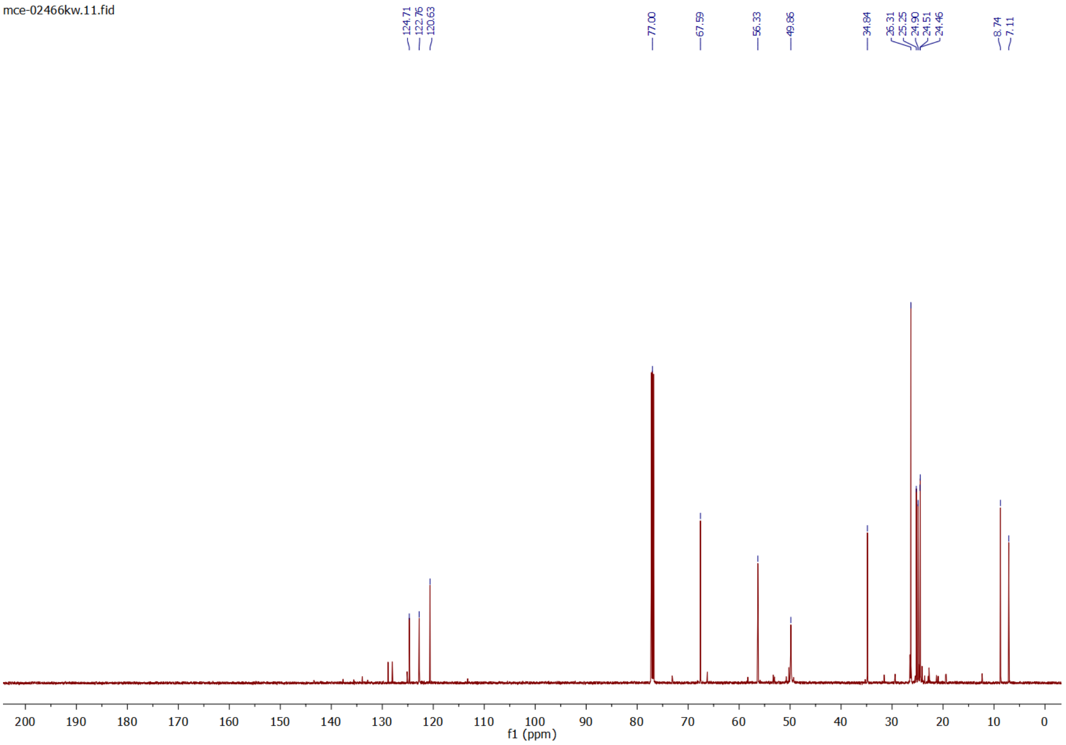


**Figure S28**. $^{13}$C NMR of (*S*,*S*)-**6c** ($CDCl_3$, 151 MHz).

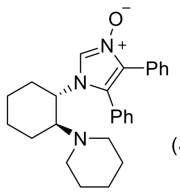

(*S*,*S*)-**6d**

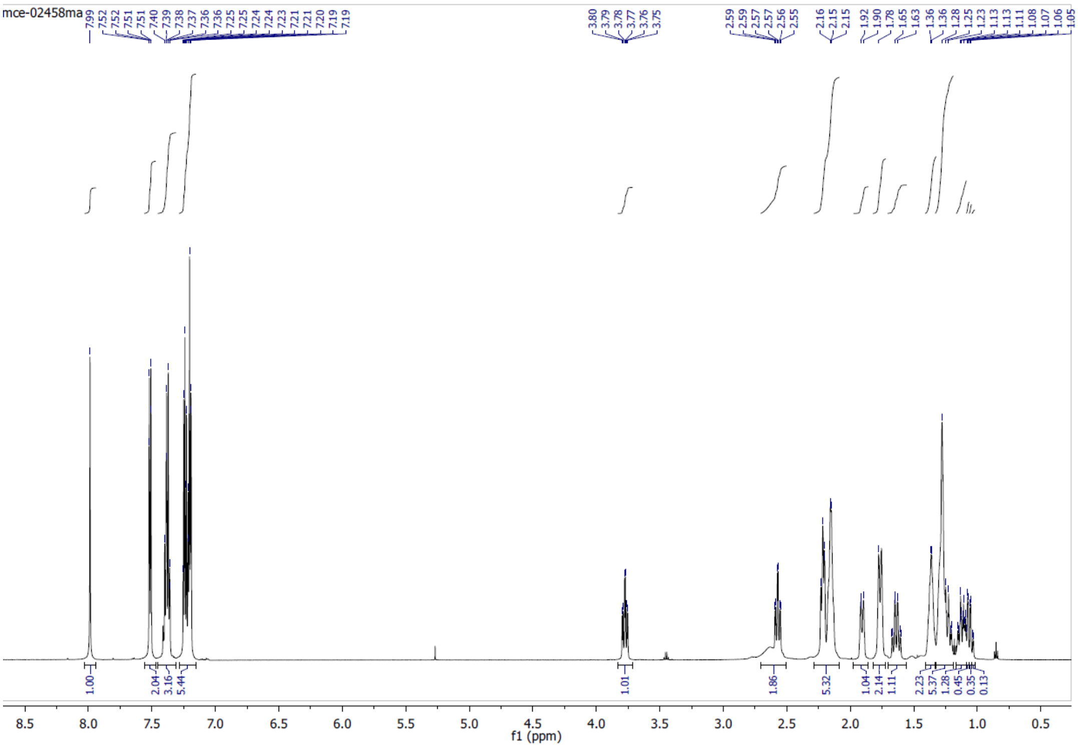

**Figure S29**. $^{1}$H NMR of (*S*,*S*)-**6d** ($CDCl_3$, 600 MHz).

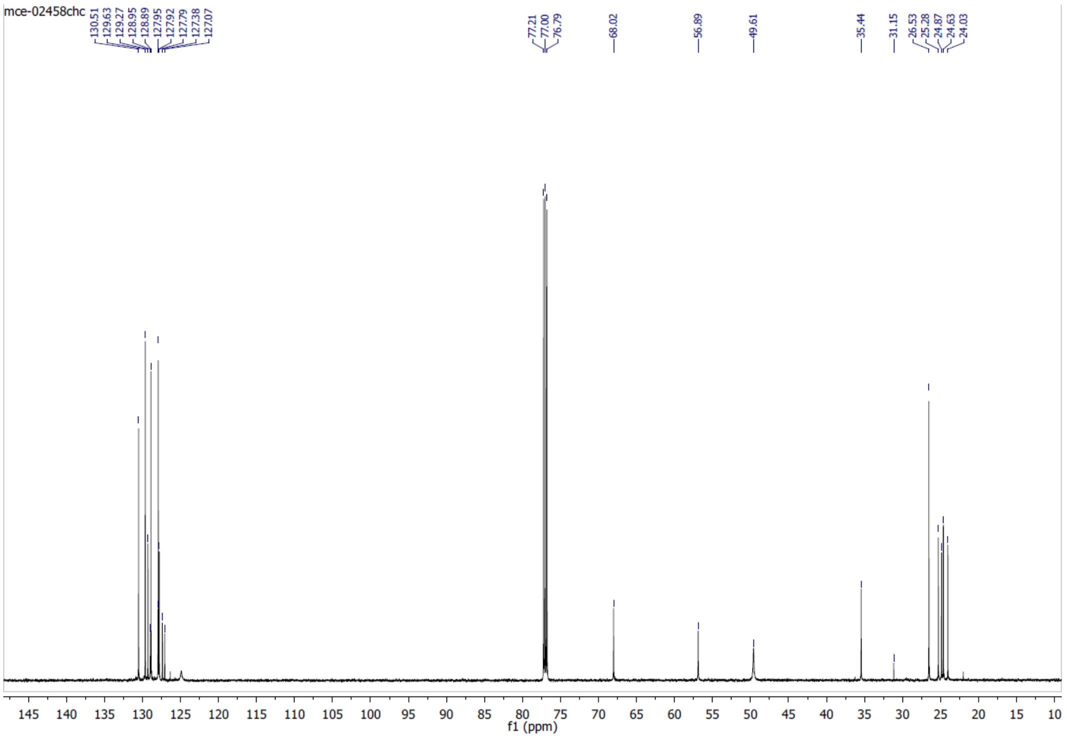

**Figure S30**. $^{13}$C NMR of (*S*,*S*)-**6d** ($CDCl_3$, 151 MHz).

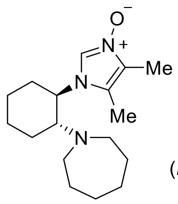


(*R*,*R*)-**6e**

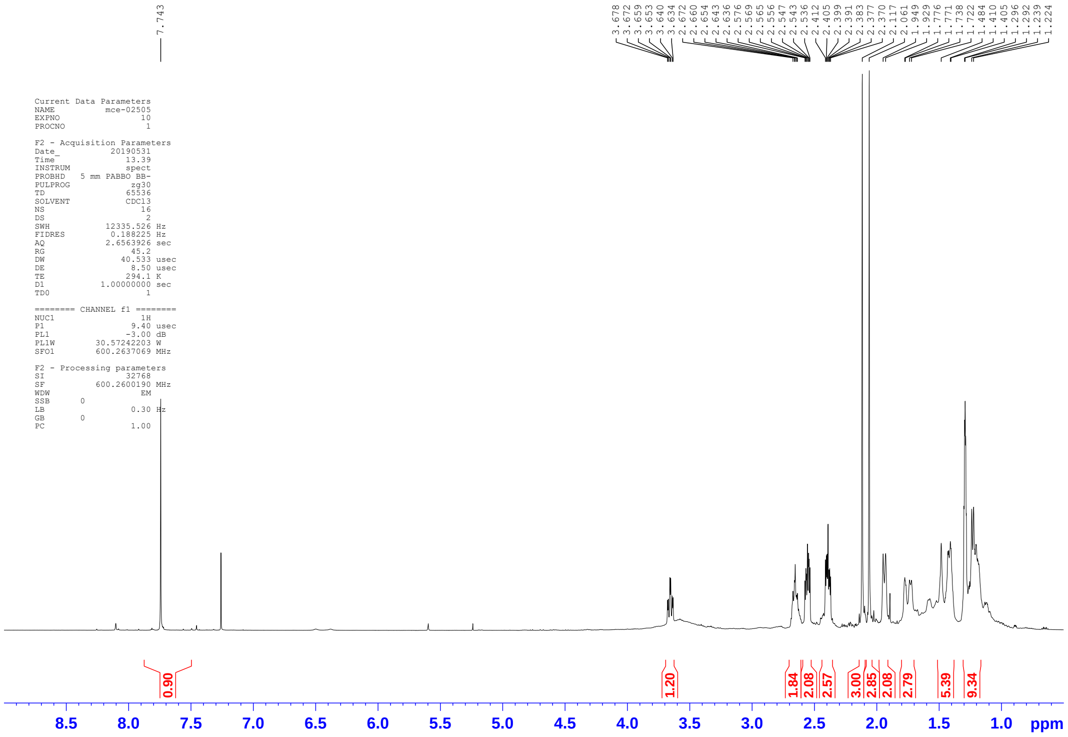


**Figure S31**. $^{1}$H NMR of (*R*,*R*)-**6e** ($CDCl_3$, 600 MHz).

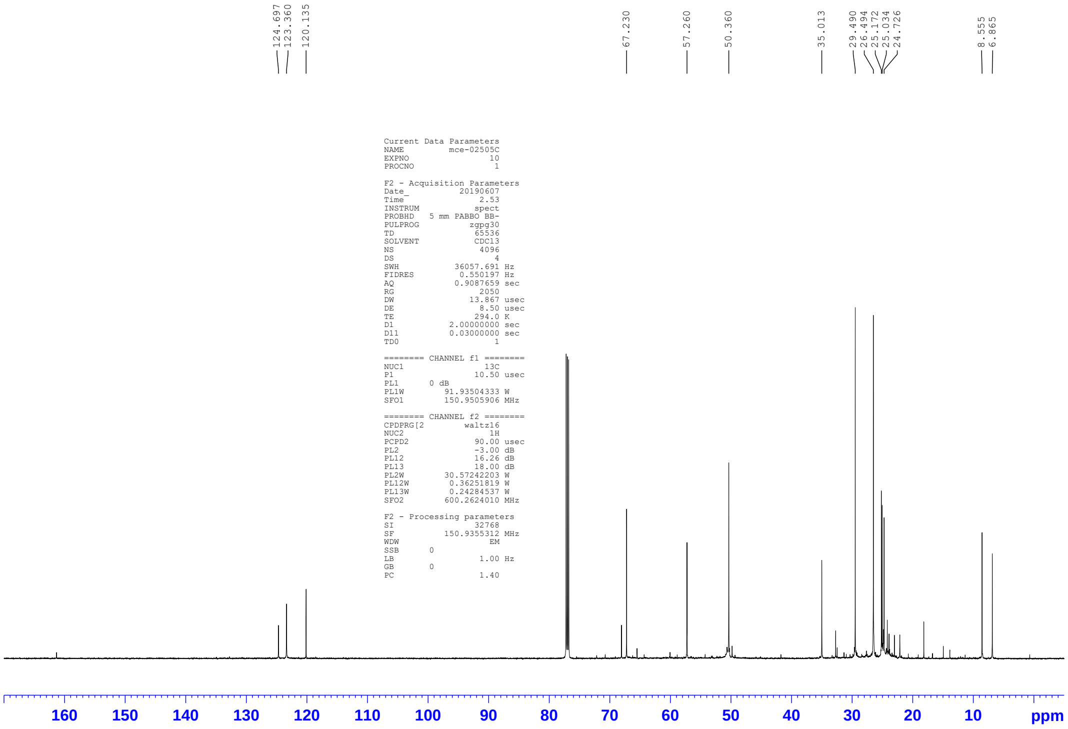


**Figure S32**. $^{13}$C NMR of (*R*,*R*)-**6e** ($CDCl_3$, 151 MHz).

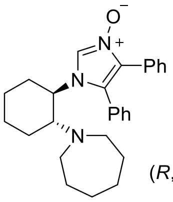


(*R*,*R*)-**6f**

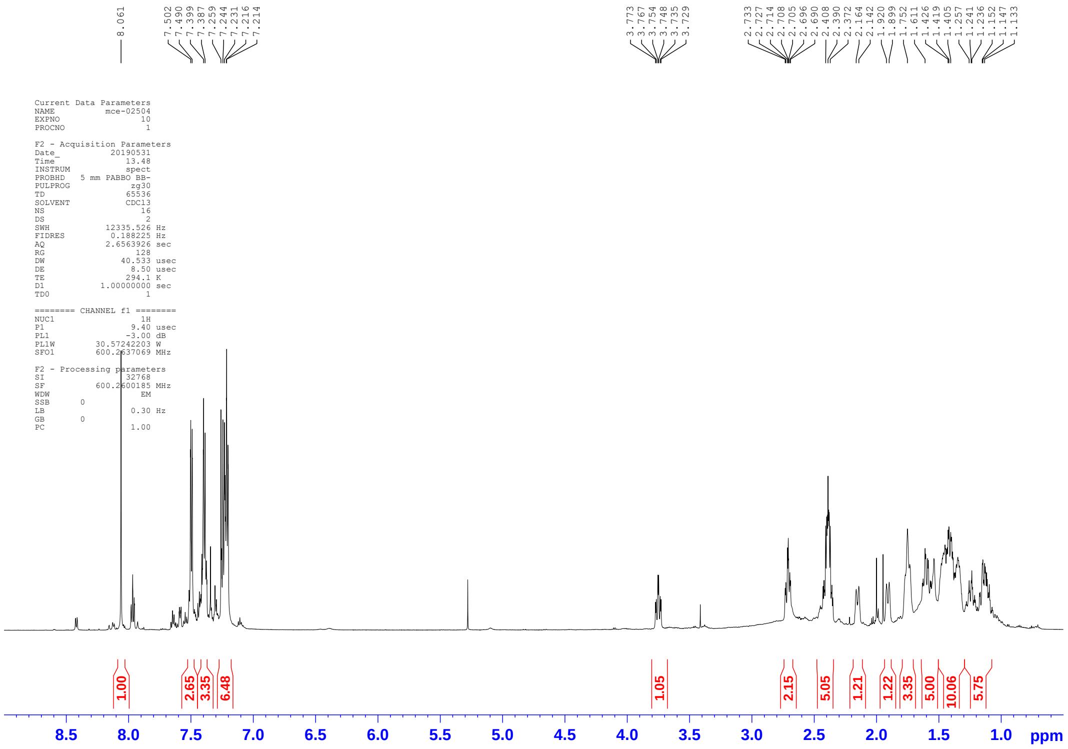


**Figure S33**. ¹H NMR of (*R*,*R*)-**6f** ($CDCl_3$, 600 MHz).

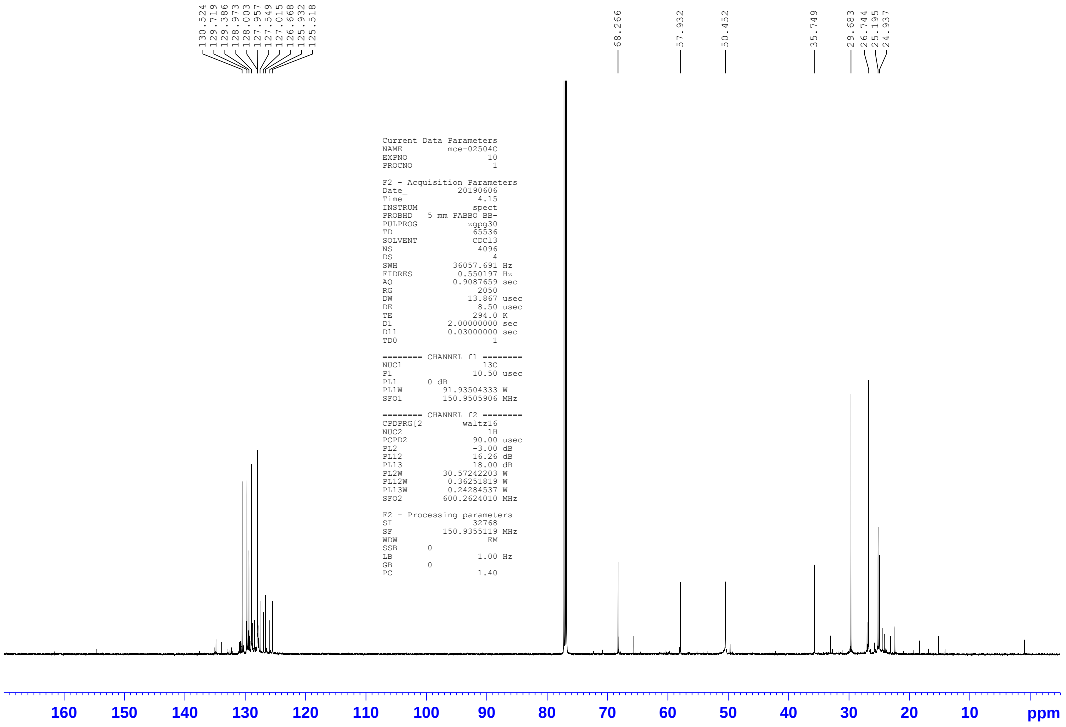


**Figure S34**. ¹³C NMR of (*R*,*R*)-**6f** ($CDCl_3$, 151 MHz).

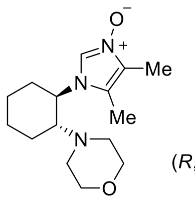


(*R*,*R*)-**6g**

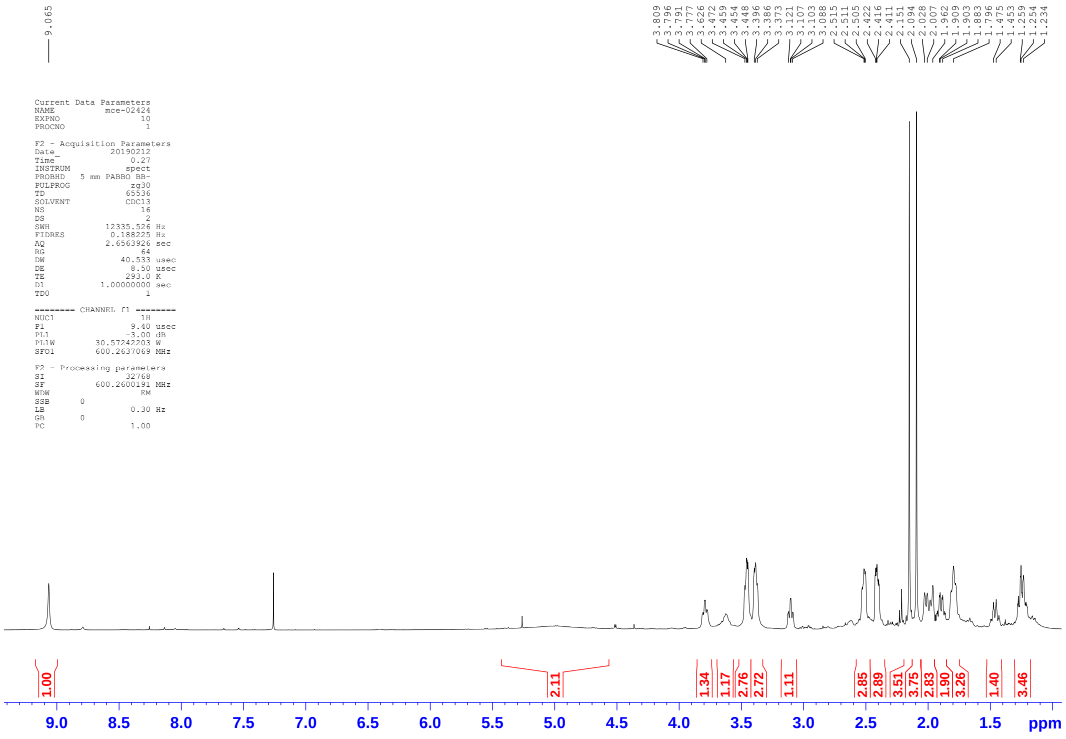


**Figure S35**. $^{1}H$ NMR of (*R*,*R*)-**6g** ($CDCl_3$, 600 MHz).

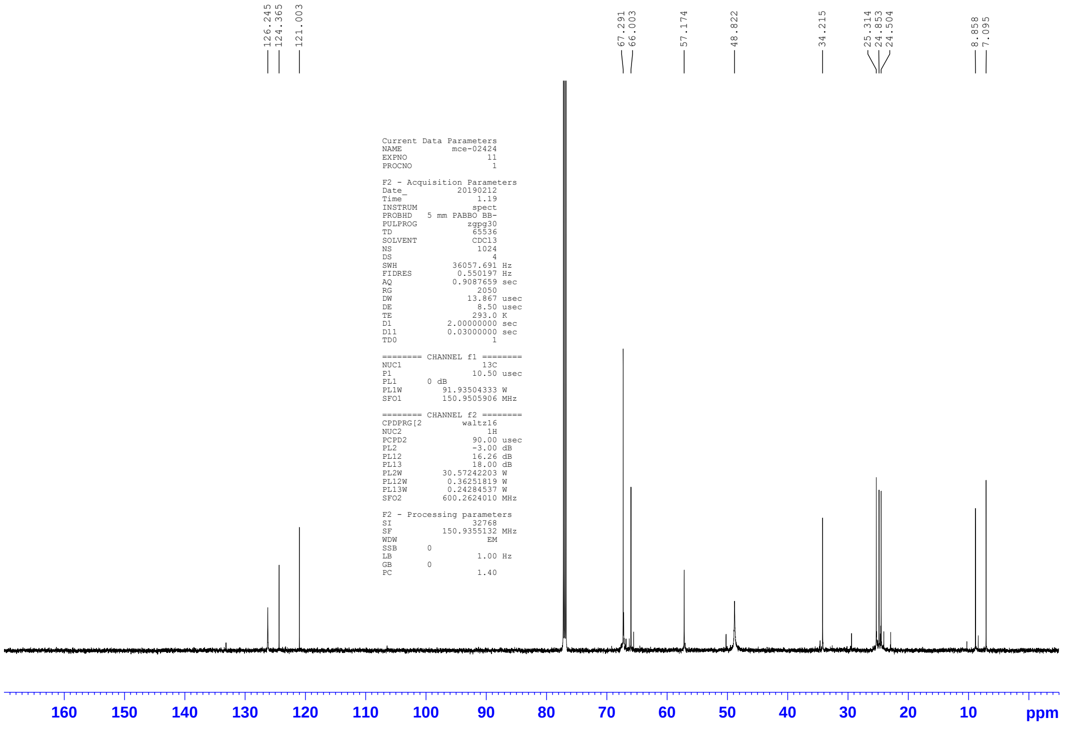


**Figure S36**. $^{13}C$ NMR of (*R*,*R*)-**6g** ($CDCl_3$, 151 MHz).

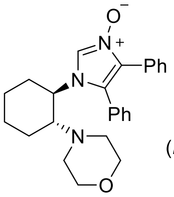


(*R*,*R*)-**6h**

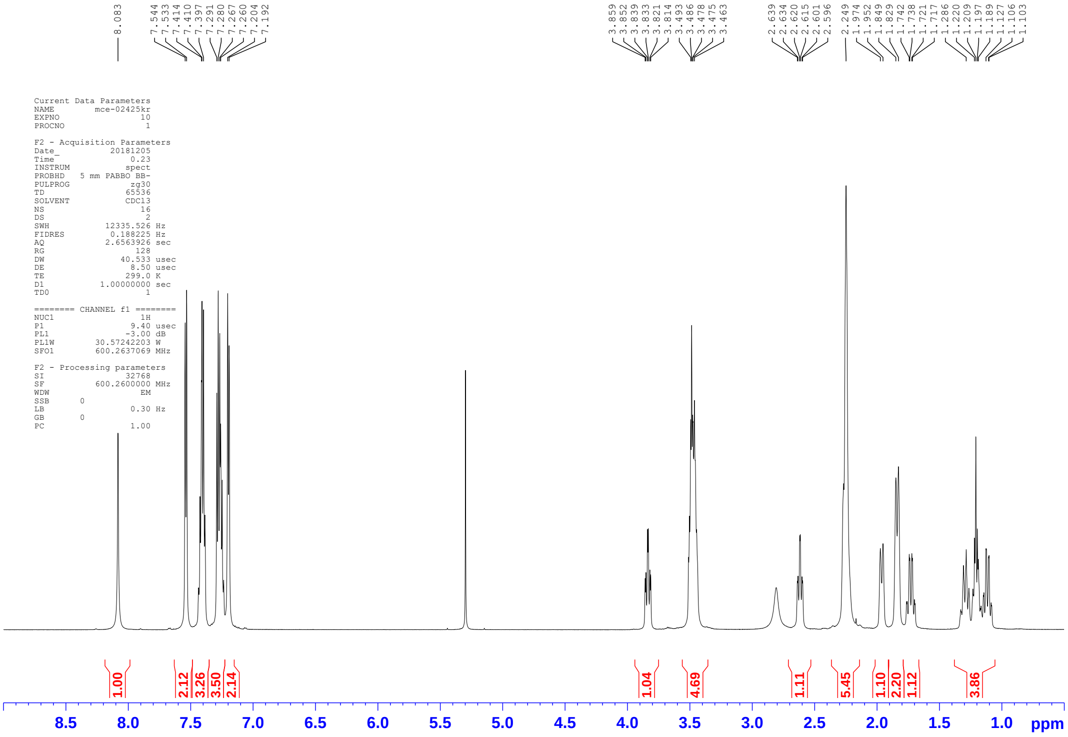


**Figure S37**. $^{1}$H NMR of (*R*,*R*)-**6h** ($CDCl_3$, 600 MHz).

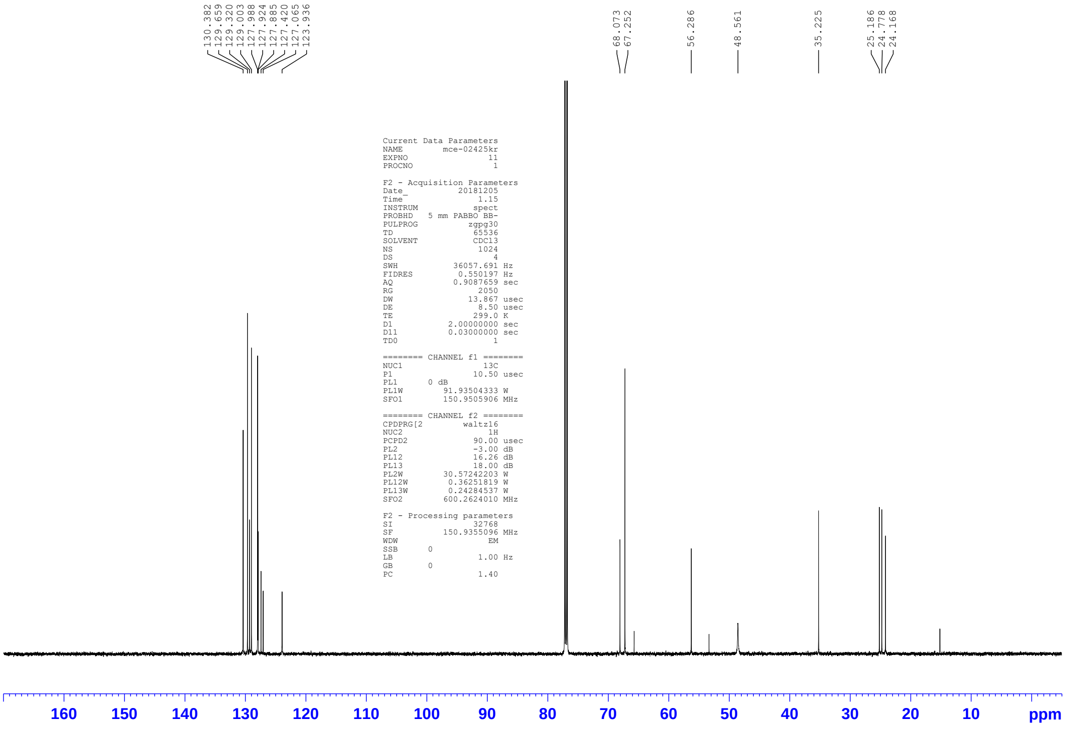


**Figure S38**. $^{13}$C NMR of (*R*,*R*)-**6h** ($CDCl_3$, 151 MHz).

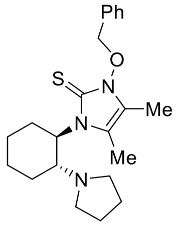


(*R*,*R*)-**9a**

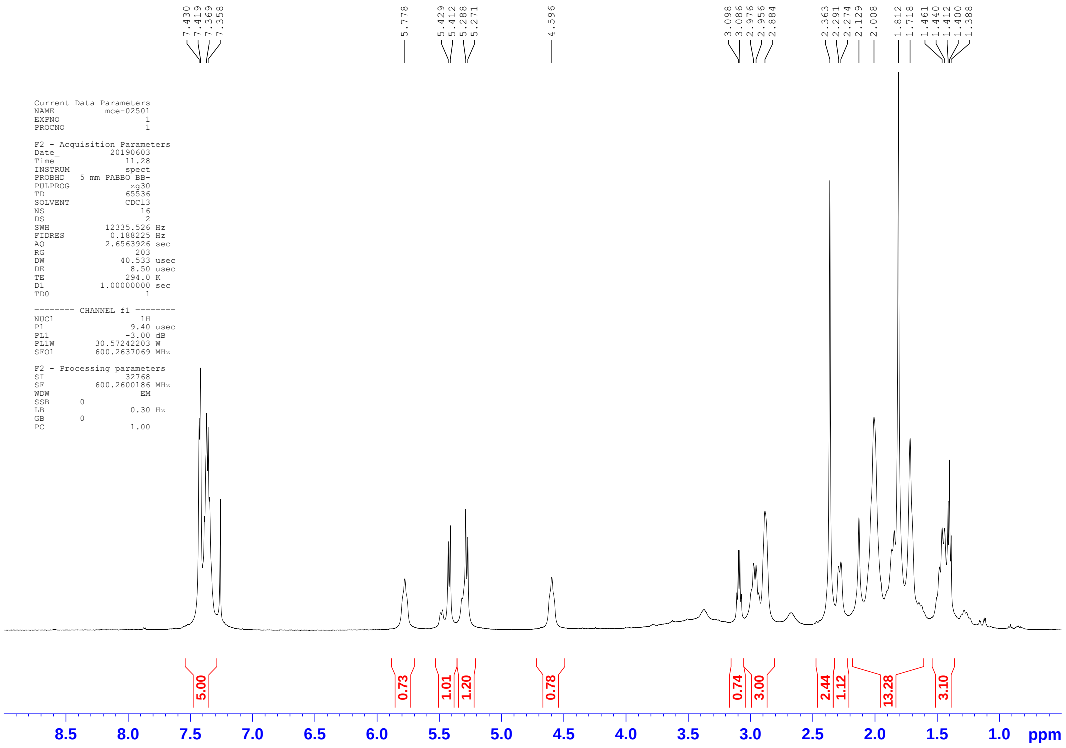


**Figure S39**. $^{1}$H NMR of (*R*,*R*)-**9a** ($CDCl_3$, 600 MHz).

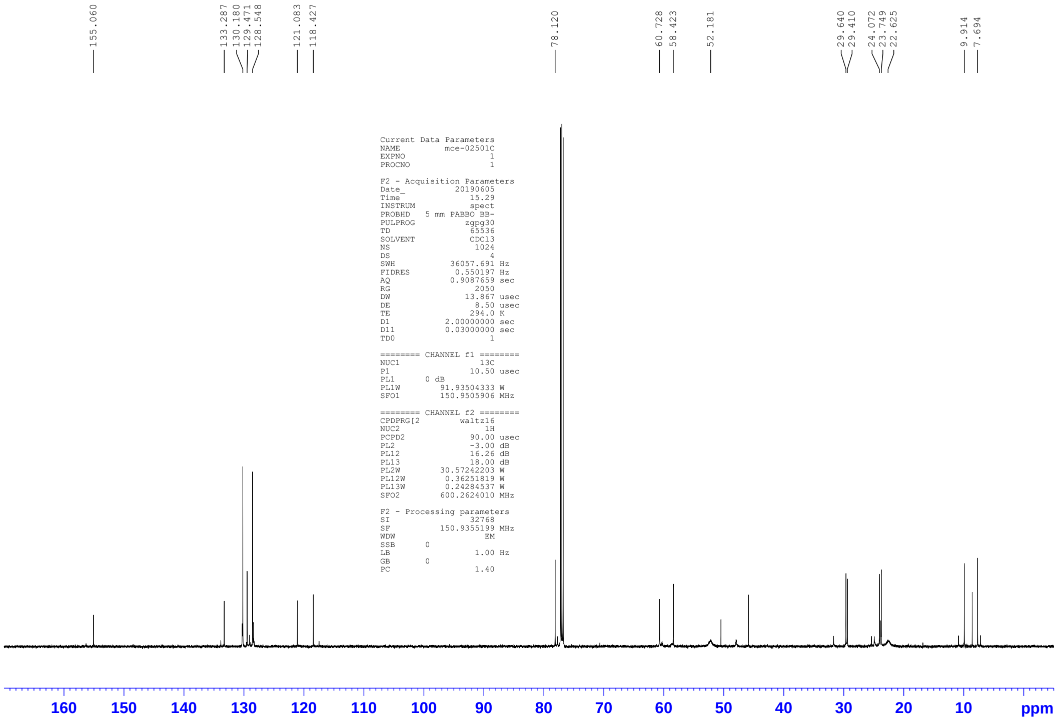


**Figure S40**. $^{13}$C NMR of (*R*,*R*)-**9a** ($CDCl_3$, 151 MHz).

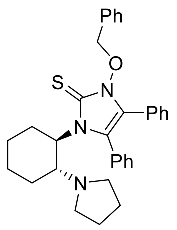


(*R*,*R*)-**9b**

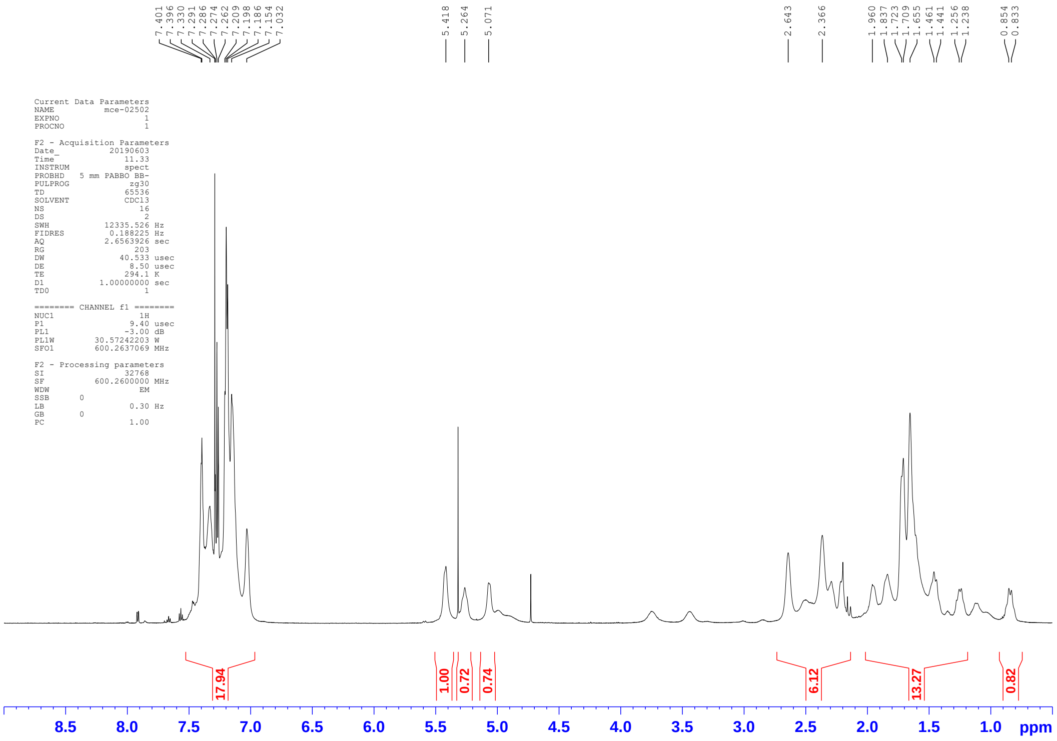


**Figure S41**. $^{1}$H NMR of (*R*,*R*)-**9b** ($CDCl_3$, 600 MHz).

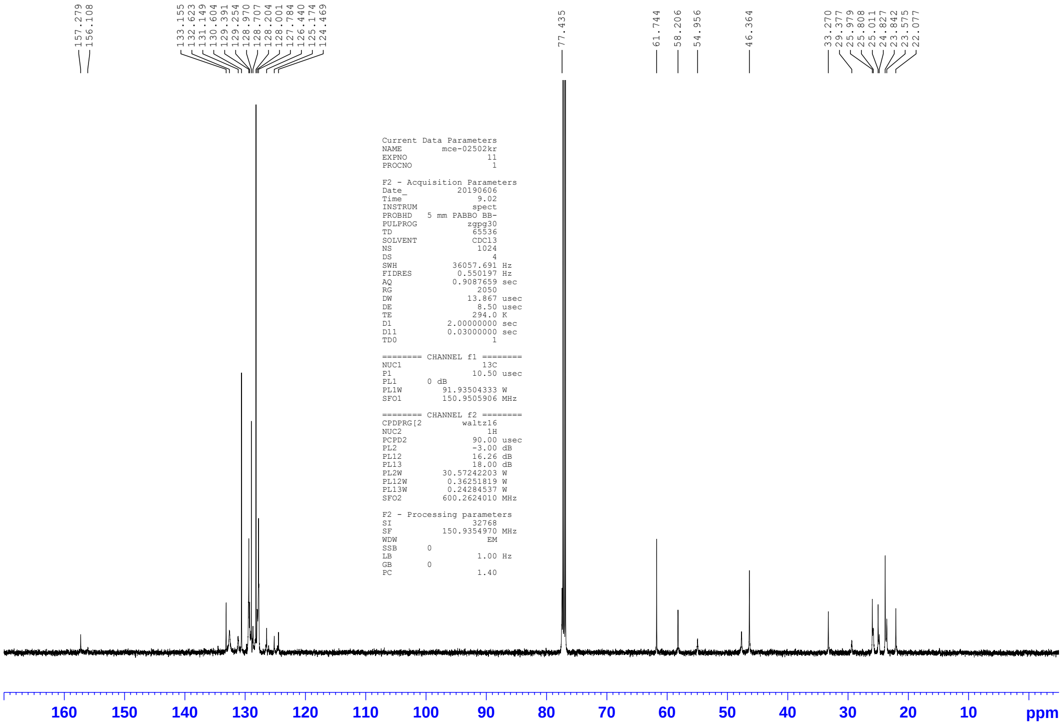


**Figure S42**. $^{13}$C NMR of (*R*,*R*)-**9b** ($CDCl_3$, 151 MHz).

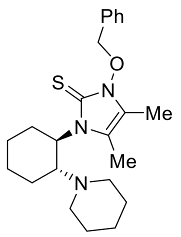


(*R*,*R*)-**9c**

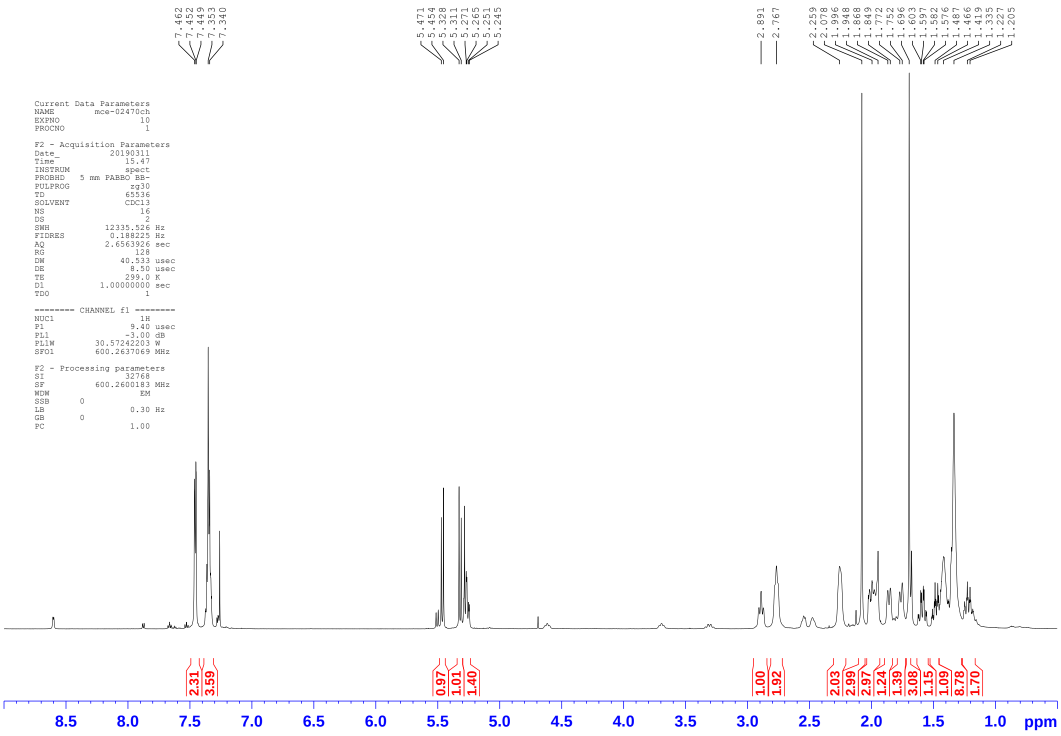


**Figure S43**. $^{1}$H NMR of (*R*,*R*)-**9c** ($CDCl_3$, 600 MHz).

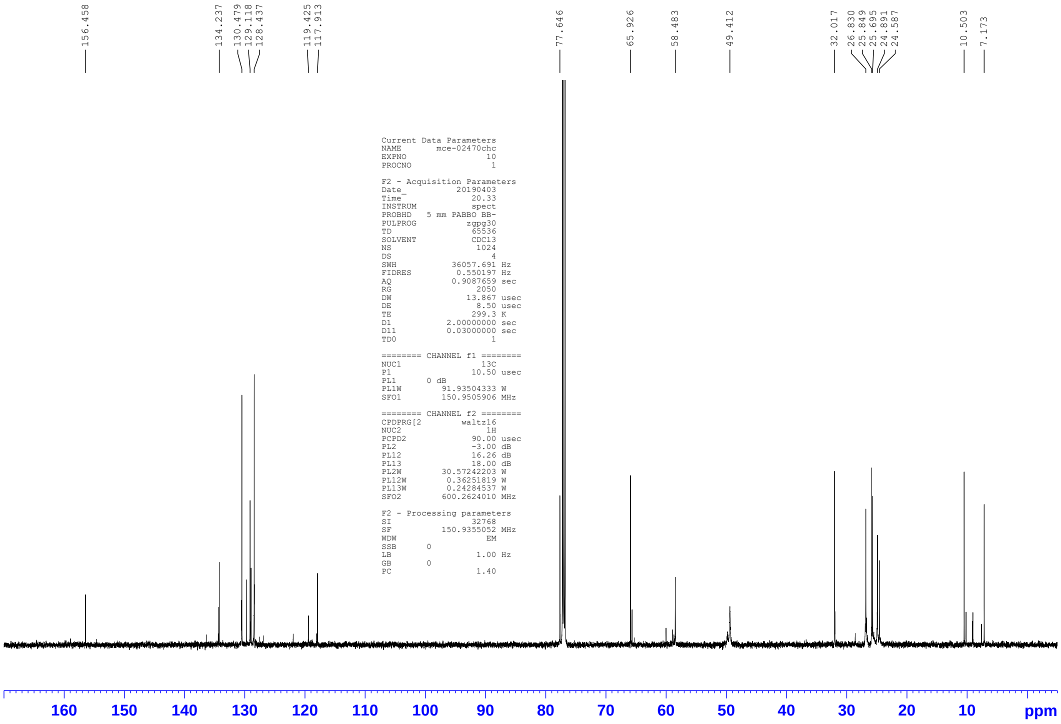


**Figure S44**. $^{13}$C NMR of (*R*,*R*)-**9c** ($CDCl_3$, 151 MHz).

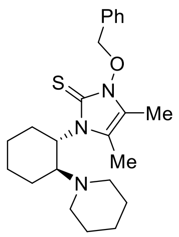


(*S*,*S*)-**9c**

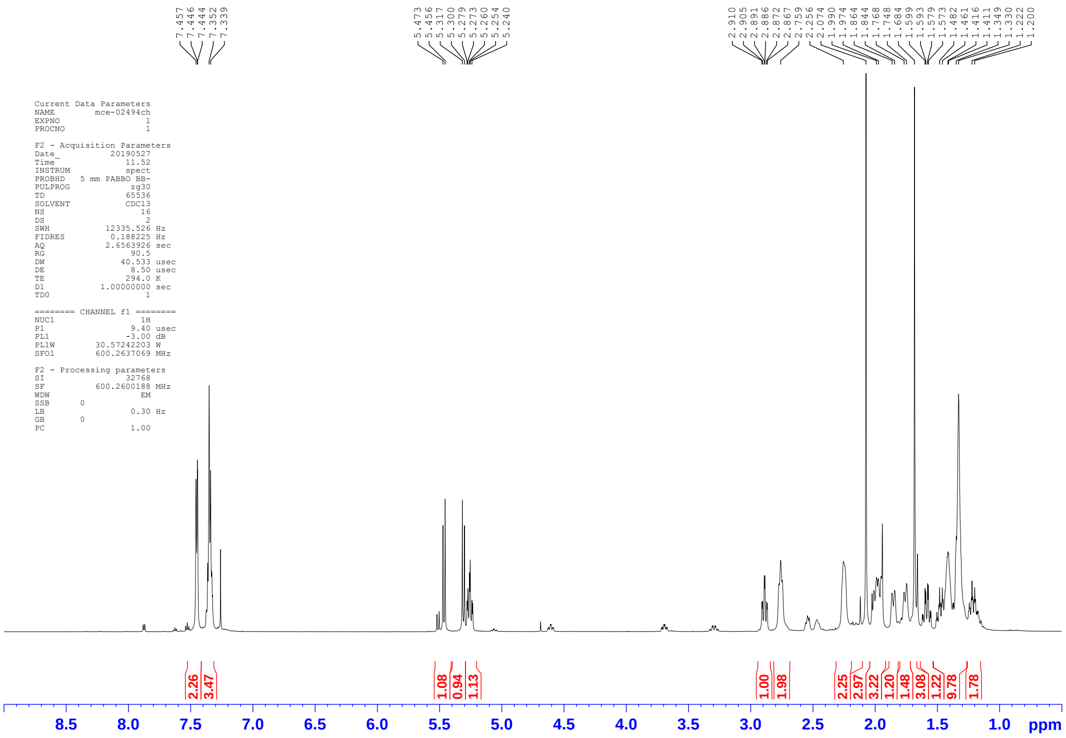


**Figure S45**. $^{1}$H NMR of (*S*,*S*)-**9c** ($CDCl_3$, 600 MHz).

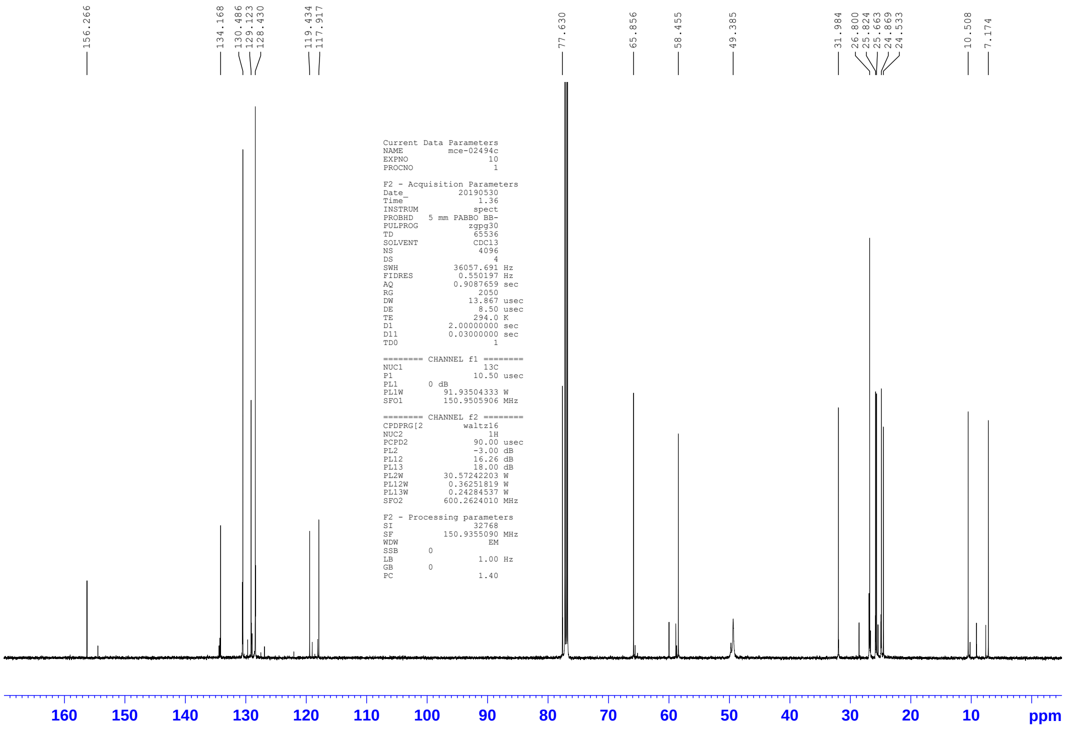


**Figure S46**. $^{13}$C NMR of (*S*,*S*)-**9c** ($CDCl_3$, 151 MHz).

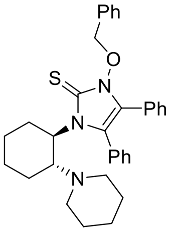


(*R*,*R*)-**9d**

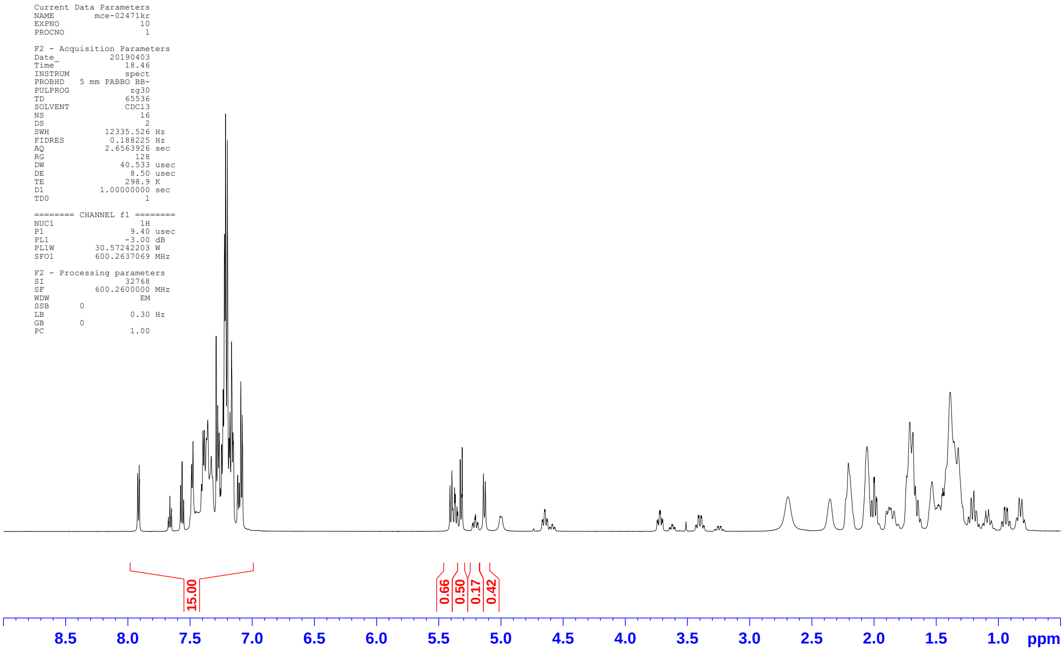


**Figure S47**. $^{1}$H NMR of (*R*,*R*)-**9d** ($CDCl_3$, 600 MHz).

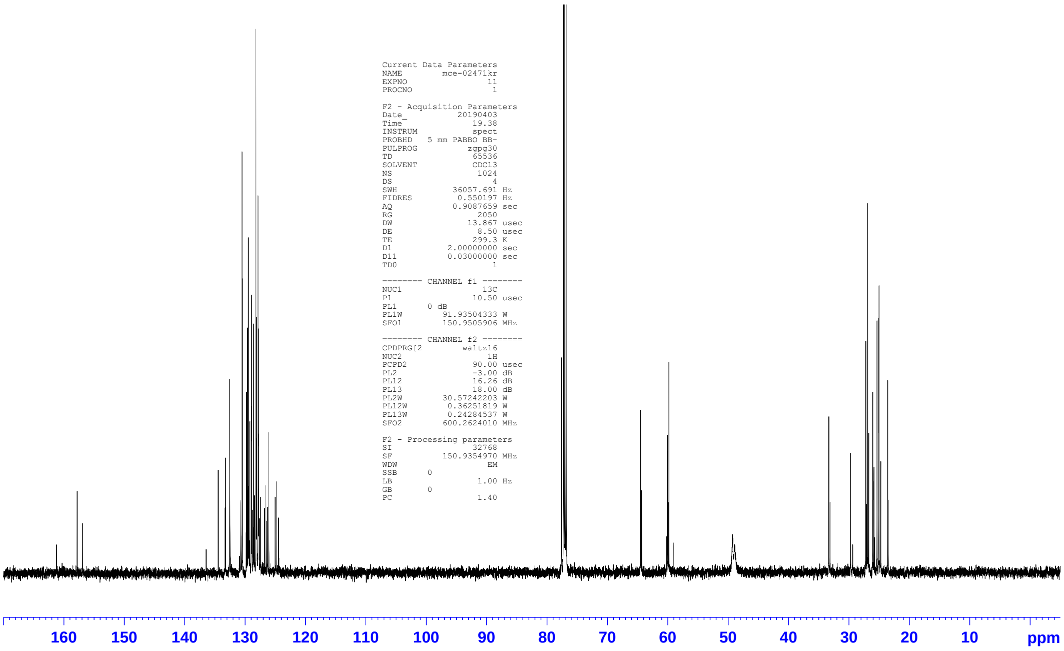


**Figure S48**. $^{13}$C NMR of (*R*,*R*)-**9d** ($CDCl_3$, 151 MHz).

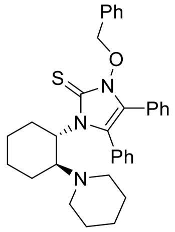


(*S*,*S*)-**9d**

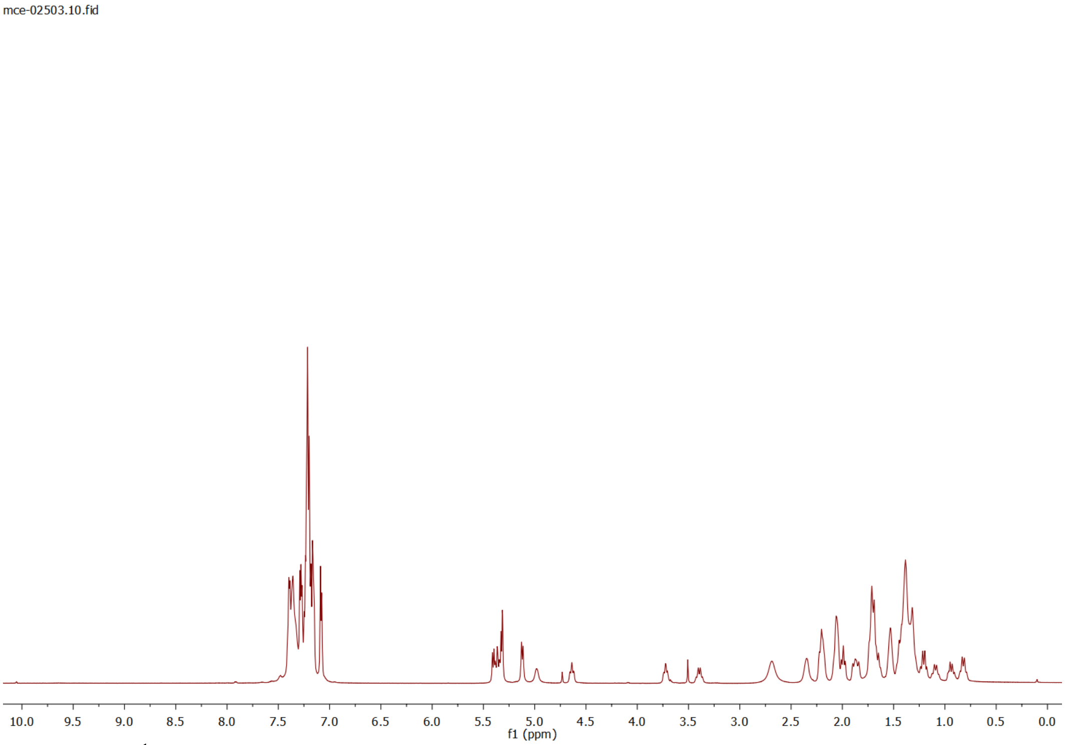


**Figure S49**. $^{1}$H NMR of (*S*,*S*)-**9d** ($CDCl_3$, 600 MHz).

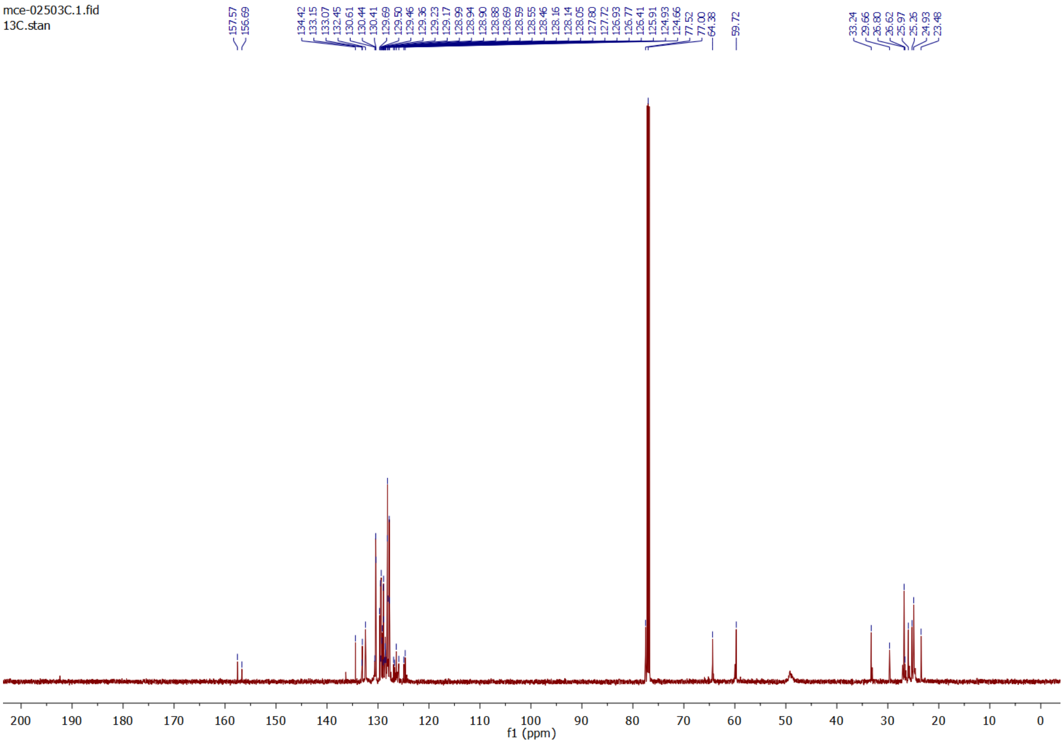


**Figure S50**. $^{13}$C NMR of (*S*,*S*)-**9d** ($CDCl_3$, 151 MHz).

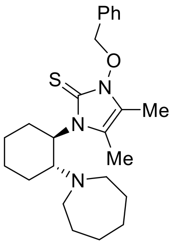


(*R*,*R*)-**9e**

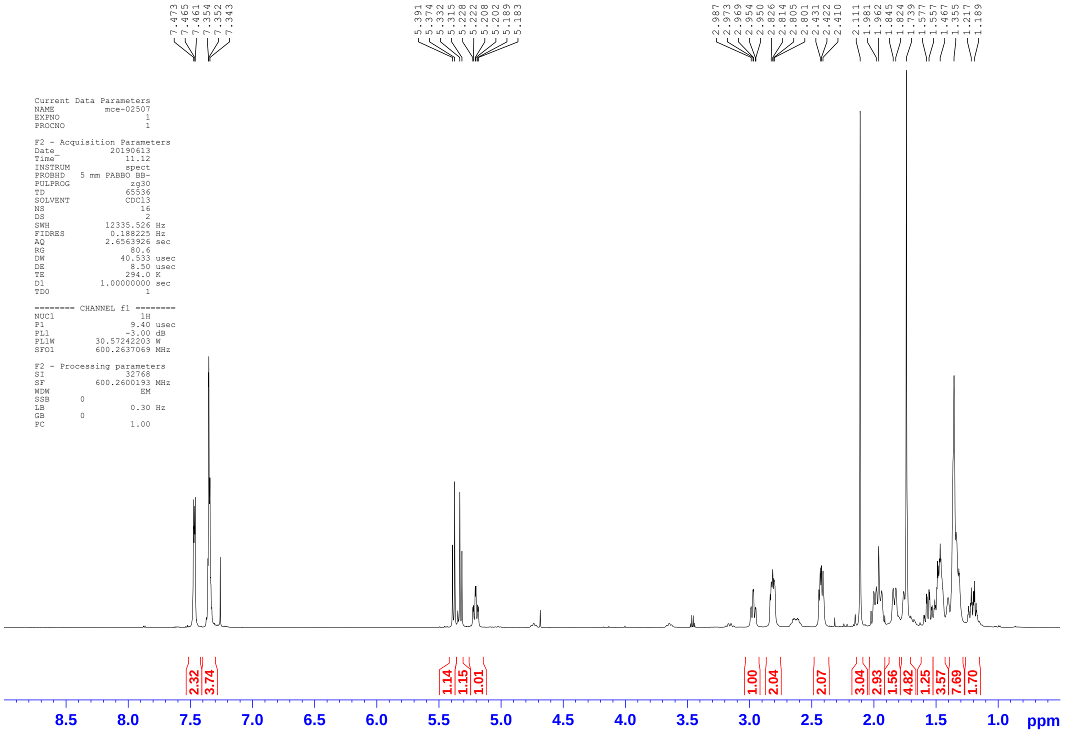


**Figure S51**. $^{1}$H NMR of (*R*,*R*)-**9e** ($CDCl_3$, 600 MHz).

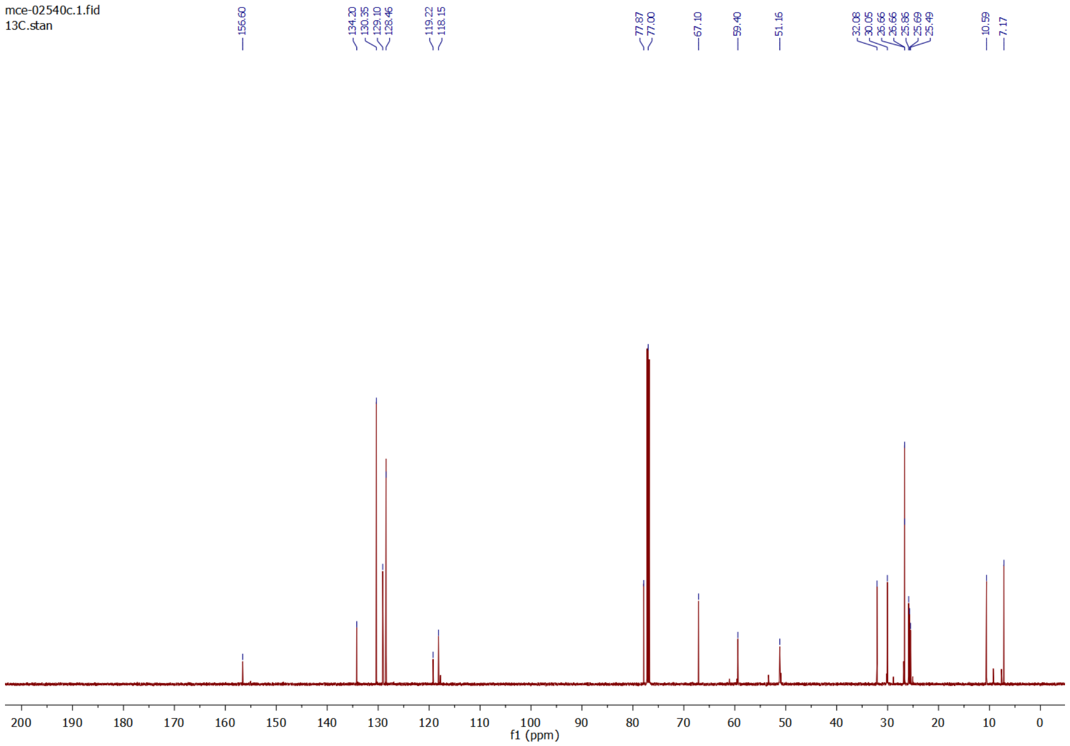


**Figure S52**. $^{13}$C NMR of (*R*,*R*)-**9e** ($CDCl_3$, 151 MHz).

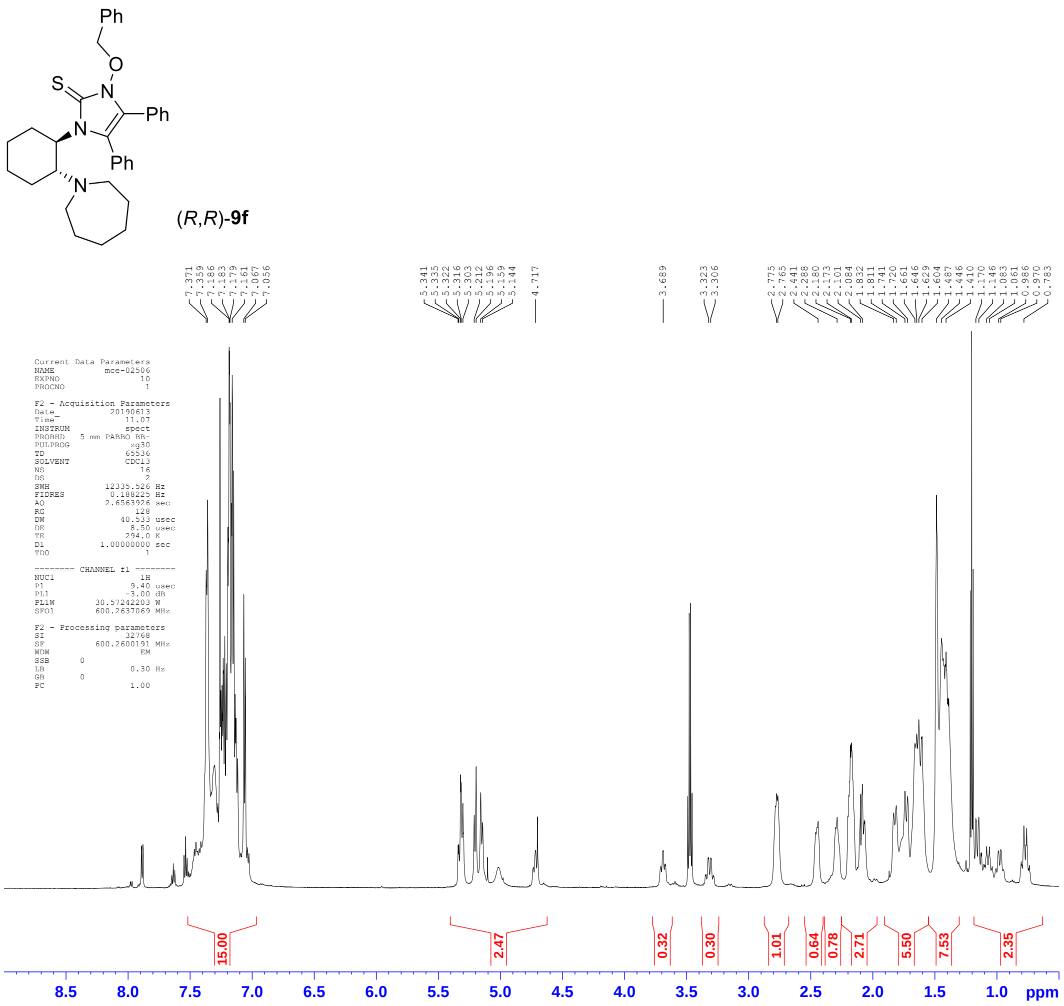

**Figure S53**. $^{1}$H NMR of (*R*,*R*)-**9f** ($CDCl_3$, 600 MHz).

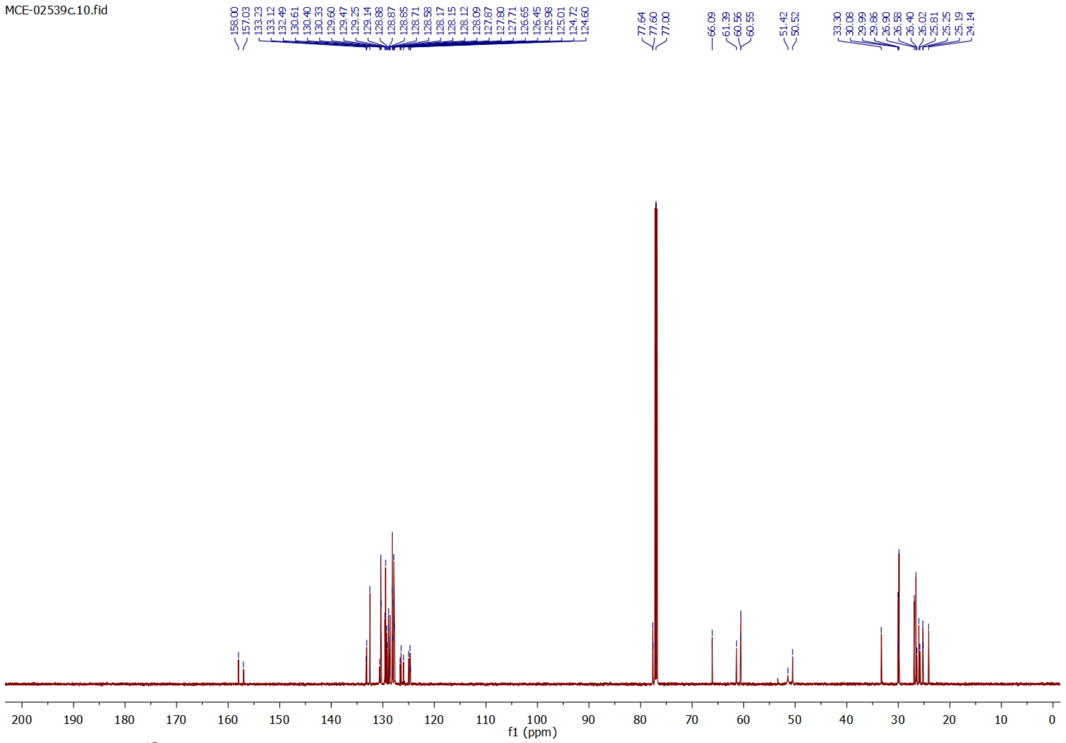

**Figure S54**. $^{13}$C NMR of (*R*,*R*)-**9f** ($CDCl_3$, 151 MHz).

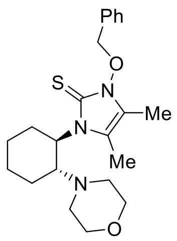


(*R*,*R*)-**9g**

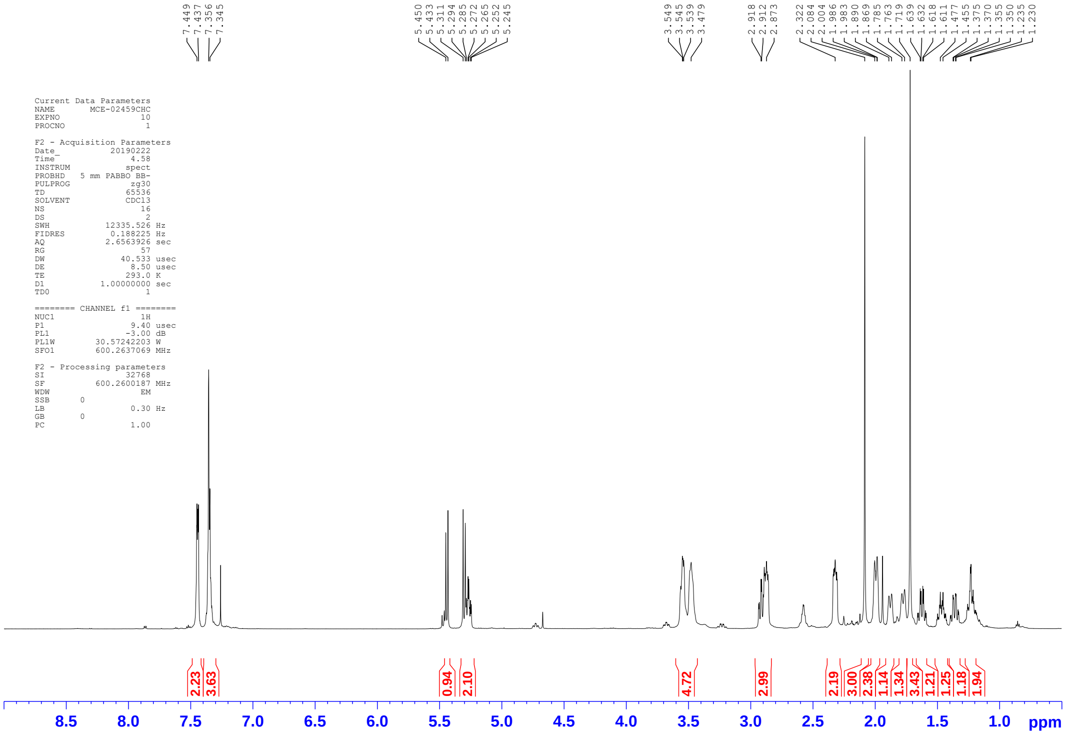


**Figure S55**. $^{1}H$ NMR of (*R*,*R*)-**9g** ($CDCl_3$, 600 MHz).

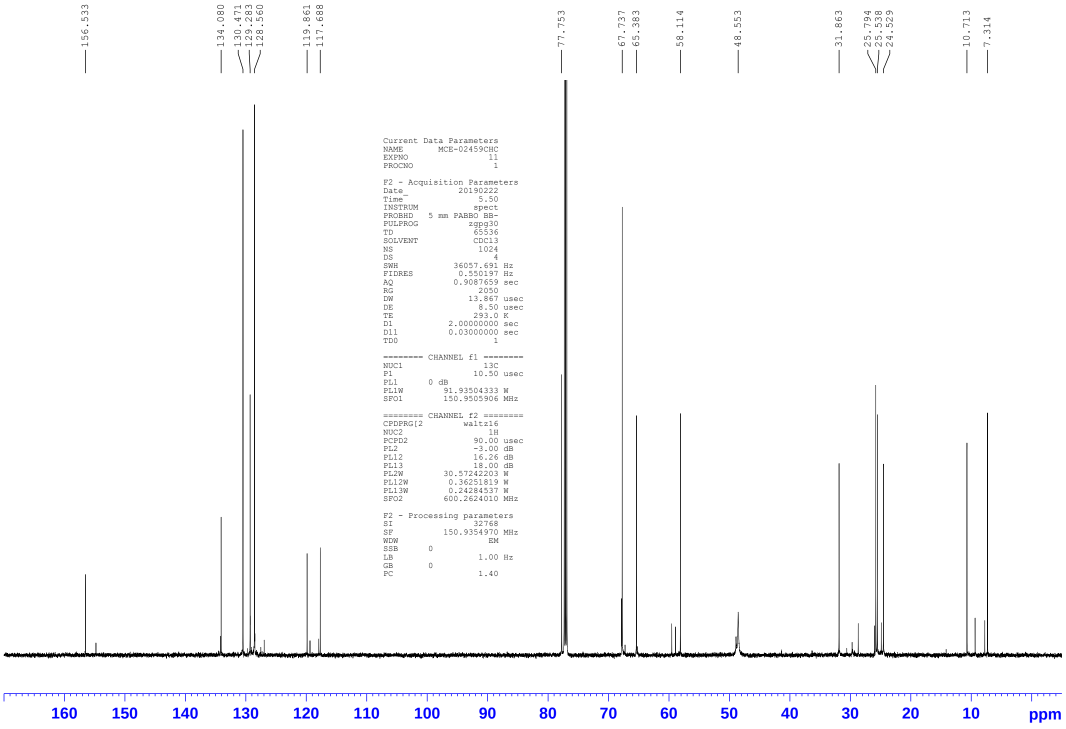


**Figure S56**. $^{13}C$ NMR of (*R*,*R*)-**9g** ($CDCl_3$, 151 MHz).

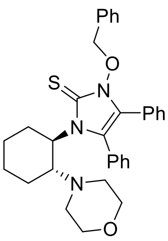


(*R*,*R*)-**9h**

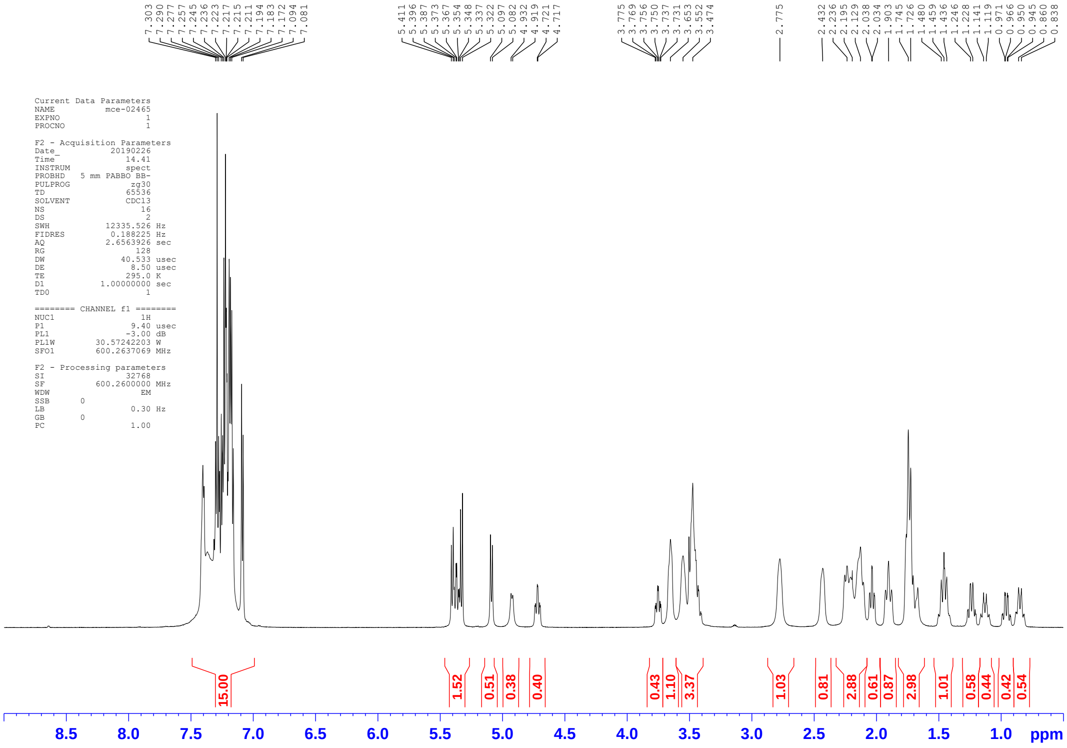


**Figure S57**. $^{1}H$ NMR of (*R*,*R*)-**9h** ($CDCl_3$, 600 MHz).

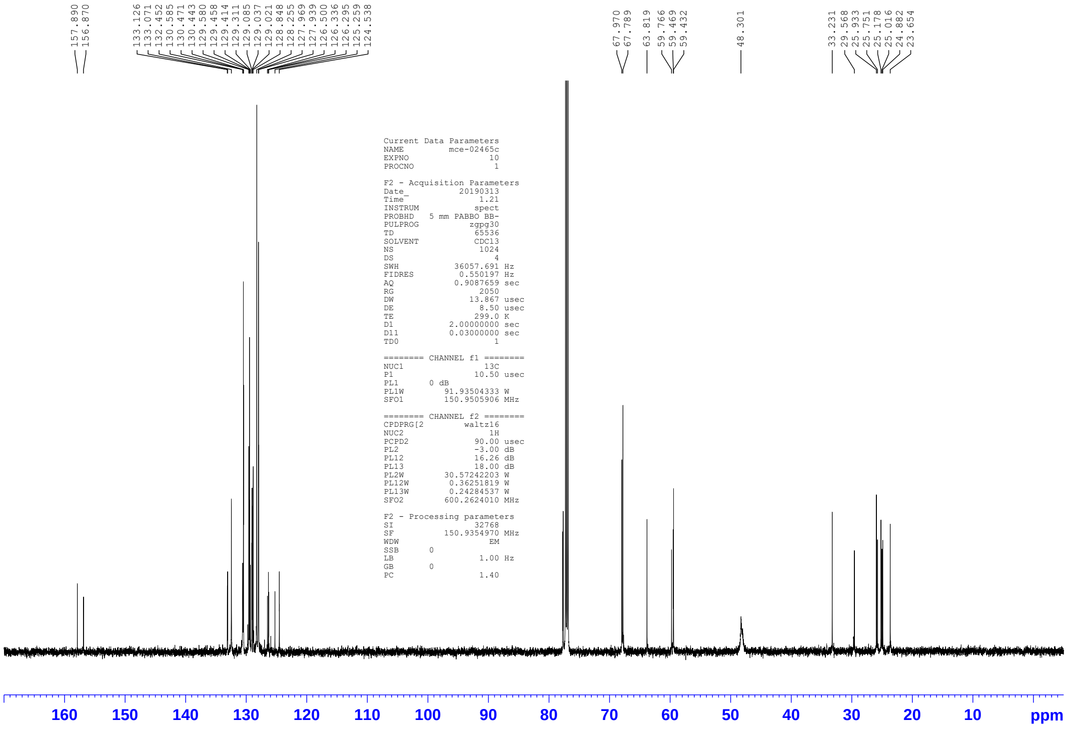


**Figure S58**. $^{13}C$ NMR of (*R*,*R*)-**9h** ($CDCl_3$, 151 MHz).

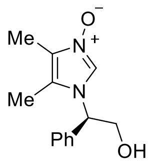


**10b**

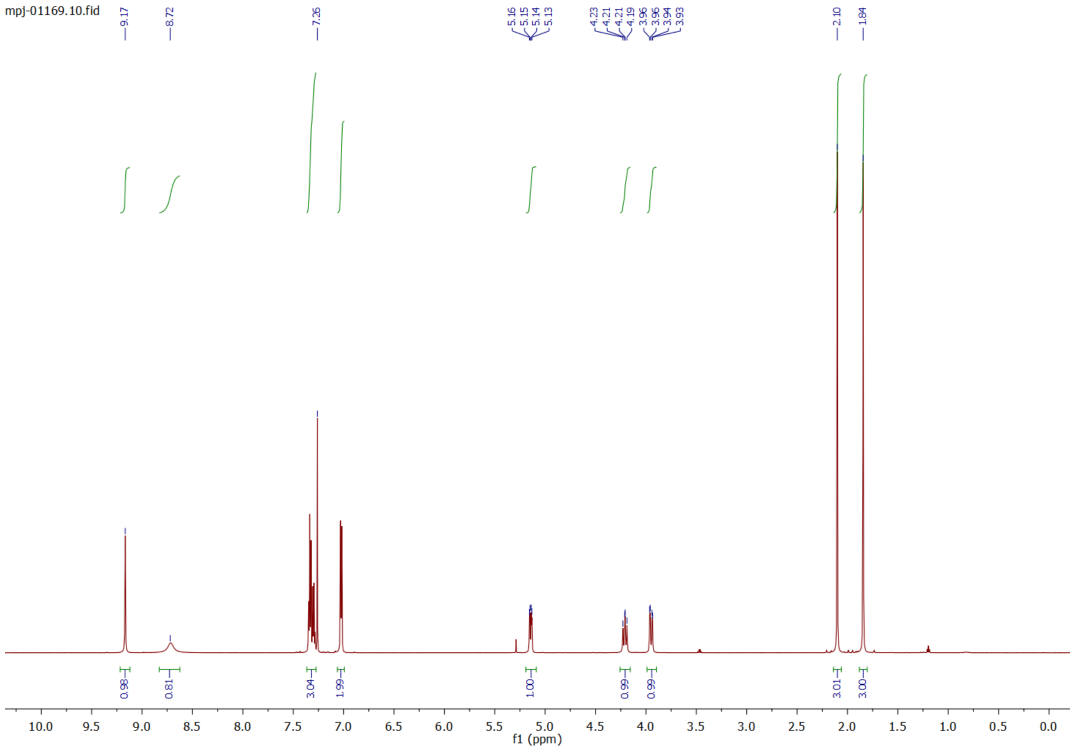


**Figure S59**. $^{1}$H NMR of **10b** ($CDCl_3$, 600 MHz).

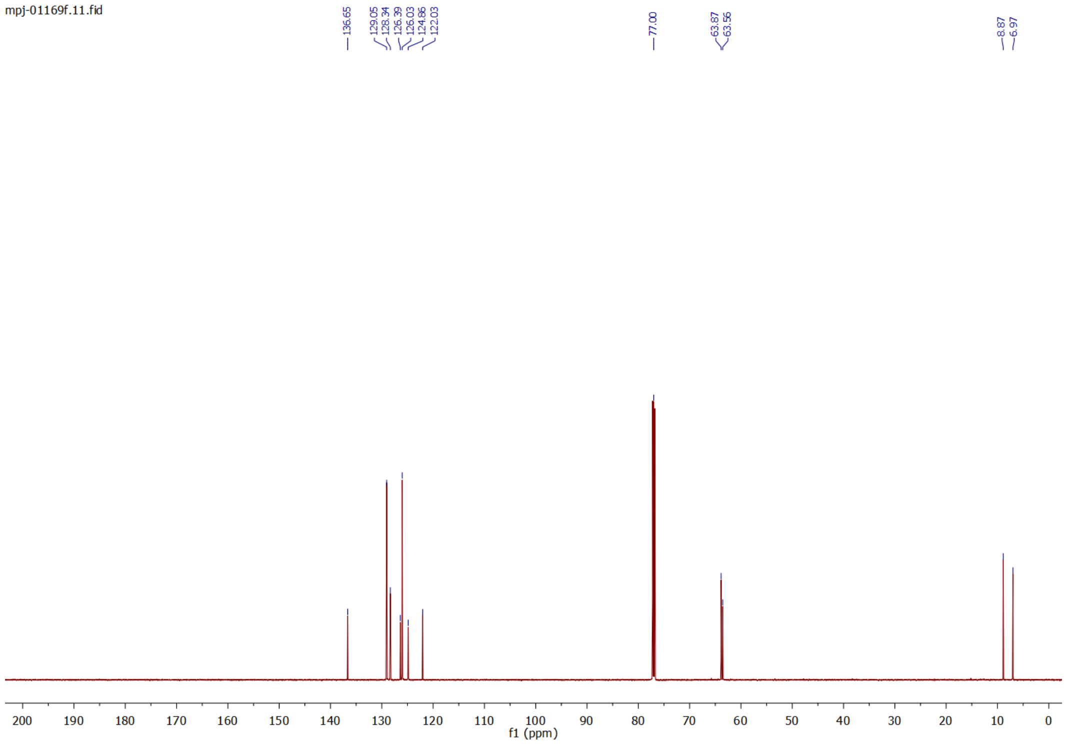


**Figure S60**. $^{13}$C NMR of **10b** ($CDCl_3$, 151 MHz).

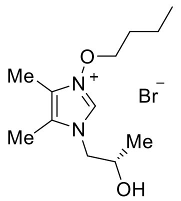


**11a**

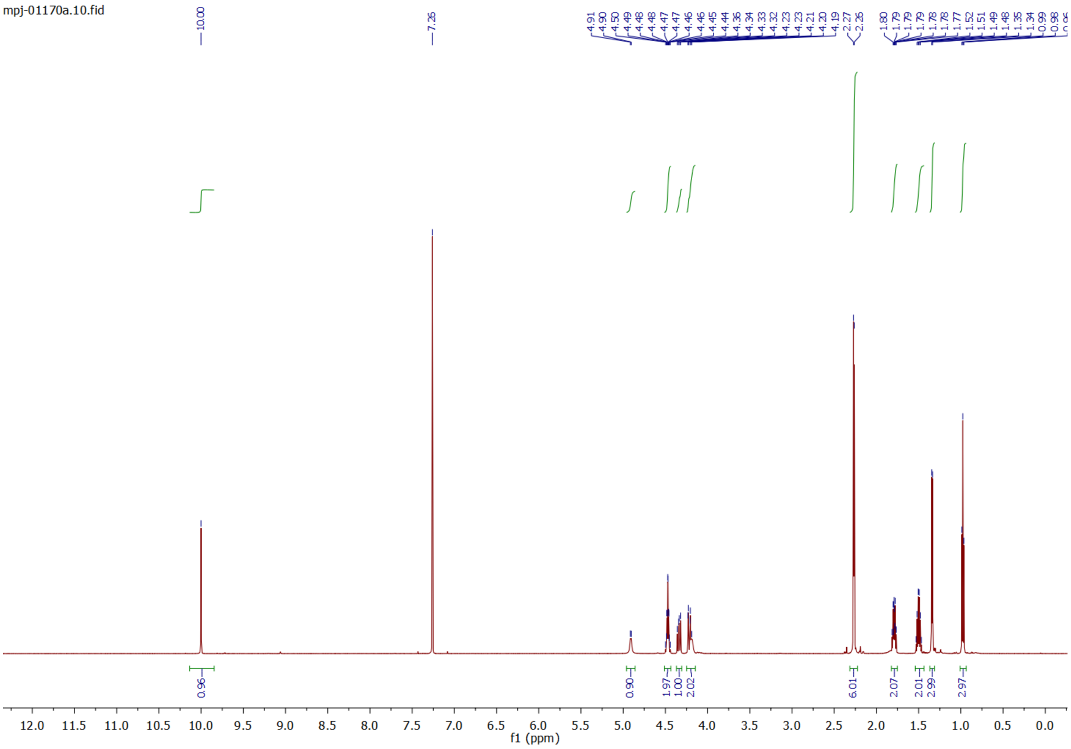


**Figure S61**. $^{1}$H NMR of **11a** ($CDCl_3$, 600 MHz).

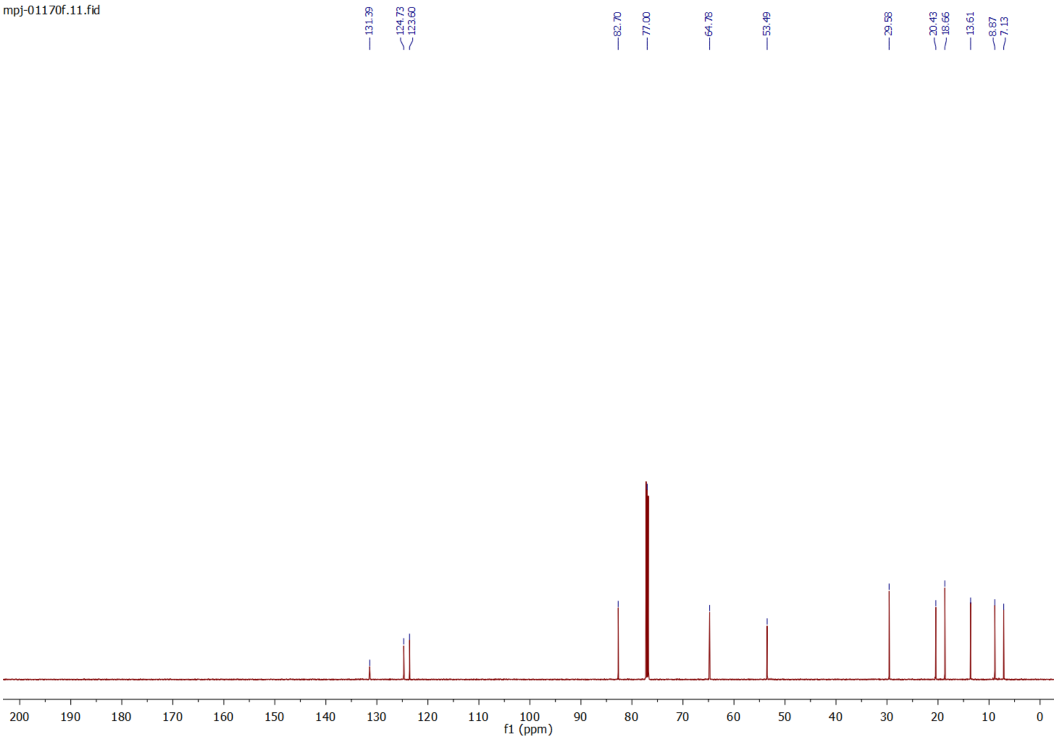


**Figure S62**. $^{13}$C NMR of **11a** ($CDCl_3$, 151 MHz).

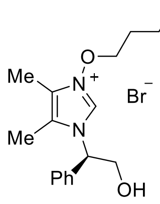


**11b**

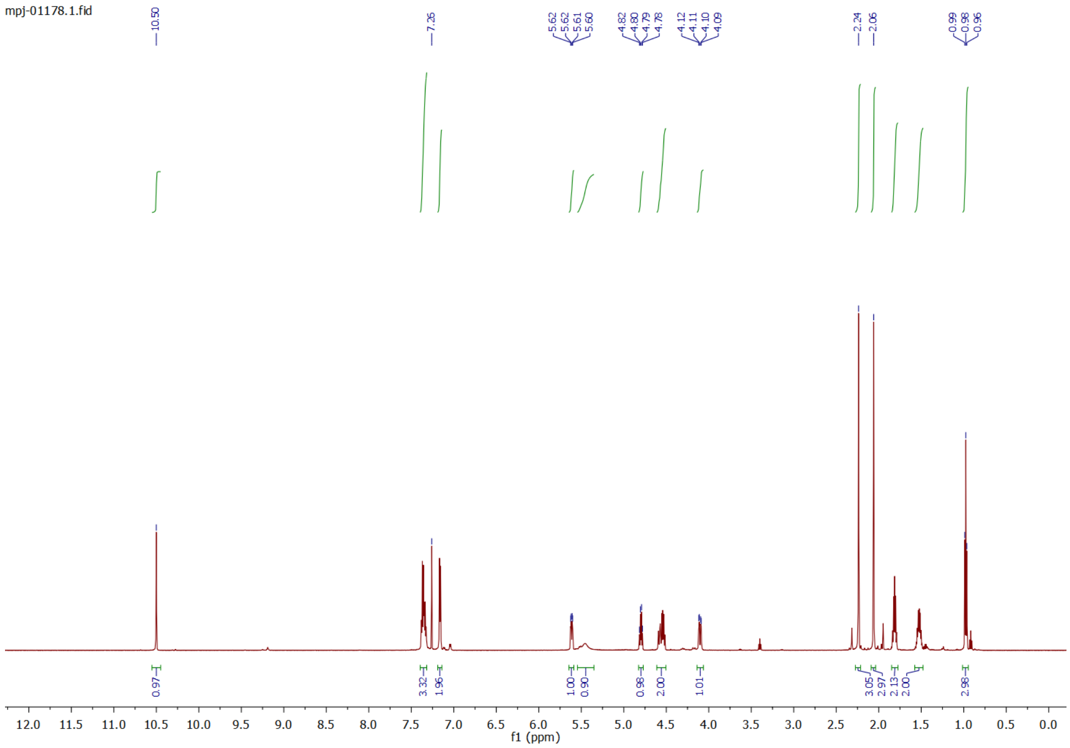


**Figure S63**. $^{1}$H NMR of **11b** ($CDCl_3$, 600 MHz).

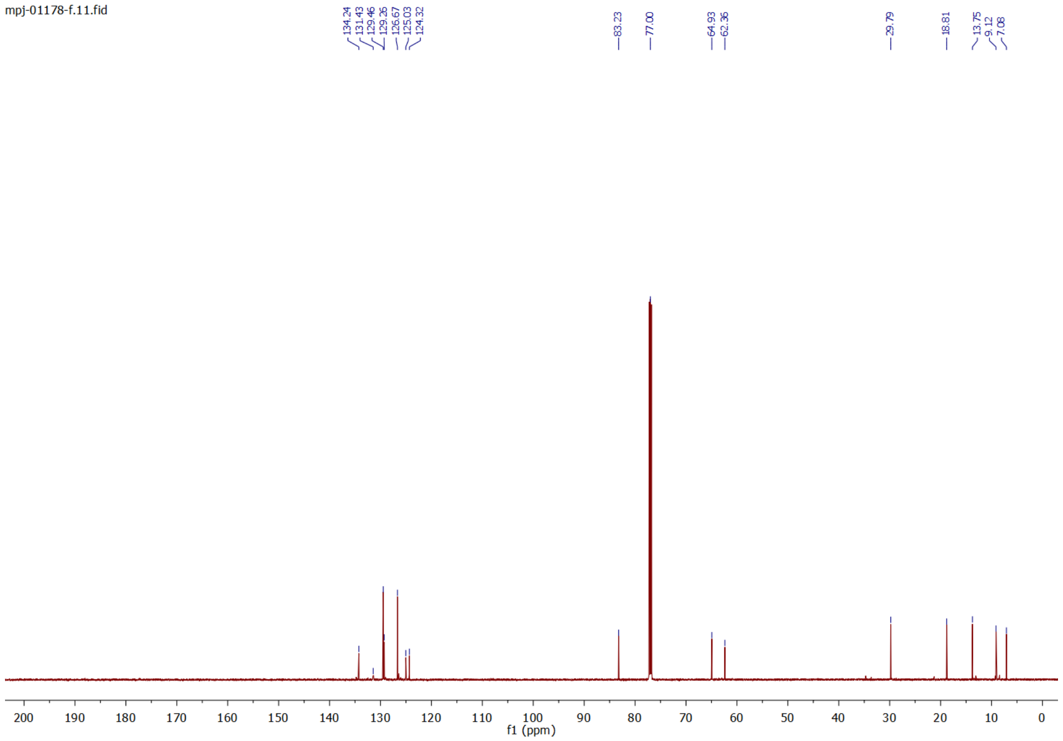


**Figure S64**. $^{13}$C NMR of **11b** ($CDCl_3$, 151 MHz).

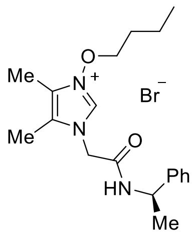


**11d**

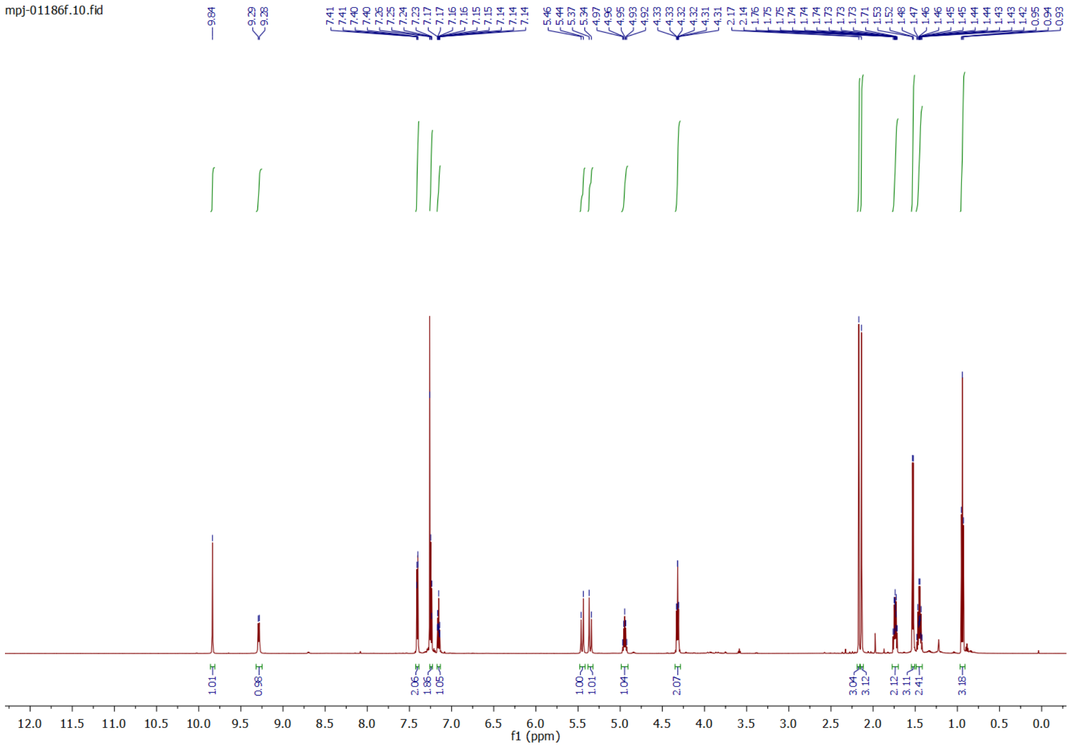


**Figure S65**. $^{1}$H NMR of **11d** ($CDCl_3$, 600 MHz).

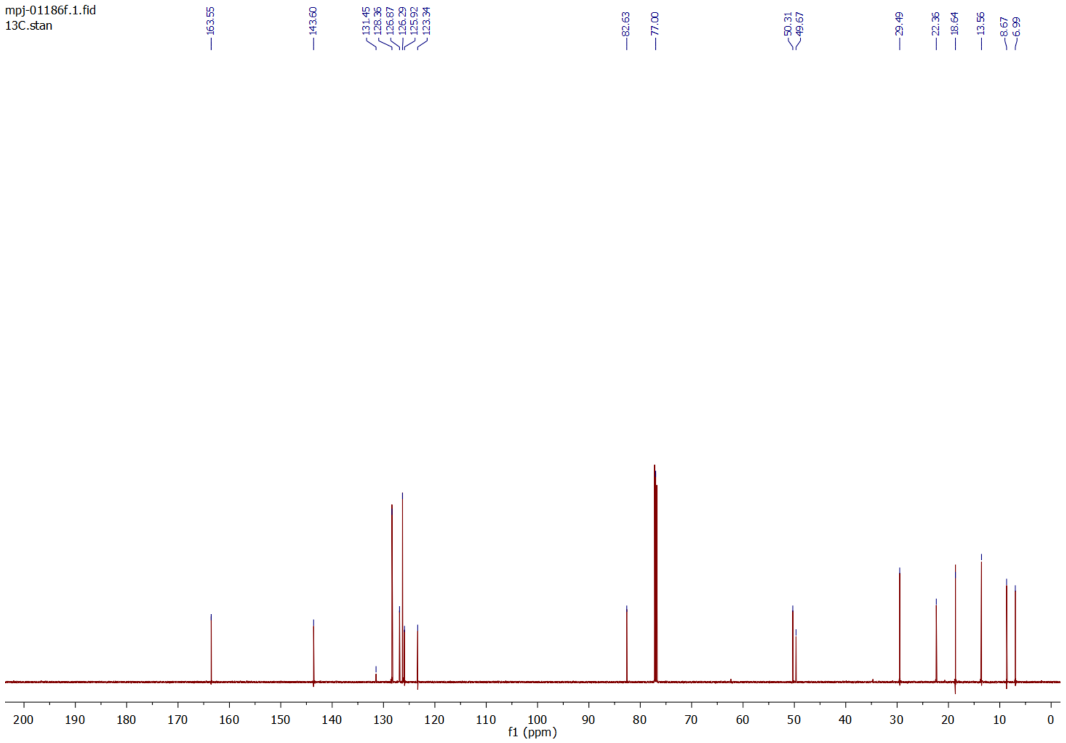


**Figure S66**. $^{13}$C NMR of **11d** ($CDCl_3$, 151 MHz).

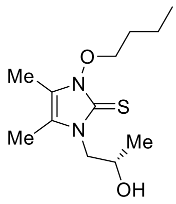


**13a**

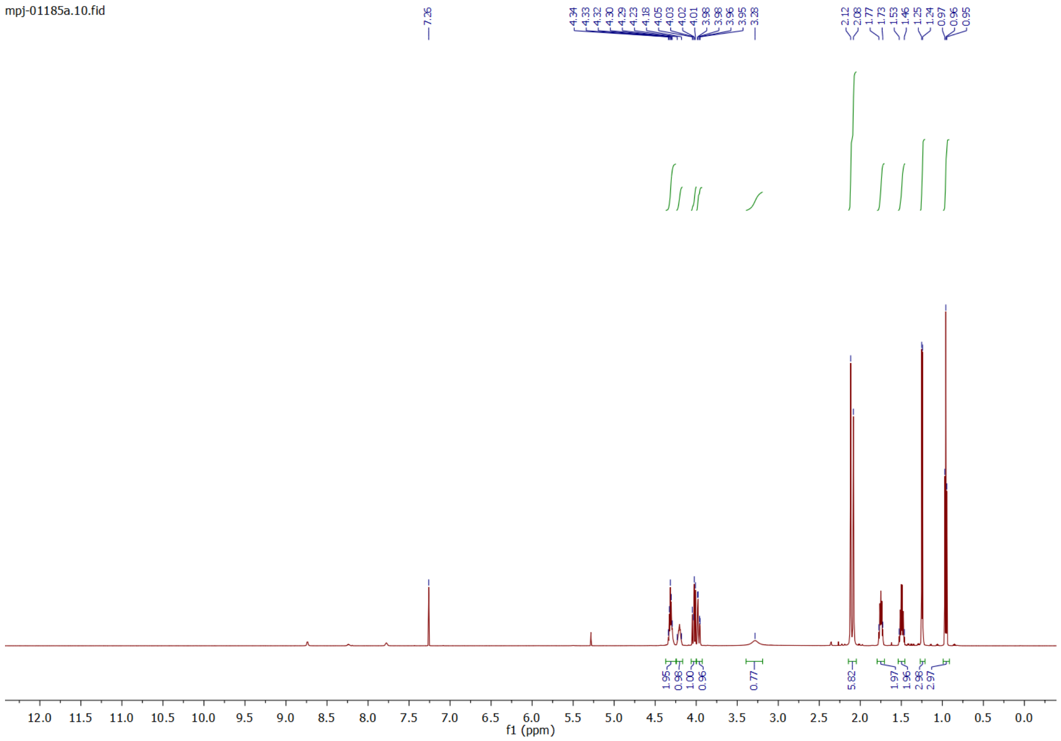


**Figure S67**. $^{1}$H NMR of **13a** ($CDCl_3$, 600 MHz).

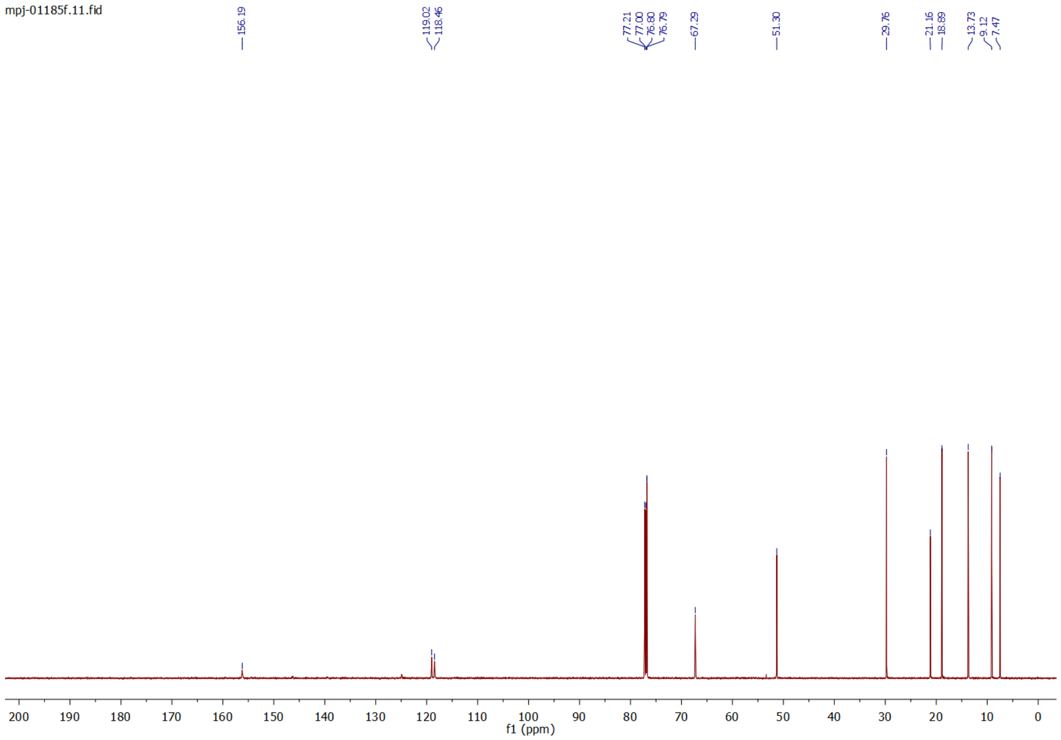


**Figure S68**. $^{13}$C NMR of **13a** ($CDCl_3$, 151 MHz).

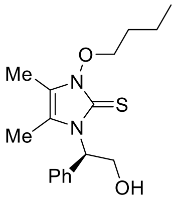


**13b**

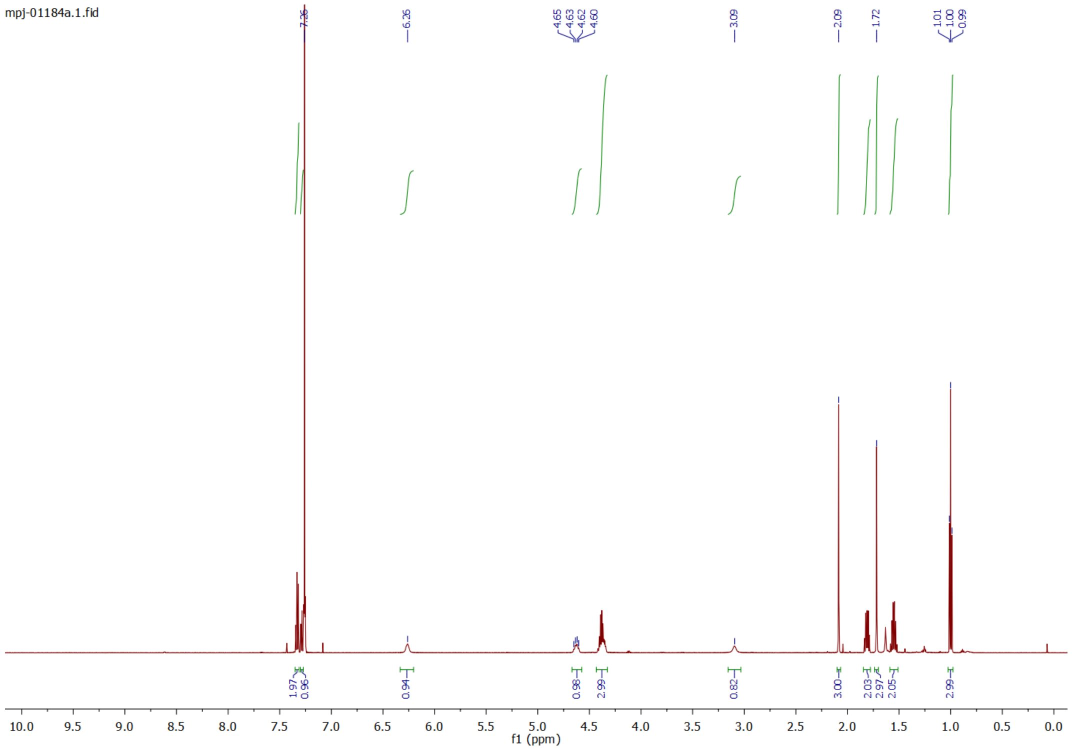


**Figure S69**. $^{1}$H NMR of **13b** ($CDCl_3$, 600 MHz).

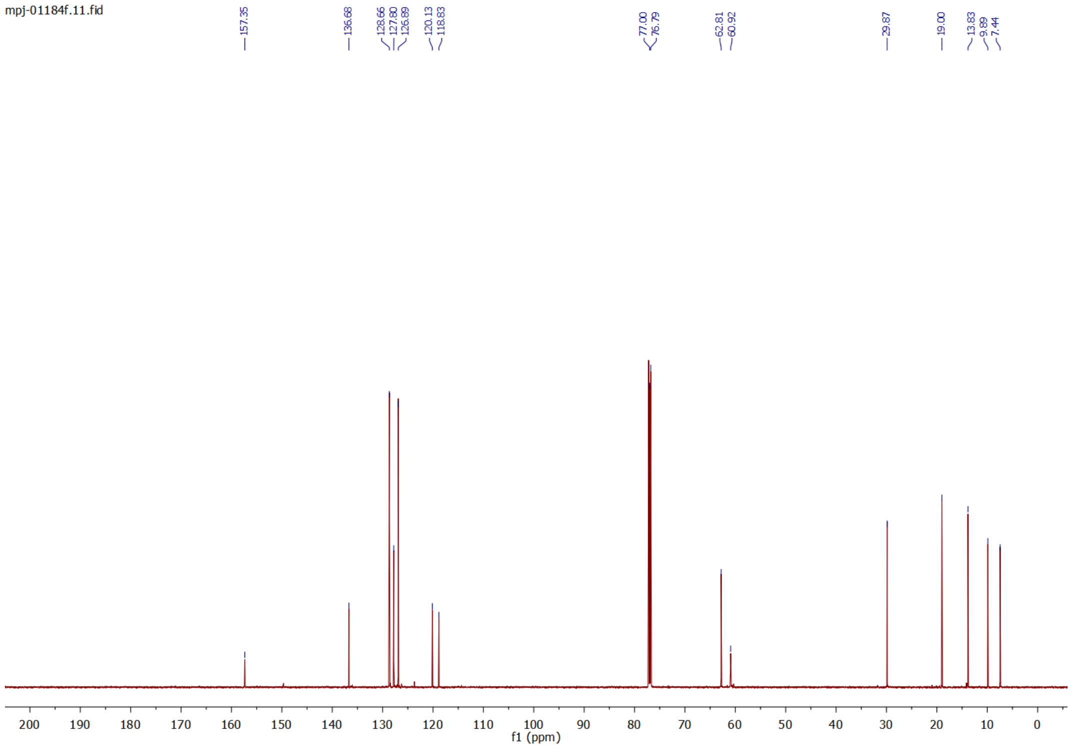


**Figure S70**. $^{13}$C NMR of **13b** ($CDCl_3$, 151 MHz).

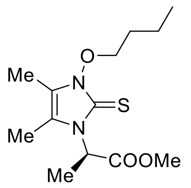


**13c**

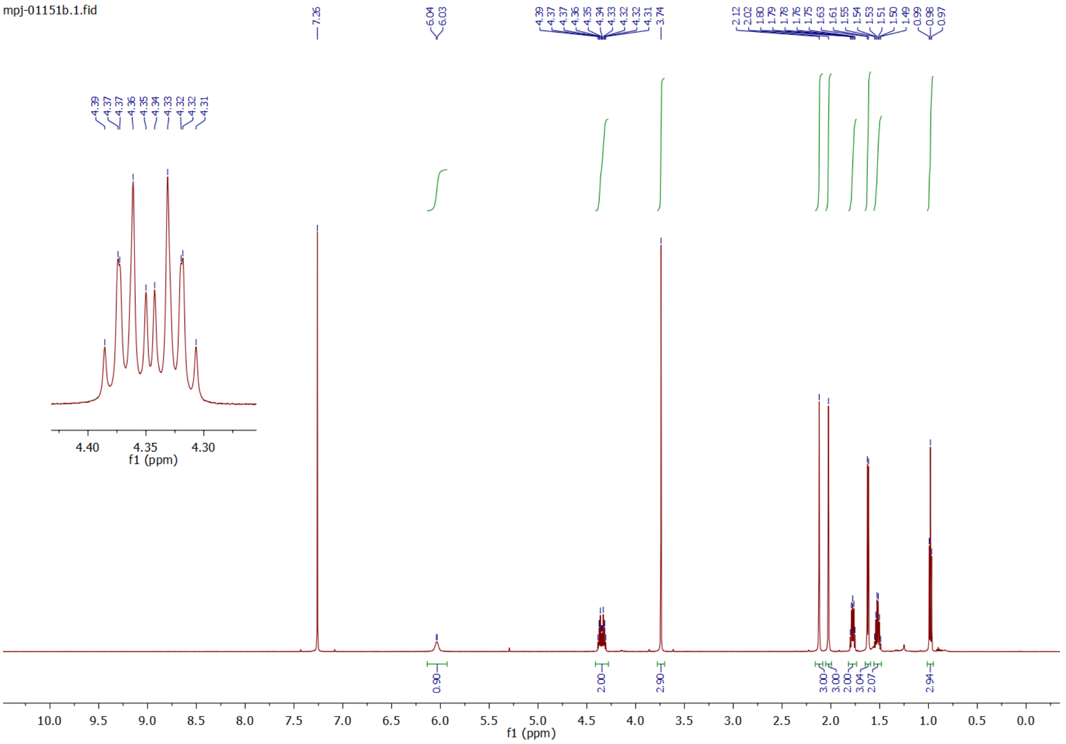


**Figure S71**. $^{1}$H NMR of **13c** ($CDCl_3$, 600 MHz).

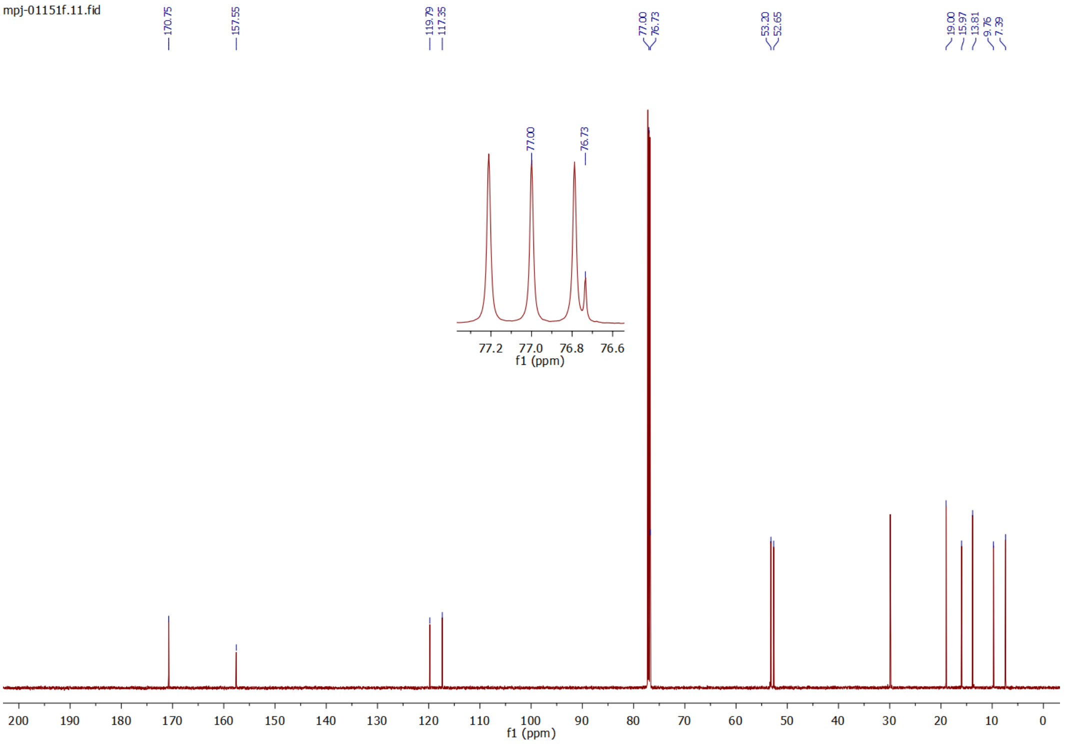


**Figure S72**. $^{13}$C NMR of **13c** ($CDCl_3$, 151 MHz).

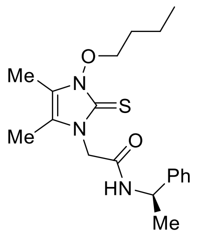

**13d**

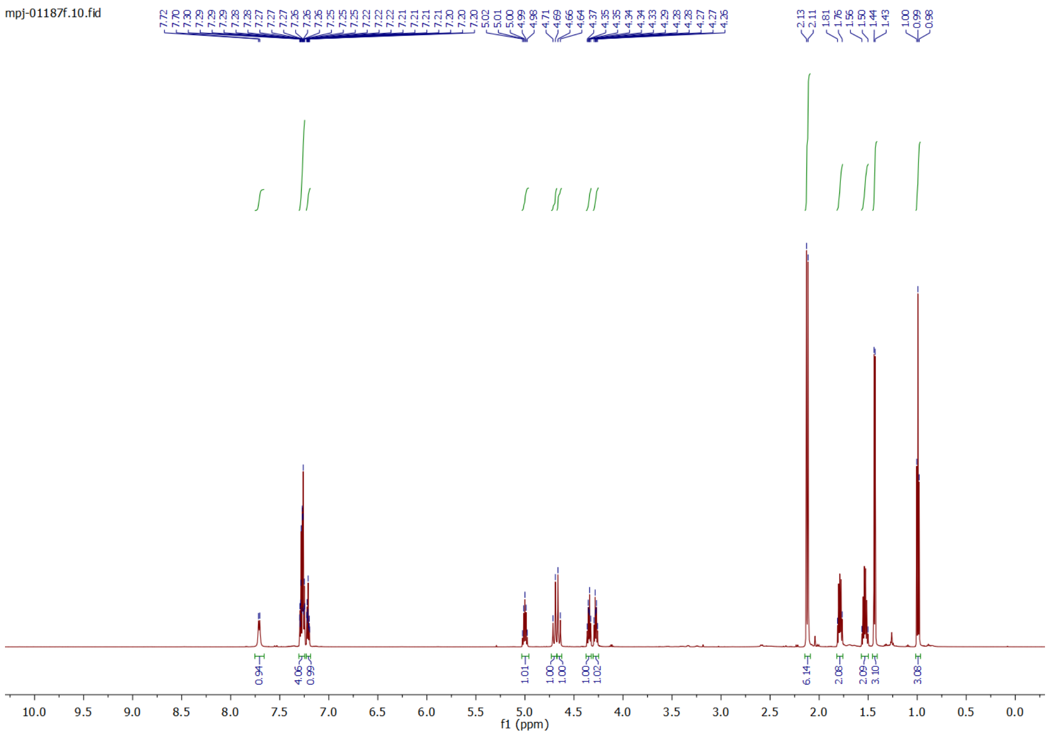

**Figure S73**. $^{1}$H NMR of **13d** ($CDCl_3$, 600 MHz).

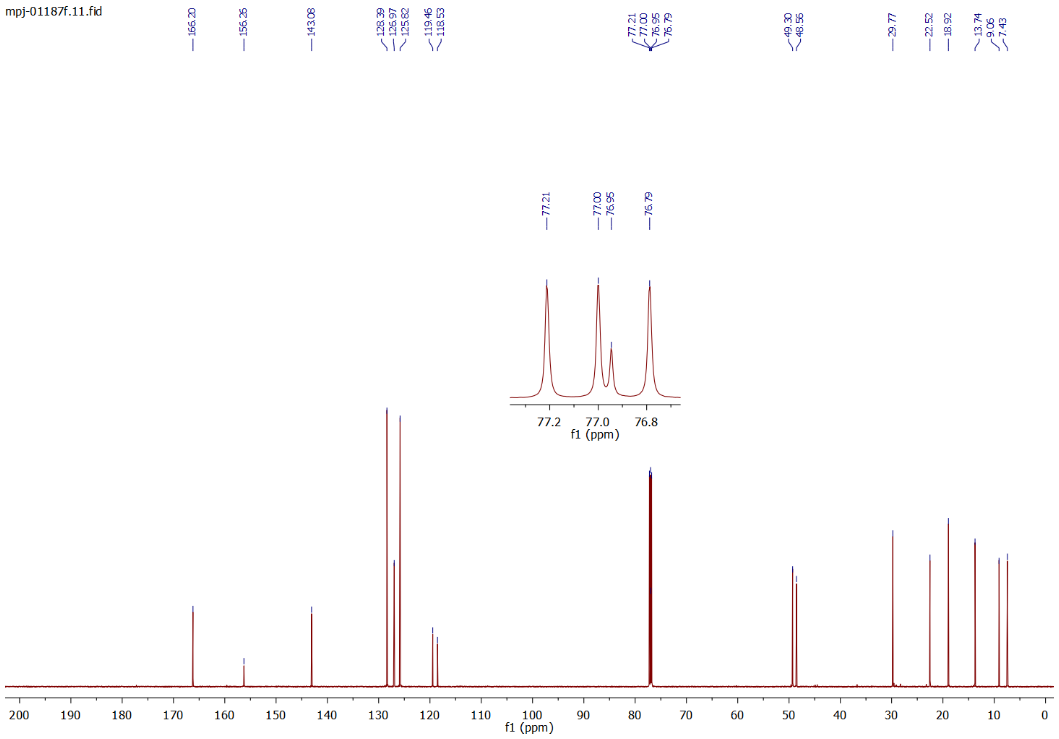

**Figure S74**. $^{13}$C NMR of **13d** ($CDCl_3$, 151 MHz).

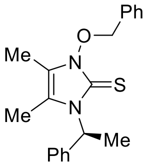


**15a**

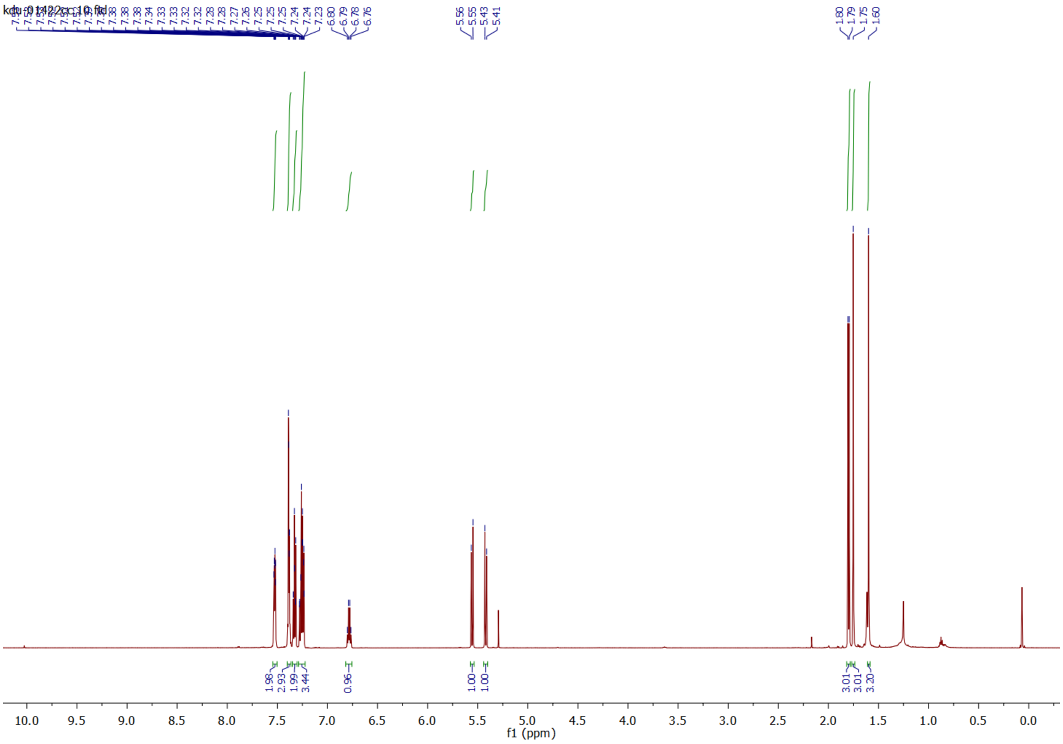


**Figure S75**. $^{1}H$ NMR of **15a** ($CDCl_3$, 600 MHz).

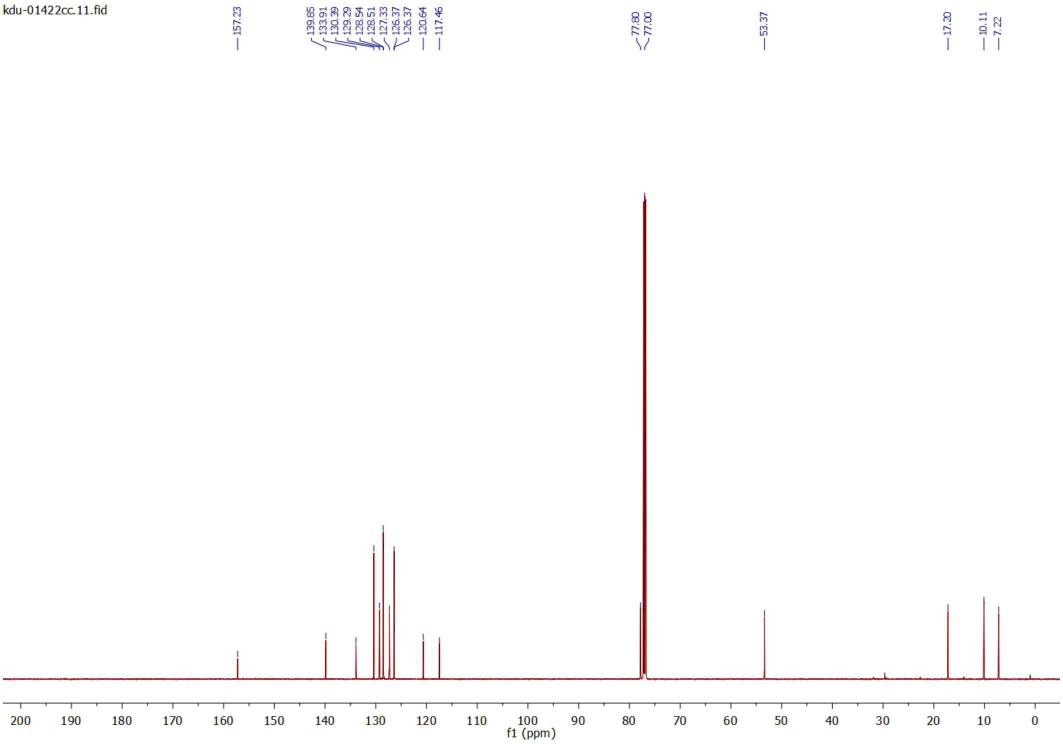


**Figure S76**. $^{13}C$ NMR of **15a** ($CDCl_3$, 151 MHz).

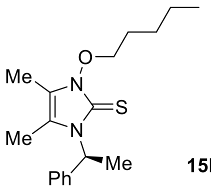


**15b**

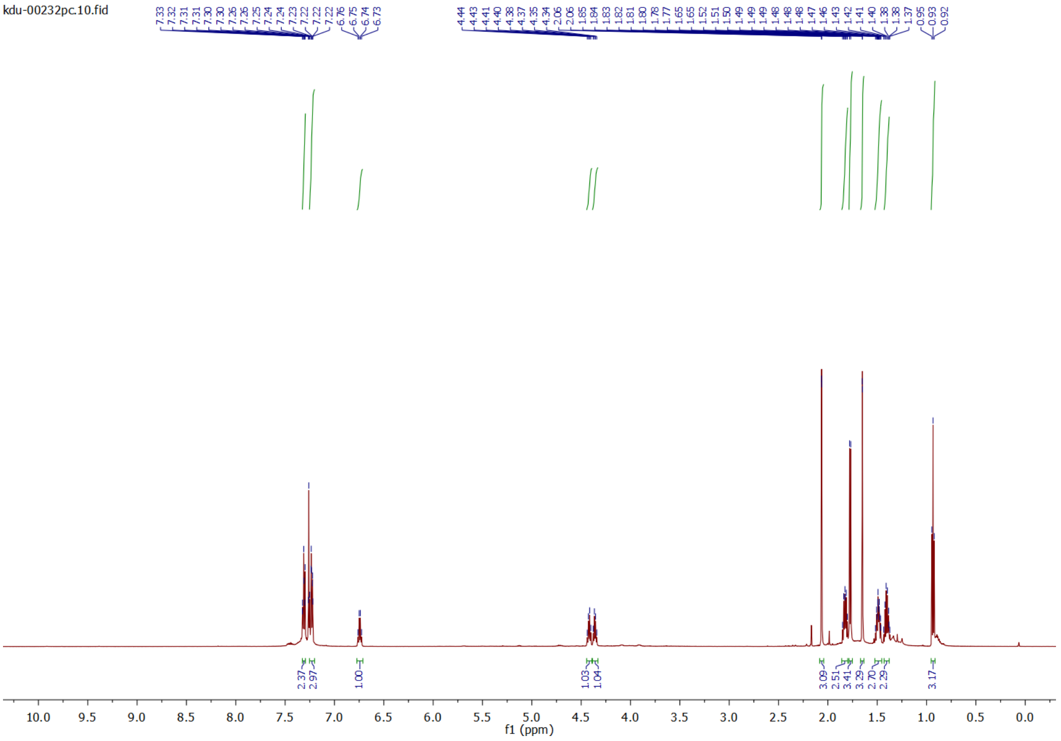


**Figure S77**. $^{1}$H NMR of **15b** ($CDCl_3$, 600 MHz).

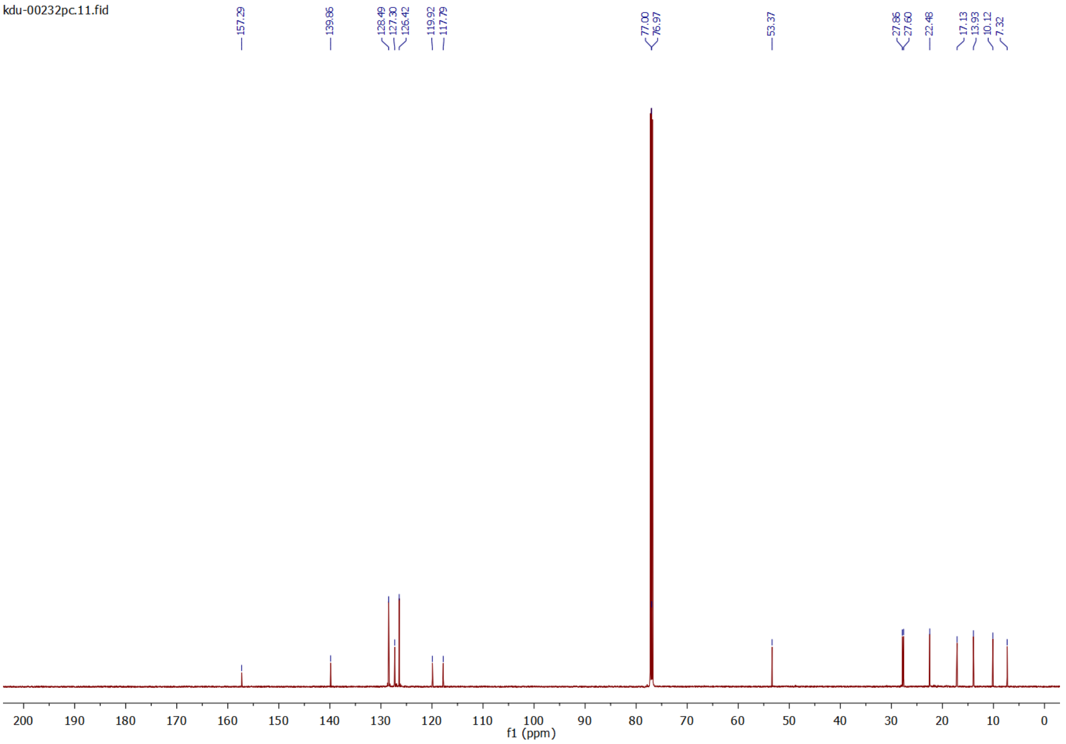


**Figure S78**. $^{13}$C NMR of **15b** ($CDCl_3$, 151 MHz).

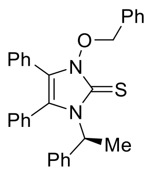


**15c**

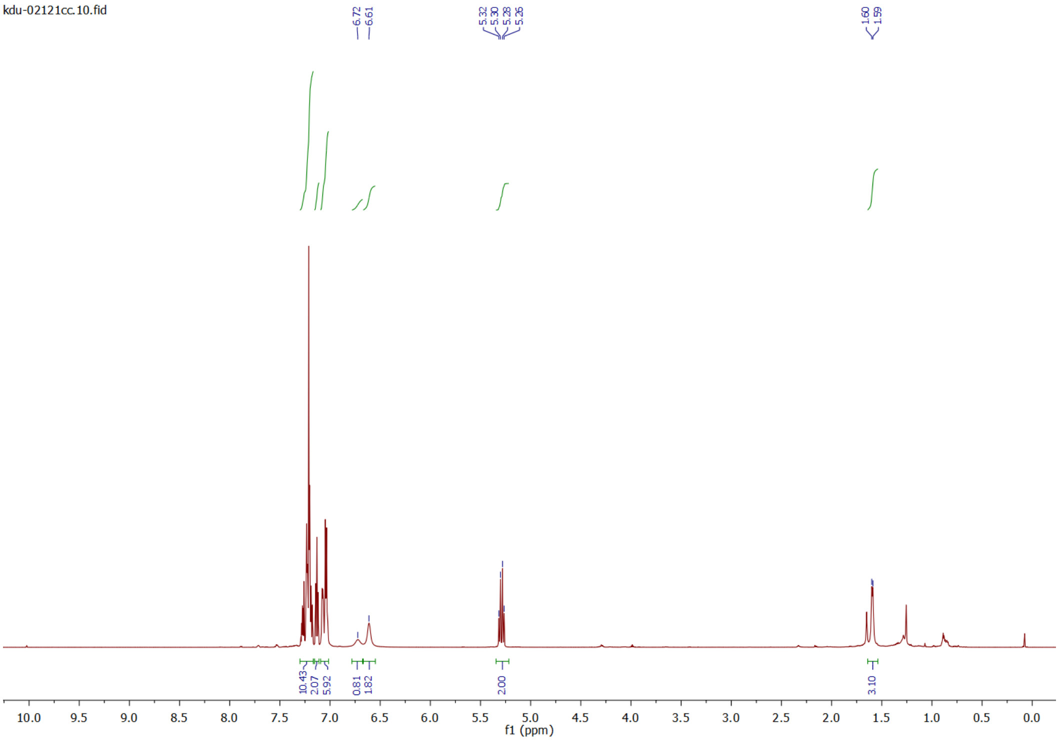


**Figure S79**. $^{1}$H NMR of **15c** ($CDCl_3$, 600 MHz).

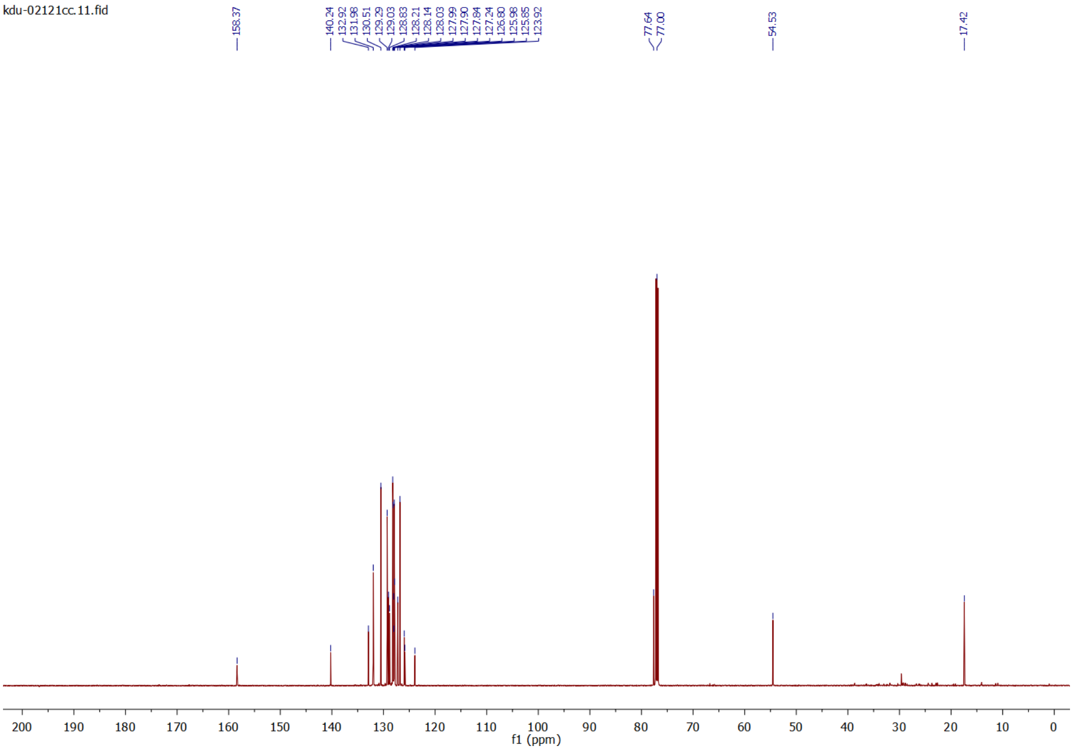


**Figure S80**. $^{13}$C NMR of **15c** ($CDCl_3$, 151 MHz).

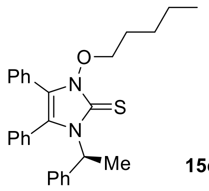


**15d**

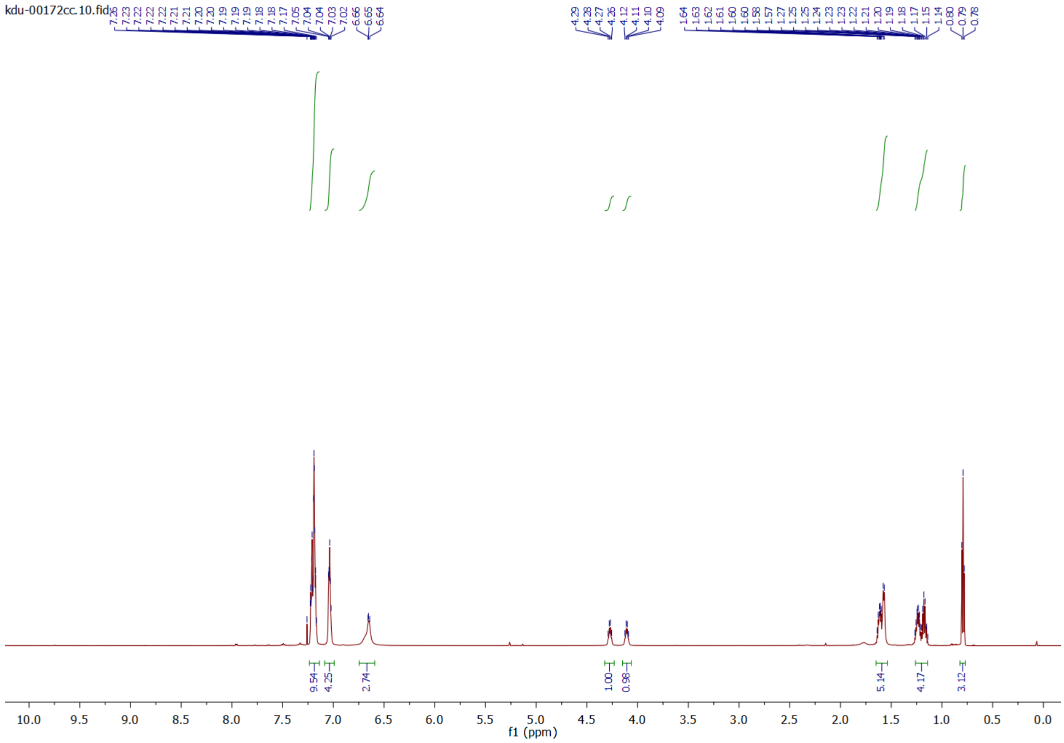


**Figure S81**. $^{1}$H NMR of **15d** ($CDCl_3$, 600 MHz).

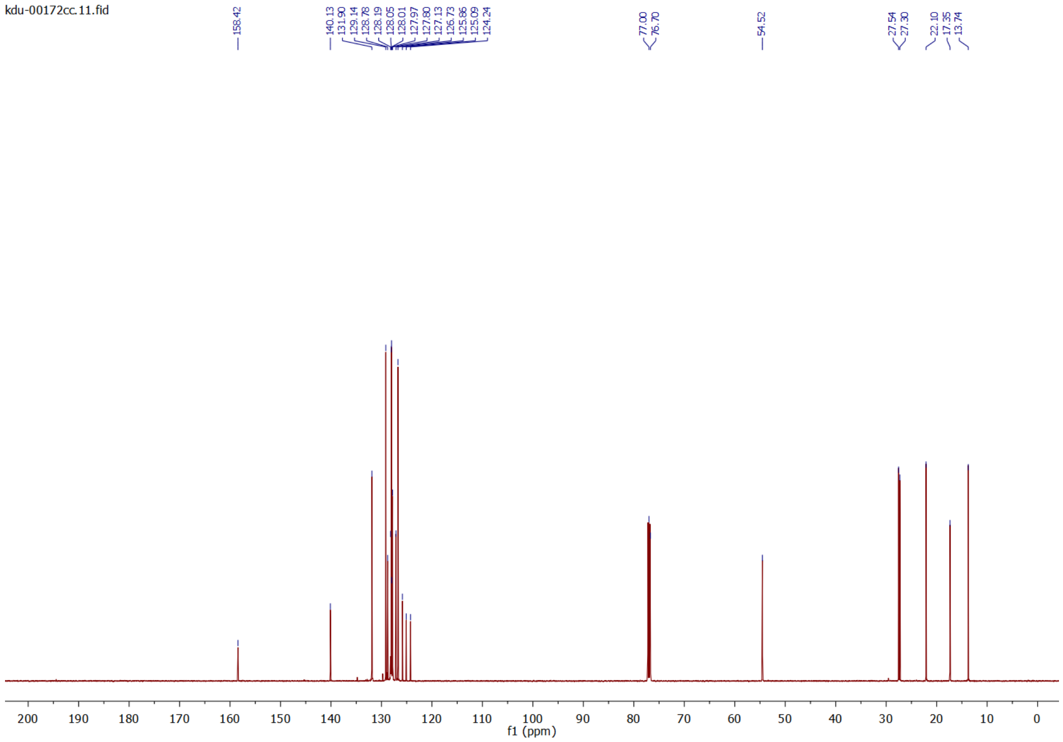


**Figure S82**. $^{13}$C NMR of **15d** ($CDCl_3$, 151 MHz).
